# Ultrafast structural dynamics of carbon–carbon single-bond rotation in transient radical species at non-equilibrium

**DOI:** 10.1038/s41467-025-57279-7

**Published:** 2025-02-25

**Authors:** Seonggon Lee, Hosung Ki, Donghwan Im, Jungmin Kim, Yunbeom Lee, Jain Gu, Alekos Segalina, Jun Heo, Yongjun Cha, Kyung Won Lee, Doyeong Kim, Jeongho Kim, Rory Ma, Jae Hyuk Lee, Hyotcherl Ihee

**Affiliations:** 1 Center for Advanced Reaction Dynamics, Institute for Basic Science (IBS), Daejeon, 34141 Republic of Korea; 2 Department of Chemistry and KI for the BioCentury, Korea Advanced Institute of Science and Technology (KAIST), Daejeon, 34141 Republic of Korea; 3 Department of Chemistry, Inha University, 100 Inha-ro, Michuhol-gu, Incheon, 22212 Republic of Korea; 4 Pohang Accelerator Laboratory, Pohang, Gyeongbuk 37673 Republic of Korea

**Keywords:** Reaction kinetics and dynamics, Thermodynamics, Stereochemistry, Structure prediction, Chemical physics

## Abstract

Bond rotation is an important phenomenon governing the fate of reactions. In particular, heterogeneously substituted ethane derivatives provide distinct structural conformations around the bond, empowering them as ideal systems for studying the rotation along carbon-containing single bonds. However, structural dynamics of ultrafast single-bond rotation, especially along C–C• bonds, have remained elusive as tracking the detailed changes in structural parameters during the rotational isomerization is challenging with conventional spectroscopic tools. Here, we employ femtosecond time-resolved X-ray liquidography to visualize the rotational isomerization between *anti* and *gauche* conformers of tetrafluoroiodoethyl radical (C_2_F_4_I•) and 1,2-tetrafluorodiiodoethane (C_2_F_4_I_2_), simultaneously. The TRXL data captures perturbations in conformer ratios and structures of each reacting species, revealing that the rotational isomerization of C_2_F_4_I• and C_2_F_4_I_2_ follows *anti*-to-*gauche* and *gauche*-to-*anti* paths with time constants of 1.2 ps and 26 ps, respectively. These findings also align with the computational predictions. This work offers an atomic-level insight into the kinetics and structural dynamics of single-bond rotation.

## Abstract (web summary)

Carbon–carbon single-bond rotation is a fundamental process in organic molecules. Here the authors use time-resolved X-ray liquidography to follow this motion in a haloalkane and its photodissociation product on an ultrafast timescale with atomic resolution.

## Introduction

The internal rotation of molecules is foundational in chemistry, governing the reactivity, stereochemistry, and fate of reactions^1^. In particular, the rotation of single bonds containing carbon atoms plays an indispensable role in synthetic and biological chemistry. The rotational degree of freedom around carbon–carbon (C–C) single bonds provides flexibility to the three-dimensional orientation of atoms in a molecule, giving rise to the concept of conformational isomerism. The conformational isomerism determines the stereochemistry of the products in some key reactions^2,3^ and affects the three-dimensional structure of proteins by altering interactions between residues^4–7^. Therefore, understanding the chemical origins, structural dynamics, and potentials of manipulation for the internal single-bond rotation processes is vital for synthesizing complex molecules with desired functionalities, improving the adaptability of proteins, and facilitating the binding of substrates^8–13^.

The conformer behaviors involved in single-bond rotation are decisively determined by *dynamic equilibrium*, where the forward and reverse transitions have equal rates. The dynamics of equilibration occurring between multiple conformers are settled by electronic, vibrational, and rotational eigenstates, as well as the conformational landscape of a molecule. The simplest molecule featuring rotational isomerism along C–C bond is ethane, where the rotation occurs between two conformers: *staggered* and *eclipsed*^1,14,15^. Accordingly, ethane and its derivatives have gathered spotlights as models for studying single-bond rotation in optical spectroscopy^16–20^ and computational chemistry^1,14,15,21–23^. However, non-substituted ethane has drawbacks as a candidate system to resolve the structural aspects of single-bond rotation. Notable experimental techniques directly sensitive to molecular structures, such as scattering or diffraction, are not very sensitive to extremely light atoms like hydrogens. Hence, the lack of structural sensitivity for the six hydrogen atoms around the C–C bond makes it challenging to retrieve the structural snapshots of bond rotation in ethane (see the sensitivity plot in Supplementary Fig. 2^24^).

On the other hand, the two *staggered* conformers in heterogeneously substituted ethane derivatives (*anti* and *gauche*) render alternative avenues to study the C–C single-bond rotation, as they possess distinctive substituents which serve as fingerprints for monitoring single-bond rotation from structural perspectives. Early theoretical studies have shown that the rotational isomerization of *n*-butane, one of the simplest ethane derivatives, occurs with time constants ranging from 1 ps to 100 ps at room temperature under varying chemical environments^22,23^. A two-dimensional infrared vibrational echo spectroscopy study on another ethane derivative (1-fluoro-2-isocyanato-ethane) revealed a time constant of 43 ps for the C–C bond rotation^16^. Subsequently, time-resolved infrared and nuclear magnetic resonance spectroscopy studies reported time constants ranging from 6.3 ps (along C–C bond)^17^, 47 ps (along C–C• bond)^18^, and 3 ps to 27 ps (along C–N bond)^19^ under various steric environments around the rotating bond. Nevertheless, time-resolved optical spectroscopy and nuclear magnetic resonance commonly highlight either ultrafast temporal resolution or atomic-level structural sensitivity, but hardly feature both simultaneously^25,26^. Meanwhile, an accurate retrieval of structural dynamics requires a simultaneous interpretation of both realms. Accordingly, the ultrafast evolution of atomic-level molecular structures during single-bond rotation remains largely unexplored in solutions.

Time-resolved X-ray liquidography (TRXL), or time-resolved X-ray solution scattering (TRXSS), provides direct and detailed information on structural dynamics^27–30^, complementing the insights obtained from optical spectroscopy. To fully leverage this structural sensitivity, we chose a derivative with three hydrogen atoms at each carbon site of ethane replaced with one iodine and two fluorine atoms. As iodine and fluorine atoms have larger scattering form factors, the structural dynamics of single-bond rotation can be directly visualized by tracing how these substituted atoms move. The photodynamics of this molecule, 1,2-tetrafluorodiiodoethane (C_2_F_4_I_2_), start with the dissociation of one carbon–halogen bond, thereby breaking the symmetry between the two carbon atoms (one carbon becomes CF_2_I and the other becomes CF_2_•) and making the rotational isomerization along C–C• bond in C_2_F_4_I• easily resolvable.

**Fig. 1:**
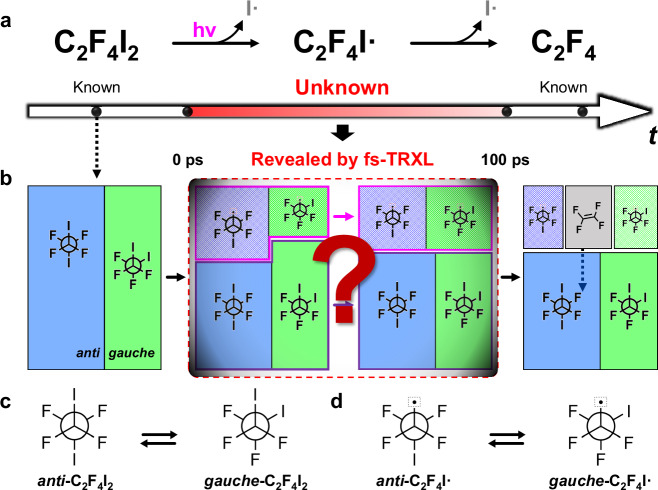
Photoinduced structural dynamics of 1,2-diiodotetrafluoroethane (C_2_F_4_I_2_). **a** Two-step photoinduced dissociation pathway of C_2_F_4_I_2_. It is known that C_2_F_4_I_2_ absorbs an ultraviolet photon at 267 nm to undergo the two-step I• dissociation to form the radical intermediate (C_2_F_4_I•) and the final product (C_2_F_4_). In cyclohexane, the secondary dissociation from C_2_F_4_I• to C_2_F_4_ is known to occur with a time constant of 292 ps, but the photodynamics before 100 ps remains unknown (emphasized with red bar). **b** The unknown ultrafast structural dynamics of C_2_F_4_I_2_, depicting possible species involved in the reaction. Before photoexcitation, the C_2_F_4_I_2_ molecules present as a mixture of *anti* (blue) and *gauche* (green) conformers. Photoinduced primary iodine dissociation depletes each conformer by different portions (purple-bordered box) depending on absorption cross-section and quantum yields. This also leaves the remaining conformational mixture C_2_F_4_I_2_ whose *anti*-to-*gauche* ratio is perturbed from the dynamic equilibrium (magenta-bordered box). **c**, **d** The rotational isomerization of (**c**) C_2_F_4_I_2_ and (**d**) C_2_F_4_I•. These perturbed *anti*-to-*gauche* ratios recover their dynamic equilibrium through the rotational isomerization of (**c**) C_2_F_4_I_2_ and (**d**) C_2_F_4_I•, while preserving the overall concentration depicted by the area of solid-bordered boxes in (**b**).

Each of C_2_F_4_I_2_ and C_2_F_4_I• has two stable conformers, *anti* and *gauche*, whose population ratio can change after excitation. In fact, a study using density functional theory (DFT) calculations suggested that *anti* and *gauche* conformers of C_2_F_4_I_2_ exhibit different absorption cross-sections. For example, in one computational study^31,32^, the relative *anti*-to-*gauche* ratio of the excitation probability to the electronic states accessible by 267 nm photon (^1^B_u_ in *anti*, ^1^B(1) in *gauche*) ranges from 80:20 to 89:11, indicating that the excitation of *anti*-C_2_F_4_I_2_ dominates over *gauche*-C_2_F_4_I_2_. These excitation ratios from C_2_F_4_I_2_ to C_2_F_4_I• are determined by the electronic properties of each C_2_F_4_I_2_ conformer. Meanwhile, the dynamic equilibrium ratios of C_2_F_4_I_2_ and C_2_F_4_I• are determined by the thermodynamic properties, implying that the excitation ratio needs not to be the same as the equilibrium ratio. These considerations suggest the possibility to trigger and capture the ultrafast single-bond rotation along C–C and C–C• bonds by observing the re-equilibration of photoinduced perturbations in *anti*-to-*gauche* ratios of C_2_F_4_I_2_ and C_2_F_4_I• (Fig. 1). Although the photodissociation of C_2_F_4_I_2_ has been extensively studied in gas^33–35^ and solution phases^36,37^, tracking the time-dependent *anti*-to-*gauche* ratios during the rotational dynamics has not been fully achieved.

In this work, we conduct a femtosecond TRXL (fs-TRXL) experiment to investigate the structural dynamics of single-bond rotation occurring within 100 ps after photoexcitation. The experimental setup for fs-TRXL is illustrated in Supplementary Fig. 1. Since the fraction of molecules participating in the rotational isomerization is far smaller than those undergoing the dissociation path, we utilize ultrabright X-ray pulses from an X-ray free-electron laser facility to capture subtle transient signals hidden beneath the dissociation dynamics. The high sensitivity of TRXL on heavy atom positions (see Supplementary Fig. 2 for the sensitivity plot^24^) enables the accurate retrieval of structural motions during rotational isomerization along single bonds (C–C and C–C•) with sub-Å precisions. Moreover, the femtosecond resolution of fs-TRXL enables the capture of sub-ps kinetics, which is particularly relevant to the rotation around C–C• bonds. By analyzing the fs-TRXL data in both reciprocal and real space, we successfully reveal the time-dependent structural dynamics, setting the stage for a detailed exploration of the single-bond rotation.

## Results and discussion

### Femtosecond TRXL of C_2_F_4_I_2_

**Fig. 2:**
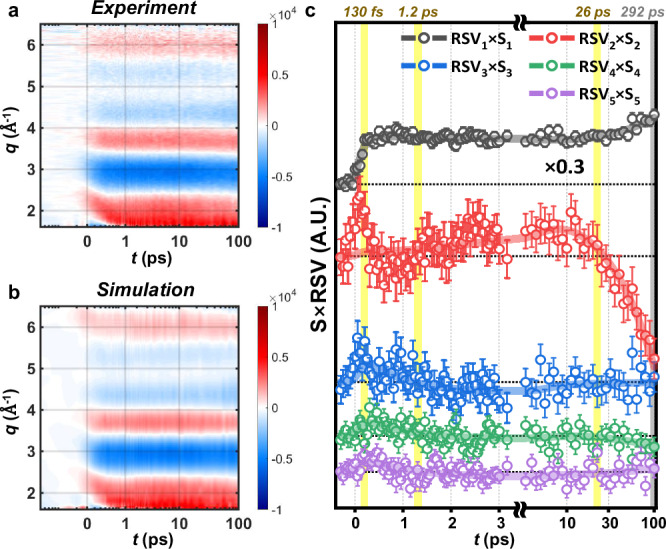
Kinetics extracted from femtosecond time-resolved X-ray liquidography (fs-TRXL) data. **a** The isotropic TRXL data (*q*ΔS_0_) of C_2_F_4_I_2_ in cyclohexane. **b** The simulated TRXL data, *q*ΔS_0_′. Isotropic TRXL data can be attributed to the molecular structural dynamics, such as primary and secondary dissociation of I• and rotational isomerization. **c** The five significant right singular vectors (RSVs, circles with vertical error bars) from the singular value decomposition (SVD) analysis of the experimental data. The error bars were estimated using the method described in Section 2-3 of the SI, where the LSVs in Supplementary Fig. 5a were used to obtain the time-dependent coefficients (S×RSV) as parameterized variables with standard errors. We conducted a global fit analysis with the sum of four exponentials, each with time constants of 130 fs, 1.2 ps, 26 ps (yellow shades), and 292 ps (grey shades to imply that the last dynamics happens slowly after the latest time delay), convoluted with a Gaussian IRF function with a width of 172 fs.

To investigate the photoinduced structural dynamics of rotation and dissociation along carbon-containing single bonds, we measured time-resolved two-dimensional X-ray scattering images of the C_2_F_4_I_2_ solution. These scattering images were decomposed into one-dimensional isotropic and anisotropic curves by utilizing the anisotropic signal decomposition method (see section 1-2 of Supplementary Information (SI) for details)^38^. The one-dimensional isotropic X-ray scattering curves (S_0_) collected after the photoexcitation were subtracted by those measured without the laser to obtain difference isotropic X-ray scattering curves. The difference isotropic X-ray scattering curves originate from the atomic pairs (1) within the solute, (2) between the solute and solvent (also known as the cage term) and (3) between the solvent molecules. Here, only the first two contributions (solute-only and cage) encrypt the structural dynamics of the solute and are termed “solute-related”. We quantitatively eliminated the contributions of (3), ΔS_0_^heat^(*q*, *t*), which originate from solvent heating (thus labeled as the solvent term), from the difference isotropic X-ray scattering curves through the projection to extract the perpendicular component (PEPC) method^39^, leaving only the solute-related data (*q*ΔS_0_(*q*, *t*), see Fig. 2a). The time-dependent changes of *q*ΔS_0_(*q*, *t*) are indicative for the solute-related structural dynamics. We constructed a comprehensive structural dynamic model, whose details are discussed throughout the following paragraphs, to disclose the origins of these features. The simulated TRXL curves (*q*ΔS_0_′(*q*, *t*), see Fig. 2b) coincide well with the experimental curves.

### Rotational isomerization along C–C and C–C• bonds

To extract the kinetic information of the solute-related dynamics, we applied singular value decomposition (SVD)^40^ on the TRXL curves. SVD decomposes the data into a product of left singular vectors (LSV(*q*)), singular values (S), and right singular vectors (RSV(*t*)). Here, LSV(*q*) contains the representative signals in *q*-space that encode the structural information of each chemical species, RSV(*t*) describes their temporal evolution, and S quantifies the relative contribution of each rank to the data (see Methods and section 1-6 of SI for details). From SVD (in Supplementary Fig. 5), we identified a total of five major components participating in the photodynamics of C_2_F_4_I_2_. To extract the solute-related kinetics, the corresponding five RSVs were globally fitted with a sum of four exponential functions, characterized by the time constants of 130 ± 50 fs, 1.2 ± 0.4 ps, 26 ± 2.4 ps, and 292 ± 136 ps, convoluted with an instrument response function (IRF) with a full-width at half maximum (FWHM) of 172 ± 47 fs representing the temporal resolution of the experiment (Fig. 2c).

To assess the dynamic origins of these time constants, we analyzed the decay-associated difference scattering curves (**DADS**(*q*)), akin to a famous concept of decay-associated spectra in time-resolved spectroscopy. Here, each DADS except for the last one (DADS_k_(*q*)) stands for the difference between scattering curves from the solution before and after the dynamics, S_before_ – S_after_, occurring with the time constant of *τ*_k_, while the last DADS accounts for the difference between initial and final states, S_final_ – S_initial_ (see section 3-2 of SI). The boldface indicates the matrix form; for instance, the k^th^ column vector of **DADS**(*q*) is DADS_k_(*q*). We constructed the matrix of normalized decays (**C**_**DADS**_**′**(*t*)) and extracted **DADS**(*q*) such that the matrix product **DADS**(*q*) × **C**_**DADS**_**′**(*t*)^T^, which equals the simulated curves (ΔS_0_′(*q*, *t*)), best agrees with ΔS_0_(*q*, *t*). We extracted DADSs for a total of four time constants (130 fs, 1.2 ps, 26 ps, and 292 ps).

**Fig. 3:**
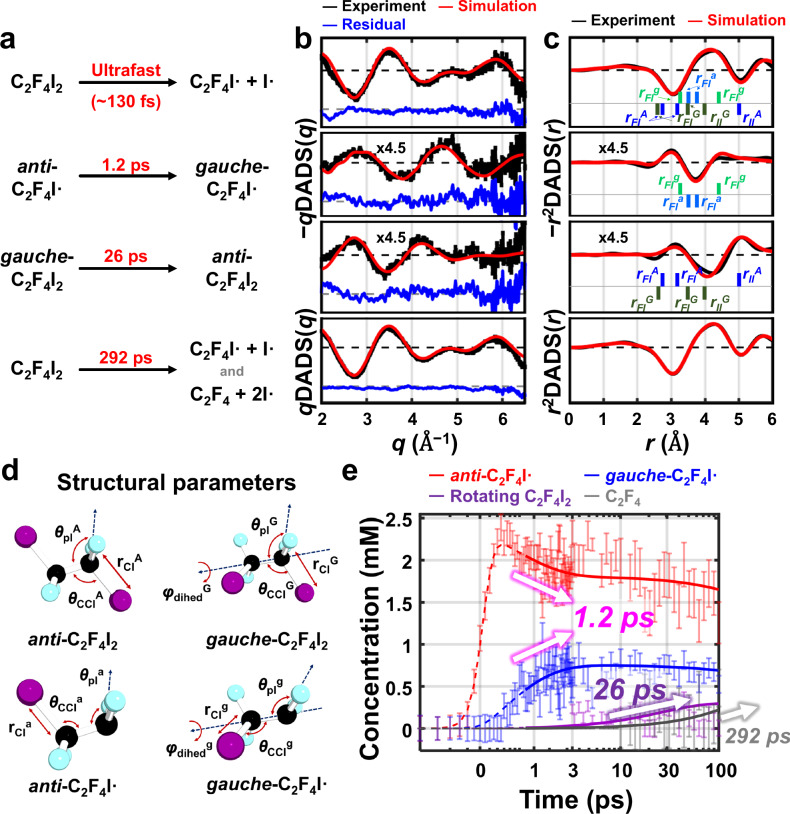
Ultrafast structural dynamics of C–C and C–C• bond rotation. **a** The reaction processes occurring in the reacting solution of C_2_F_4_I_2_ within 100 ps after photoexcitation, corresponding to the time constants of 130 fs, 1.2 ps, 26 ps, and 292 ps. **b** The experimental decay-associated difference scattering curves (*q***DADS**(*q*), black lines) corresponding to the four time constants, superimposed with respective simulated DADSs (*q***DADS′**(*q*), red lines). We modeled various difference scattering curves corresponding to the candidate transition processes that can occur upon photoexcitation of C_2_F_4_I_2_ (see Supplementary Figs. 8–10 for details) and identified that the four time constants are best attributable to the structural transitions shown in (**a**). The experimental error and fit residuals are indicated with vertical bars and blue lines, respectively. **c** The *r*-space representation of the experimental (black) and simulated (red) DADSs. In the 2^nd^ DADS, interatomic distances in the *anti*-C_2_F_4_I• (light blue lines, *r*_FI_^a^ = 3.5 Å and 3.8 Å) and *gauche*-C_2_F_4_I• (light green lines, *r*_FI_^g^ = 3.3 Å and 4.3 Å) vanish and emerge, respectively, implying the *anti*-to-*gauche* isomerization of the radical. Likewise, in the 3^rd^ DADS, interatomic distances in the *anti*-C_2_F_4_I_2_ (blue lines, *r*_II_^A^ = 5.0 Å, *r*_FI_^A^ = 2.9 Å and 3.2 Å) and *gauche*-C_2_F_4_I_2_ (green lines, *r*_II_^G^ = 4.0 Å, *r*_FI_^G^ = 2.8 Å and 3.4 Å) emerge and vanish, respectively, supporting the *gauche*-to-*anti* isomerization of C_2_F_4_I_2_. We note that only the contributions of solute-related dynamics to the scattering signal are shown in (**b**) and (**c**). **d** Structural parameters of *anti*-C_2_F_4_I_2_, *gauche*-C_2_F_4_I_2_, *anti*-C_2_F_4_I•, and *gauche*-C_2_F_4_I• used for the iterative optimization. The simulated curves in (b) and (c) were generated using these optimized structural parameters. **e** The time-dependent concentration profiles with respect to the four components: *anti*-C_2_F_4_I• (red), *gauche*-C_2_F_4_I• (blue), the *gauche*-to-*anti* rotation of C_2_F_4_I_2_ (purple), and C_2_F_4_ (gray). The error bars were estimated using the method described in section 2-3 of the SI, where the SADSs in Supplementary Fig. 12b were used to obtain the concentration profiles as parameterized variables with standard errors. We note that the third component accounts for the time-dependent concentration change of both *anti*-C_2_F_4_I_2_ and *gauche*-C_2_F_4_I_2_ simultaneously, as detailed in the methods section. In (**e**), the results of linear combination fit (LCF) and the least-χ^2^ optimization based on the kinetic modeling are shown with bars and solid lines, respectively.

To attribute the dynamic origins to each DADS_k_(*q*), we simulated difference isotropic scattering curves for various candidate structural transitions (Supplementary Fig. 6) that could take place upon the photoexcitation of C_2_F_4_I_2_. For example, DADS_2_(*q*) was compared with the simulated difference X-ray scattering curves corresponding to (i) the rotational isomerization of C_2_F_4_I_2_, (ii) the rotational isomerization of C_2_F_4_I•, (iii) the dissociation of two iodine atoms from C_2_F_4_I_2_ to form C_2_F_4_ + 2I•, and (iv) the formation of a hypothetical isomer (I_2_…C_2_F_4_, suggested in one previous study^41^) from either C_2_F_4_I_2_, C_2_F_4_I• + I•, or C_2_F_4_ + 2I•. We identified the best candidates, which have the smallest reduced chi-square (χ_ν_^2^) values, to describe the four DADSs (Fig. 3a). χ_ν_^2^ is widely utilized as a statistical criterion to evaluate the fitness of models^26,27,42^, whose details can be found in section 3-1 of SI. The comparison of each DADS with all possible pathways is demonstrated in Supplementary Figs. 8–10. Based on the statistical considerations, we identified the following candidates that best describe the DADSs: (1) the primary photodissociation of an *anti* and *gauche* mixture of C_2_F_4_I_2_ to a perturbed mixture of C_2_F_4_I• (for DADS_1_(*q*) with 130 fs), (2) the *anti*-to-*gauche* rotational isomerization of C_2_F_4_I• (for DADS_2_(*q*) with 1.2 ps), (3) the *gauche*-to-*anti* rotational isomerization of C_2_F_4_I_2_ (for DADS_3_(*q*) with 26 ps), and (4) the secondary dissociation of C_2_F_4_I• to C_2_F_4_ and I• (for DADS_4_(*q*) with 292 ps). These “correct” pathways are summarized in Fig. 3a. We confirmed the statistical significance of each dynamic pathway by analyzing the changes in discrepancies between experimental and simulated TRXL curves when each pathway was excluded the model (Supplementary Fig. 11).

The integrated analysis of DADS_k_(*q*)s with the previously assigned time constants shed light on the comprehensive structural dynamics. Firstly, upon photoexcitation, the primary I• dissociation occurs in both C_2_F_4_I_2_ conformers with the apparent time constant of 130 fs (DADS_1_(*q*)). We use the term “apparent” to emphasize that the ultrafast I• dissociation from C_2_F_4_I_2_ in fact occurs through a complicated mechanism rather than a mere exponential kinetics. This is already hinted in the discrepancy between experimental and simulated RSVs (especially in the second RSV, see section 1-5 and Supplementary Fig. 5 of SI). We described this ultrafast temporal range by considering coherent and nonadiabatic real-time atomic motions detailed in section 5 and Supplementary Fig. 14 of SI. Nevertheless, DADS_1_(*q*) can approximate the changes in scattering curve associated with the transition from the two conformers of C_2_F_4_I_2_ to the transient mixture of C_2_F_4_I_2_ and C_2_F_4_I• at which the coherent and nonadiabatic atomic motions—or the transition occurring with the time constant of 130 fs in the exponential kinetics picture—complete. In other words, while we need an in-depth analysis (in section 5 of SI) to describe *how* the intricate sub-ps dynamics occurs, the changes in the scattering curve *before and after* the ultrafast dissociation are incorporated in DADS_1_(*q*). Once the dynamics related to DADS_1_ completes, the amount of dissociated *anti* and *gauche* conformers are initially transferred intact to the *anti* and *gauche* C_2_F_4_I• mixture. However, the *anti*-to-*gauche* ratios of both remaining C_2_F_4_I_2_ and the newly-formed C_2_F_4_I• immediately after the primary photodissociation differ from those in dynamic equilibrium. Thus, the *anti* and *gauche* C_2_F_4_I• approach their dynamic equilibrium via *anti*-to-*gauche* isomerization along C–C• bond with the time constant of 1.2 ps (DADS_2_(*q*)). The non-dissociated C_2_F_4_I_2_ mixture also returns to the dynamic equilibrium via *gauche*-to-*anti* isomerization along C–C bond with the time constant of 26 ps (DADS_3_(*q*)). Finally, secondary dissociation occurs in 30% of the equilibrated C_2_F_4_I• mixture with a time constant of 292 ps, resulting in a 70:30 mixture of C_2_F_4_I• and C_2_F_4_ (DADS_4_(*q*)), consistent with the previous ps-TRXL study^37^.

**Fig. 4:**
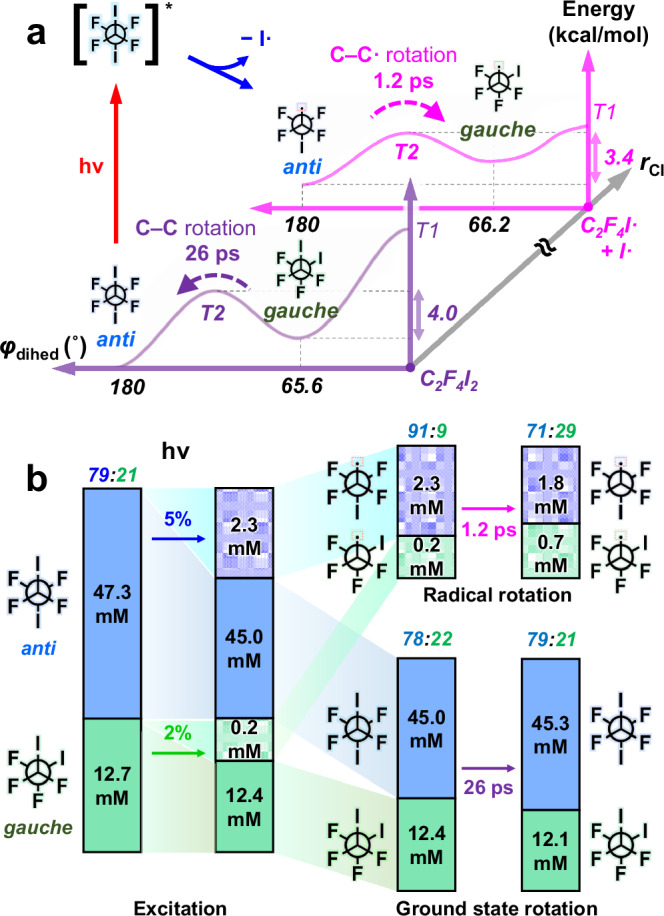
Photodynamics of C_2_F_4_I_2_ as revealed by time-resolved X-ray liquidography. **a** A schematic diagram of primary iodine dissociation and rotational isomerization on the potential energy surface (PES) with respect to *r*_CI_ and *φ*_dihed_. UV photon irradiation majorly excites *anti*-C_2_F_4_I_2_ molecules to the Franck–Condon region (red arrow). Thee excited molecules (denoted with an asterisk) rapidly dissociate into *anti*-C_2_F_4_I• and I• (blue arrow). Along the PES of C_2_F_4_I• (magenta coordinate), an excess amount of *anti*-C_2_F_4_I• molecules undergo *anti*-to-*gauche* rotational isomerization (magenta arrow), overcoming the energy barrier of 3.4 kcal/mol with a time constant of 1.2 ps. Meanwhile, along the PES of C_2_F_4_I_2_ (violet coordinate), a depleted portion of *anti*-C_2_F_4_I_2_ molecules re-equilibrate by *gauche*-to-*anti* rotational isomerization of C_2_F_4_I_2_ (violet arrow), surpassing the energy barrier of 4.0 kcal/mol with a time constant of 26 ps. We note that, at the initial step, only the *anti*-to-*anti* excitation is depicted in the diagram for simplicity. **b** Time-dependent concentrations of *anti*-C_2_F_4_I_2_, *gauche*-C_2_F_4_I_2_, *anti*-C_2_F_4_I•, and *gauche*-C_2_F_4_I•. Upon photodissociation, 5% of *anti* (blue) and 2% of *gauche* (green) C_2_F_4_I_2_ convert to their respective conformers of C_2_F_4_I•, perturbing the *anti*-to-*gauche* ratios of both C_2_F_4_I_2_ (to 45.0 mM:12.4 mM = 78:22) and C_2_F_4_I• (to 2.3 mM:0.2 mM = 91:9). Subsequently, 0.5 mM of *anti*-C_2_F_4_I• converts to *gauche*-C_2_F_4_I• with a time constant of 1.2 ps (upper-right panel), converging to a dynamic equilibrium anti-to-gauche ratio of C_2_F_4_I• (to 1.8 mM:0.7 mM = 71:29). Likewise, 0.3 mM of *gauche*-C_2_F_4_I_2_ isomerizes to *anti*-C_2_F_4_I• with a time constant of 26 ps (lower-right panel), recovering a dynamic equilibrium (to 45.3 mM:12.1 mM = 79:21). Finally, 30% of the equilibrated C_2_F_4_I• undergoes secondary dissociation into the product (C_2_F_4_) with a time constant of 292 ps, while the remaining C_2_F_4_I• persist up to 100 ps (as can be seen in Fig. 3a–c).

In general, the observed rotational isomerization time constants of 1.2 ps and 26 ps are comparable to those reported in several previous studies, which range from a few picoseconds to tens of picoseconds. However, we note that the time constant of 1.2 ps for the C–C• bond is drastically faster than the time constant of 26 ps for the C–C bond. This discrepancy may arise from different distributions of vibrational and rotational states contributing distinctively to the pre-exponential factor, and the energetically hot nature of C_2_F_4_I• (see section 7 of SI for details). To theoretically validate the observed kinetics, we applied Rice-Ramsperger-Kassel-Marcus (RRKM) theory^43–45^ to estimate the time constants for the observed rotational motions. To do this, we first calculated the shape of the potential energy surface (PES) with respect to the dihedral angle (*φ*_dihed_) for C_2_F_4_I_2_ and C_2_F_4_I• and identified two transition states (T1 and T2) along *φ*_dihed_ (Fig. 4a and Supplementary Fig. 16). Here, it becomes evident that the rotational isomerization of both C_2_F_4_I_2_ and C_2_F_4_I• proceeds through the T2 state (located between *anti* and *gauche*). To quantitatively assess the time constants, we computed the molecular geometry, free energies of ground and excited states, vibrational frequencies, and rotational moments of inertia for the three conformers (*anti*, *gauche*, and T2) of C_2_F_4_I_2_ and C_2_F_4_I•. Incorporating these data into RRKM theory provided the time constants of 20.2 ps for the *gauche*-to-T2-to-*anti* rotation of C_2_F_4_I_2_ and 1.15 ps for the *anti*-to-T2-to-*gauche* rotation of C_2_F_4_I•. These estimated values show close accordance with the experimental time constants of 26 ps and 1.2 ps (see Table 1).

**Table 1:** Experimental and theoretical kinetic constants assigned to structural dynamics

	*τ*_1_ (fs)	*τ*_2_ (ps)	*τ*_3_ (ps)	*τ*_4_ (ps)
Experiment	130 ± 50	1.2 ± 0.4	26 ± 2.4	292 ± 136
Theory	40^a^	1.15^b^	20.2^b^	
Dynamics	primary iodine dissociation	*anti*-to-*gauche *rotation of C_2_F_4_I•	*gauche*-to-*anti *rotation of C_2_F_4_I_2_	secondary iodine dissociation

^a^The time constants were referred to from the previous study which reported the primary iodine dissociation trajectory of gas-phase C_2_F_4_I_2_ using the surface hopping simulation^33^.

^b^The time constants were computed through the Rice-Ramsperger-Kassel-Marcus (RRKM) theory, as detailed in the main text.

### Structural dynamics of rotational isomerization

To fully leverage the high sensitivity of TRXL on molecular structures, based on the sensitivity plot^24^ (see Supplementary Fig. 2), we iteratively optimized four selected structural parameters—the C–I distance (*r*_CI_), the CCI angle (*θ*_CCI_), the planar angle between C–I axis and FCF plane (*θ*_pl_), and *φ*_dihed_—describing the molecular geometry of *anti*-C_2_F_4_I_2_, *gauche*-C_2_F_4_I_2_, *anti*-C_2_F_4_I• and *gauche*-C_2_F_4_I•. Moreover, we introduced four concentration-related parameters to account for population dynamics of excitation and equilibration. Using these structural and concentration-related parameters, the simulated DADSs (**DADS′**(*q*)) were computed and superimposed with **DADS**(*q*) (Fig. 3b). This rigorous analysis allows retrieving structural (Table 2) and concentration-related (Table 3) parameters for each reacting species. The signs of the first three DADSs were reversed so that the negative and positive signs match with the “depletion” and “formation” of the species. The methodological details are described in the “Structure refinement” section of Methods and section 3 of SI.

**Table 2:** Optimized structural parameters of the *anti* and *gauche* conformers of the two reacting species, C_2_F_4_I_2_ and C_2_F_4_I•

	*anti*-C_2_F_4_I_2_			*gauche*-C_2_F_4_I_2_			
	*r*_CI_^A^ (Å)	*θ*_CCI_^A^ (˚)	*θ*_pl_^A^ (˚)	*r*_CI_^G^ (Å)	*θ*_CCI_^G^ (˚)	*θ*_pl_^G^ (˚)	*φ*_dihed_^G^ (˚)
Theory^a^	2.20	115	121	2.20	116	120	66
ps-TRXL^b^	2.15 ± 0.03	112 ± 9.8	124^c^	2.14 ± 0.05	116 ± 10.4	121^c^	66^c^
fs-TRXL	2.12 ± 0.007	110 ± 0.66	120 ± 0.14	2.10 ± 0.034	114 ± 0.26	122 ± 0.25	66 ± 0.97

	*anti*-C_2_F_4_I•			*gauche*-C_2_F_4_I•			
	*r*_CI_^a^ (Å)	*θ*_CCI_^a^ (˚)	*θ*_pl_^a^ (˚)	*r*_CI_^g^ (Å)	*θ*_CCI_^g^ (˚)	*θ*_pl_^g^ (˚)	*φ*_dihed_^g^ (˚)
Theory^a^	2.22	115	137	2.20	113	137	66
ps-TRXL^b^	2.16 ± 0.04	119 ± 8.2	140^c^	2.14 ± 0.05	118 ± 9.4	138^c^	66^c^
fs-TRXL	2.16 ± 0.013	119 ± 1.3	142 ± 0.70	2.14 ± 0.011	118 ± 9.0	139 ± 3.8	67 ± 1.5

^a^The structural parameters were obtained through the geometry optimization process using extended multi-state complete active space second-order perturbation theory (XMS-CASPT2)^49^ calculations.

^b^The structural parameters were extracted from global fitting analysis of the ps-TRXL data about the photodynamics of C_2_F_4_I_2_ in cyclohexane after 100 ps^37^.

^c^These variables in the previous study were not used as fitting parameters but were instead fixed to the values obtained from the DFT calculations.

Structural parameters obtained from the femtosecond TRXL study in this work (denoted as “fs-TRXL”) are compared with those derived from computations (denoted as “Theory”) and the previous picosecond TRXL study (denoted as “ps-TRXL”)^37^. The parameters investigated include the C–I distance (*r*_CI_), the CCI angle (*θ*_CCI_), the plane angle between the C–I vector and FCF plane (*θ*_pl_), and the dihedral angle (*φ*_dihed_). These parameters were characterized for four species, with superscripts (A, G, a, g) used to distinguish between *anti*-C_2_F_4_I_2_, *gauche*-C_2_F_4_I_2_, *anti*-C_2_F_4_I•, and *gauche*-C_2_F_4_I•, respectively. Overall, the structural parameters determined from this fs-TRXL study closely align with the ps-TRXL results, particularly for radicals, where the deviations are observed between the DFT values and the ps-TRXL results.

**Table 3:** Concentration-related parameters of the photodissociation and rotational isomerization dynamics

	*f*_GS_	*f*_Rad_		*f*_exc_		*f*_rem_
ps-TRXL^a^	0.84 ± 0.037					
fs-TRXL	0.79 ± 0.048	0.71 ± 0.089		0.91 ± 0.11^b^		0.78 ± 0.004^b^

	*χ*_a_		*χ*_g_		*χ*_exc_	
ps-TRXL^a^	0.052 ± 0.0014^b^					
fs-TRXL	0.049 ± 0.0031		0.019 ± 0.0045		0.043 ± 0.0036^b^	

^a^The parameters were extracted from global fitting analysis of the ps-TRXL data about the photodynamics of C_2_F_4_I_2_ in cyclohexane after 100 ps^37^.

^b^The error values for the dependent parameters (*f*_exc_, *f*_rem_, and *χ*_exc_) were calculated from the statistical error values for the independent parameters (*f*_GS_, *f*_Rad_, *χ*_a_, and *χ*_g_) using the error propagation formula.

The symbols in the first row represent the following: the population fraction of *anti*-C_2_F_4_I_2_ in the total amount of C_2_F_4_I_2_ at equilibrium (*f*_GS_), the population fraction of *anti*-C_2_F_4_I• in the total amount of C_2_F_4_I• at equilibrium (*f*_Rad_), the fraction of *anti*-C_2_F_4_I_2_ in the dissociating population of C_2_F_4_I_2_ (*f*_exc_), the fraction of *anti*-C_2_F_4_I_2_ in the remaining population of C_2_F_4_I_2_ after dissociation (*f*_rem_), the population fraction of *anti*-C_2_F_4_I_2_ initially dissociated to *anti*-C_2_F_4_I• (*χ*_a_), the population fraction of *gauche*-C_2_F_4_I_2_ initially dissociated to *gauche*-C_2_F_4_I• (*χ*_g_), and the total dissociation ratio from C_2_F_4_I_2_ to C_2_F_4_I• (*χ*_exc_). The results from the previous ps-TRXL study are also presented for comparison. We note that, as this ps-TRXL study assumed that *χ*_a_ and *χ*_g_ are identical, *f*_GS_, *f*_Rad_, *f*_exc_, and *f*_rem_ were all regarded to be equal in the first row.

TRXL is renowned for its ability to visualize structural changes in real space with sub-Å precision. In real (*r*) space, the formation and depletion of interatomic pair distances can be more directly visualized by positive and negative signs of the oscillating *r*-space intensities. To do this, both **DADS′**(*q*) and **DADS**(*q*) in reciprocal space were Fourier sine transformed to the real space (**DADS′**(*r*) and **DADS**(*r*), see Fig. 3c). In DADS_2_(*r*), the *anti*-to-*gauche* rotation of C_2_F_4_I• is depicted by the depletions of interatomic distances in *anti*-C_2_F_4_I• (*r*_FI_^a^ = 3.54 Å and 3.76 Å) and the formations in *gauche*-C_2_F_4_I• (*r*_FI_^g^ = 3.28 Å and 4.25 Å). Likewise, in DADS_3_(*r*), the *gauche*-to-*anti* rotation of C_2_F_4_I_2_ is expressed by the depletions of interatomic distances in *gauche*-C_2_F_4_I_2_ (*r*_II_^G^ = 4.00 Å, *r*_FI_^G^ = 2.76 Å and 3.43 Å) and the formations in *anti*-C_2_F_4_I_2_ (*r*_II_^A^ = 4.99 Å, *r*_FI_^A^ = 2.87 Å and 3.20 Å). DADS_1_(*r*) and DADS_4_(*r*) show a combination of formation and depletion, as both conformers of C_2_F_4_I_2_ and C_2_F_4_I• contribute to the primary and secondary iodine dissociation dynamics.

### Time-dependent population dynamics revealed by TRXL

To retrieve the time-dependent concentrations of each component, we conducted the linear combination fit (LCF) analysis. From the combined analysis of SVD and DADS, we found five major species (*anti*-C_2_F_4_I•, *gauche*-C_2_F_4_I•, *anti*-C_2_F_4_I_2_, *gauche*-C_2_F_4_I_2_, and C_2_F_4_) contributing to ΔS_0_(*q*, *t*). The LCF results (in Fig. 3e) highlight both *gauche*-to-*anti* rotation of C_2_F_4_I_2_ and *anti*-to-*gauche* rotation of C_2_F_4_I• that take place with the time constant of 26 ps and 1.2 ps, respectively. These concentration profiles align well with the kinetics identified from the DADS analysis, validating our model and optimized structures.

The optimized concentration-related parameters (Table 3) and the LCF results (Fig. 3e) combined with the theoretical considerations provide a comprehensive picture of single-bond rotation dynamics. Initially, in the 60 mM C_2_F_4_I_2_ solution, both conformers comprise the dynamic equilibrium with the *anti*-to-*gauche* ratio of 79( ± 4.8):21. The 267 nm photon activates the antibonding orbitals along the C–I bond in both conformers, exciting the molecule to the Franck-Condon state. Within ultrafast time, the intersystem crossing occurs to the dissociative states (see Supplementary Fig. 15), where one C–I bond ultimately dissociates with the apparent time constant of 130 fs. The total amount of excited molecules is 6.8 mM, where 4.3 mM relaxes back to the ground state and 2.5 mM follows the dissociative path (see section 6-4 and Supplementary Fig. 17 of SI). When the C–I bond completely dissociates, 5% of *anti*-C_2_F_4_I_2_ and 2% of *gauche*-C_2_F_4_I_2_ convert to their corresponding C_2_F_4_I• conformers. At this point, the C_2_F_4_I• mixture contains 2.3 mM of *anti*-C_2_F_4_I• and 0.2 mM of *gauche*-C_2_F_4_I•, with an *anti*-to-*gauche* ratio of 91( ± 11):9. Soon, the C_2_F_4_I• mixture reaches dynamic equilibrium with a time constant of 1.2 ps, altering the radical composition to 1.8 mM of *anti*-C_2_F_4_I• and 0.7 mM of *gauche*-C_2_F_4_I•, leading to an equilibrium *anti*-to-*gauche* ratio of 71( ± 8.9):29. Meanwhile, the unbalanced depletion of C_2_F_4_I_2_ leaves 45.0 mM of *anti*-C_2_F_4_I_2_ and 12.4 mM of *gauche*-C_2_F_4_I_2_, modifying the *anti*-to-*gauche* ratio of C_2_F_4_I_2_ to 78( ± 3.6):22. This perturbed mixture returns to dynamic equilibrium with a time constant of 26 ps, as captured by the conversion of 0.3 mM of *gauche*-C_2_F_4_I_2_ to *anti*-C_2_F_4_I_2_. The relatively larger *gauche* fraction of C_2_F_4_I• compared to C_2_F_4_I_2_ at dynamic equilibrium implies that the free energy difference between the two conformers is smaller in C_2_F_4_I• than in C_2_F_4_I_2_ (see Supplementary Fig. 16). Finally, 30% of the equilibrated C_2_F_4_I• undergoes secondary dissociation to form C_2_F_4_ with a time constant of 292 ps, consistent with the previous ps-TRXL results. A comprehensive overview of population dynamics is presented in Fig. 4.

### Implications of reconstructing rotational isomerization from structural perspectives

In this work, we designed an experiment using an ultrashort laser pulse to transiently generate chemical environments far from equilibrium. We applied fs-TRXL on an illustrative model system of C_2_F_4_I_2_ to elucidate the C–C and C–C• single-bond rotation dynamics coupled with rotational isomerization between multiple conformers. Using a UV pump laser pulse, the non-equilibrium states of C_2_F_4_I_2_ and C_2_F_4_I• were prepared to allow several unique observations on their journey back to dynamic equilibria. For example, the rotational isomerization among the *non-photoreacted* molecules could be captured in this work by perturbing the mixture of C_2_F_4_I_2_, in sharp contrast to the typical pump-probe experiments where only the dynamics starting from the *photoexcited* molecules could be investigated. Moreover, the non-equilibrium mixture of C_2_F_4_I• features the single-bond rotation along the C–C• bond with the exceptionally faster time constant (1.2 ps) than typical values in single-bond rotation kinetics. Our analysis involved a systematic extraction of major components from the TRXL data, followed by a numerical comparison with various simulated models to account for the structural origins of the dissociation and rotational dynamics. We identified how the population and structure of the conformers, as well as their relative fractions governing each reaction step, change over time. These experimental findings are also well supported by quantum calculations performed at various levels. As a final remark, we note that traditional equilibrium assumptions often do not hold for chemical and biological processes occurring far from equilibrium. Nevertheless, direct observations of such phenomena, particularly concerning rotational coherences, remain in their infancy. In this regard, the insights from this study will pave a way for a deeper comprehension of ultrafast dynamics related to internal bond rotation, such as the coherent motions associated with re-equilibration from states far from dynamic equilibrium.

## Methods

### Time-resolved X-ray liquidography at PAL-XFEL

We prepared a 60 mM solution of 1,2-tetrafluorodiiodoethane (C_2_F_4_I_2_; 97%, SynQuest) in cyclohexane (C_6_H_12_; Aldrich, 99.9%), and used it for a femtosecond time-resolved X-ray liquidography (fs-TRXL) measurement. We also conducted a separate fs-TRXL measurement with a 6.4 mM solution of 4-bromo-4′-(*N*,*N*-diethylamino)-azobenzene (HANCHEM, 99.9%), also referred to as Azo-Br, dissolved in cyclohexane (C_6_H_12_; Aldrich, 99.9%) to elucidate the effect of the response of bulk solvent (see section 2-3 of SI). The fs-TRXL experiments were carried out at the XSS-FXL beamline of PAL-XFEL in Korea.

In the experiment, the C_2_F_4_I_2_ solution was excited with a femtosecond laser pulse at 267 nm to initiate the photodynamics. After a selected time delay, the X-ray scattering images, which encrypt the structural dynamics of the sample, were generated by the femtosecond X-ray pulses from the XFEL. The 267 nm optical laser pulses, generated as the third-harmonics of a Ti:Sapphire laser, were focused to a 132 × 132 μm^2^ spot size (full-width-at-half-maximum, FWHM) with a fluence of 2.20 mJ/mm^2^ at the sample position. The temporal width of the optical laser pulse was 130 fs (FWHM). The X-ray pulses had a narrow bandwidth (ΔE/E ≃ 0.15%) centered at 12.7 keV and were delivered at a repetition rate of 30 Hz. These X-ray pulses were focused to a spot size of 23 × 19 μm² (FWHM) at the sample position. The temporal width of the X-ray pulse was less than 50 fs (FWHM). The optical pump and X-ray probe were spatiotemporally overlapped at the stable position of the liquid jet, with a crossing angle of 10˚. The temporal widths of optical laser and X-ray pulses, the velocity mismatch caused by the width of the capillary jet, and the timing jitter altogether decide the temporal resolution of the experiment, expressed by instrument response function (IRF). We determined the IRF value of 172 ± 47 fs from the data analysis.

To ensure a continuous supply of fresh molecules at the interacting point, the sample solution was circulated through a 100-µm-thick capillary nozzle at a linear velocity faster than 3 m/s. Also, the sample solution was refreshed every two to three hours to maintain the integrity of the sample and to exclude the effects of irreversible products and radiation damages during the experiment. The X-ray scattering images were collected on the Rayonix MX225-HS area detector located 39 mm downstream of the circulating jet position. We accurately calibrated the X-ray beam center position and sample-to-detector distance by measuring the X-ray diffraction pattern of the reference sample, lanthanum hexabromide (LaB_6_), and comparing this pattern with the known diffraction peak positions.

The TRXL images were collected at various time delays ranging from −0.3 ps to 3 ps with a linear step of 50 fs, plus an additional 31 log-scaled points per decade up to 100 ps, resulting in a total of 98 time points. To maintain the temporal stability between the laser and X-ray pulses, we employed a timing feedback tool in the PAL-XFEL. To improve the signal-to-noise ratio of the data, a number of images ( ~ 8500 images) were repeatedly collected and averaged at each time delay. A typical TRXL experimental setup is depicted in Supplementary Fig. 1. Further details on the TRXL experiment can be found in previous publications^26–30,37,46^.

### Reducing the data to ΔS_0_(*q*, *t*)

The real space vectors, referenced at the X-ray beam center on the detector plane, were converted into reciprocal space vectors, whose magnitudes are defined by *q* = (4π/*λ*)sin(*θ*), where *λ* and 2*θ* denote the X-ray wavelength and scattering angle, respectively. We performed a series of corrections on the measured scattering images to address four crucial factors: (1) the solid angle of each detector pixel, (2) the polarization direction of the X-rays, (3) the alignment of the detector plane, and (4) attenuation arising from the phosphor screen of the detector. We used pyFAI^47^, a well-known python library for X-ray scattering and diffraction image processing, to perform these corrections. These corrected images were converted into the static 1D isotropic scattering curves (S_0_) by removing the anisotropic component (S_2_). Afterwards, we normalized the static S_0_ curves by dividing them with the sum of scattering intensities within the *q* range of 3–6.5 Å^−1^. These normalized S_0_ curves were further scaled to properly represent the absolute X-ray scattering signal from one solute molecule. The S_0_ curves taken without the laser (S_0_(*q*, off)) were subtracted from those taken at a time delay *t* (S_0_(*q*, *t*)) to generate the difference X-ray scattering data (ΔS_0_^raw^(*q*, *t*)) encrypting the structural dynamics information. Details of the initial data processing scheme are provided in section 1 of SI.

### Determining the number of intermediates and kinetic constants

We decomposed ΔS_0_^raw^(*q*, *t*) into the solute-related components, ΔS_0_(*q*, *t*), and the heat-related components, ΔS_0_^heat^(*q*, *t*), by implementing the projection to extract the perpendicular component (PEPC) method. The solvent heating and artifact signals are represented with **H**(*q*), a matrix obtained from the TRXL data of Azo-Br. The retrieval of **H**(*q*) and the assessment of the temperature rise of the solution are detailed in section 1-3 of SI. ΔS_0_^raw^(*q*, *t*) and ΔS_0_^heat^(*q*, *t*) are demonstrated in Supplementary Figs. 3–4.

To determine the number of intermediates and kinetic constants, we employed singular value decomposition (SVD), an algebraic technique that decomposes the data into a product of left singular vectors (LSV(*q*), time-independent components), right singular vectors (RSV(*t*), temporal evolutions) singular values (S, relative contribution of each rank). The SVD analysis of ΔS_0_(*q*, *t*) revealed five components contributing to ΔS_0_(*q*, *t*) (Supplementary Fig. 5). These five RSVs were globally fitted by a sum of multiexponential functions convoluted by a Gaussian-shaped IRF with w = 172 ± 47 fs. Satisfactory fit results were obtained using four Gaussian-convoluted exponential functions, each characterized by the shared time constants of 130 ± 50 fs, 1.2 ± 0.39 ps, 26 ± 2.4 ps, and 292 ps. The discrepancy between experimental and fitted curves in the sub-ps time domain of the second RSV can be improved by replacing 130 ± 50 fs with two exponential constants, 105 ± 13 fs and 114 ± 13 fs. Nevertheless, we used four exponentials for the kinetic analysis, as rationalized in the main text (see section 1-5 of SI for details).

### Analysis of decay-associated difference scattering curves (DADSs)

To analyze the detailed structural origins of the extracted time constants, we retrieved the experimental DADSs (denoted as **DADS**(*q*)) from ΔS_0_(*q*, *t*) using the following equation.

1 $$\Delta {{{{\rm{S}}}}}_{0}\left(q,t\right)={{{\bf{DADS}}}}\left(q\right){{{\times }}}{{{{{\bf{C}}}}}_{{{{\bf{DADS}}}}}^{\prime}\left(t\right)}^{{{{\rm{T}}}}}={\sum }_{{{{\rm{k}}}}=1}^{4}{{{{\rm{DADS}}}}}_{{{{\rm{k}}}}}\left(q\right)\times {{{{\rm{C}}}}}_{{{{{\rm{DADS}}}}}_{{{{\rm{k}}}}}}^{{\prime} }{\left(t\right)}^{{{{\rm{T}}}}}$$

Here, C_DADSk_′(*t*) is a hypothetical kinetic profile corresponding to the process associated with DADS_k_(*q*). The superscript T indicates the transpose of a matrix. Using Eq. 1, we retrieved **DADS**(*q*) that minimizes the χ^2^-distance between ΔS_0_(*q*, *t*) and **DADS**(*q*) × **C**_**DADS**_(*t*)^T^. The details are provided in section 3 of SI. We extracted and analyzed DADS_k_(*q*)s for a total of four time constants (130 fs, 1.2 ps, 26 ps, and 292 ps). C_DADSk_′(*t*) arises from zero to unity within IRF and decaying again to zero with a time constant of *τ*_k_. It is expressed by the convolution of an exponential decay with a normalized Gaussian function centered at zero, ${{{\mathscr{N}}}}$(*w*, 0; *t*), as in the following equations.

2 $${{{{\rm{C}}}}}_{{{{{\rm{DADS}}}}}_{{{{\rm{k}}}}}}^{{\prime} }\left(t\right)={{{\mathscr{N}}}}\left(w,0{;t}\right)\otimes \left(\exp \left(-\frac{t}{{\tau }_{{{{\rm{k}}}}}}\right)\right)$$

3 $${{{\mathscr{N}}}}\left(w,0{;t}\right)=\frac{1}{\sqrt{2\pi }w}\exp \left(-\frac{{t}^{2}}{2{w}^{2}}\right)$$

Here, ⊗ indicates convolution, and an IRF of *w* = 172 fs was used to construct ${{{\mathscr{N}}}}$(*w*, 0; *t*).

After retrieving **DADS**(*q*), we simulated decay-associated difference scattering curves, **DADS′**(*q*), corresponding to each potential candidate pathway in Supplementary Fig. 6. The resulting set of candidate **DADS′**(*q*) were compared with each DADS_k_(*q*). By identifying the pathway—a pair of chemical species for initial and final states—whose difference scattering curve best matches with each DADS_k_(*q*), we can attribute the dynamic pathway to each kinetic constant (*τ*_k_). We also conducted the structure refinement when constructing each DADS_k_′(*q*) as detailed in the next section. We examined the candidate pathways in Supplementary Fig. 6 for DADS_1_(*q*) (Supplementary Fig. 8), DADS_2_(*q*) (Supplementary Fig. 9), and DADS_3_(*q*) (Supplementary Fig. 10), and attributed them to the primary I• dissociation, the *anti*-to-*gauche* rotational isomerization of C_2_F_4_I•, and the *gauche*-to-*anti* rotational isomerization of C_2_F_4_I_2_, respectively. For DADS_4_, we simulated the scattering curve using the known species and concentrations at 100 ps, and subtracted the scattering curve of the photodissociated mixture of C_2_F_4_I_2_ (Fig. 3). Details of the DADS analysis is provided in section 3 of SI.

### Structure refinement

While analyzing the DADS to identify the structural transitions corresponding to each DADS, we simultaneously determined the molecular geometry of the species involved in the reaction through structure refinement. We started with the molecular geometries of the species optimized by DFT calculations and iteratively varied their structural parameters so that the simulated difference scattering curves, **DADS′**(*q*), generated from the refined structures best matched the experimental counterpart, **DADS**(*q*). The parameters, *r*_CI_, *θ*_CCI_, *θ*_pl_, and *φ*_dihed_, defined in Fig. 3d (with more details provided in Supplementary Fig. 7) were varied near the DFT-optimized values (Table 2). In C_2_F_4_I_2_, the two carbon atoms share the four structural parameters to represent symmetry. On the other hand, in C_2_F_4_I•, the structural parameters at the –CF_2_I site were taken to be equivalent to those for C_2_F_4_I_2_ of the respective conformer (denoted with the superscripts “A” or “G”), whereas those at the –CF_2_• site were defined with new parameters (denoted with the superscripts “a” or “g”).

We utilized the reduced chi-square for the k^th^ DADS, denoted as χ^2^_νk_, as a statistical criterion to decide the best-matching candidate of DADS_k_′(*q*) with DADS_k_(*q*) for k = 1–3 (see section 3-1 of SI for details of the statistical criteria). **DADS′(*q*)** were calculated using well-established formulations from scattering theory, such as the Debye equation (see section 2-1 of SI for details). The simulated **DADS′**(*q*) were converted to the *r*-space, **DADS′**(*r*), through the Fourier sine transform (in eqs. S7–S8). We adjusted the amplitude of **DADS′**(*q*) to be on the exact scale by multiplying the scattering signal from one solute molecule by the relative molar quantity corresponding to each transition. As the volume of the solution at the interaction point remains identical for every species, we simply used concentrations (c_DADSk_) to express relative molar quantities. The scale factors were determined from the kinetic model. Here, the first two equations were used to construct the scale factors, and the remaining four were introduced to build an expression for each of the four DADS_k_′(*q*)s.

4 $${{{{\rm{c}}}}}_{{{{\rm{DADS}}}}1}={{{{\rm{c}}}}}_{{{{\rm{DADS}}}}2}={{{{\rm{c}}}}}_{{{{\rm{DADS}}}}4}={{{{\rm{c}}}}}_{0}{\chi }_{{{{\rm{exc}}}}}$$

5 $${{{{\rm{c}}}}}_{{{{\rm{DADS}}}}3}={{{{\rm{c}}}}}_{0}\left(1-{\chi }_{{{{\rm{exc}}}}}\right)$$

6 $${{{{\rm{DADS}}}}}_{1}^{{\prime} }\left(q\right)={{{{\rm{c}}}}}_{{{{\rm{DADS}}}}1}\cdot \left\{\begin{array}{c}\left({f}_{{{{\rm{exc}}}}}\cdot {{{{\rm{S}}}}}_{{{{\rm{A}}}}}\left(q\right)+\left(1-{f}_{{{{\rm{exc}}}}}\right)\cdot {{{{\rm{S}}}}}_{{{{\rm{G}}}}}\left(q\right)\right)\,\hfill\\ -\left({f}_{{{{\rm{exc}}}}}\cdot \left({{{{\rm{S}}}}}_{{{{\rm{a}}}}}\left(q\right)+{{{{\rm{S}}}}}_{{{{\rm{I}}}}}\left(q\right)\right)+\left(1-{f}_{{{{\rm{exc}}}}}\right)\cdot \left({{{{\rm{S}}}}}_{{{{\rm{g}}}}}\left(q\right)+{{{{\rm{S}}}}}_{{{{\rm{I}}}}}\left(q\right)\right)\right)\end{array}\right\}$$

7 $${{{{\rm{DADS}}}}}_{2}^{{\prime} }\left(q\right)={{{{\rm{c}}}}}_{{{{\rm{DADS}}}}2}\cdot \left\{\left({f}_{{{{\rm{Rad}}}}}-{f}_{{{{\rm{exc}}}}}\right)\cdot \left({{{{\rm{S}}}}}_{{{{\rm{a}}}}}\left(q\right)-{{{{\rm{S}}}}}_{{{{\rm{g}}}}}\left(q\right)\right)\right\}$$

8 $${{{{\rm{DADS}}}}}_{3}^{{\prime} }\left(q\right)={{{{\rm{c}}}}}_{{{{\rm{DADS}}}}3}\cdot \left\{\left({f}_{{{{\rm{rem}}}}}-{f}_{{{{\rm{GS}}}}}\right)\cdot \left({{{{\rm{S}}}}}_{{{{\rm{G}}}}}\left(q\right)-{{{{\rm{S}}}}}_{{{{\rm{A}}}}}\left(q\right)\right)\right\}$$

9 $${{{{\rm{DADS}}}}}_{4}^{{\prime} }\left(q\right)={{{{\rm{c}}}}}_{{{{\rm{DADS}}}}4}\cdot \left\{\begin{array}{c}0.7\cdot \left({f}_{{{{\rm{Rad}}}}}\cdot \left({{{{\rm{S}}}}}_{{{{\rm{a}}}}}\left(q\right)+{{{{\rm{S}}}}}_{{{{\rm{I}}}}}\left(q\right)\right)+\left(1-{f}_{{{{\rm{Rad}}}}}\right)\cdot \left({{{{\rm{S}}}}}_{{{{\rm{g}}}}}\left(q\right)+{{{{\rm{S}}}}}_{{{{\rm{I}}}}\cdot }\left(q\right)\right)\right)\\+0.3\cdot \left({{{{\rm{S}}}}}_{{{{\rm{P}}}}}\left(q\right)+2{{{{\rm{S}}}}}_{{{{\rm{I}}}}}\left(q\right)\right)-\left({f}_{{{{\rm{exc}}}}}\cdot {{{{\rm{S}}}}}_{{{{\rm{A}}}}}\left(q\right)+\left(1-{f}_{{{{\rm{exc}}}}}\right)\cdot {{{{\rm{S}}}}}_{{{{\rm{G}}}}}\left(q\right)\right)\end{array}\right\}$$

Here, *f*_GS_ is the population fraction of *anti*-C_2_F_4_I_2_ in the total amount of C_2_F_4_I_2_ at equilibrium, *f*_Rad_ is the population fraction of *anti*-C_2_F_4_I• in the total amount of C_2_F_4_I• at equilibrium, *χ*_a_ is the population fraction of *anti*-C_2_F_4_I_2_ initially dissociated to *anti*-C_2_F_4_I•, *χ*_g_ is the population fraction of *gauche*-C_2_F_4_I_2_ initially dissociated to *gauche*-C_2_F_4_I•, *χ*_exc_ is the total dissociation ratio from C_2_F_4_I_2_ to C_2_F_4_I•, *f*_exc_ is the fraction of *anti*-C_2_F_4_I_2_ in the dissociating population of C_2_F_4_I_2_, and *f*_rem_ is the fraction of *anti*-C_2_F_4_I_2_ in the remaining population of C_2_F_4_I_2_ after dissociation. c_0_ indicates the initial C_2_F_4_I_2_ concentration ( = 60 mM). S_subscript_(*q*) represents the simulated X-ray scattering curve from the species denoted with the subscript, where the subscripts (A, G, a, g, P, and I•) stand for the abbreviation of *anti*-C_2_F_4_I_2_, *gauche*-C_2_F_4_I_2_, *anti*-C_2_F_4_I•, *gauche*-C_2_F_4_I•, C_2_F_4_, and I•, respectively. Among the concentration-related parameters, the first four (*f*_GS_, *f*_Rad_, *χ*_a_, and *χ*_g_) were iteratively optimized, while the later three (*χ*_exc_, *f*_exc_, and *f*_rem_) were calculated from them using the equations in section 3-3 of SI. The method to construct simulated scattering curves from the molecular structure is described in section 2-1 of SI. The theory for the DADS analysis is summarized in section 3 of SI.

### Analysis using linear combination fit (LCF)

Using the optimal model determined from the analysis of the DADSs, we constructed the simulated species-associated difference scattering curves (**SADS′**(*q*)). To obtain **SADS′**(*q*), we took the following five species into consideration: (1) *anti*-C_2_F_4_I•, (2) *gauche*-C_2_F_4_I•, (3) *anti*-C_2_F_4_I_2_, (4) *gauche*-C_2_F_4_I_2_, and (5) C_2_F_4_. Then, SADS′_k_(*q*)s were calculated by subtracting the scattering curve for the initially reacting mixture of C_2_F_4_I_2_ from that corresponding to the k^th^ species.

10 $${{{{\rm{SADS}}}}}_{{{{\rm{k}}}}}^{{\prime} }\left(q\right)={{{{\rm{S}}}}}_{{{{\rm{k}}}}}\left(q\right)-\left({f}_{{{{\rm{exc}}}}}{{{{\rm{S}}}}}_{{{{\rm{A}}}}}\left(q\right)+\left(1-{f}_{{{{\rm{exc}}}}}\right){{{{\rm{S}}}}}_{{{{\rm{G}}}}}\left(q\right)\right)$$

Here, *f*_exc_ is the fraction of *anti*-C_2_F_4_I_2_ in the dissociating population of C_2_F_4_I_2_. Also, S(*q*) indicates the simulated X-ray scattering curve from the species denoted with the subscript, where the subscripts A and G mean *anti*-C_2_F_4_I_2_ and *gauche*-C_2_F_4_I_2_, respectively. Initially, the ground state ensemble consists of a mixture of *anti* and *gauche* C_2_F_4_I_2_. Upon photoexcitation, the portion of C_2_F_4_I_2_ with an *anti*-to-*gauche* ratio of *f*_exc_:(1−*f*_exc_) are depleted from the original equilibrium composition and start to follow the photoreaction pathway, as described in the subtracted term of Eq. 10. Here, it is noteworthy that the SADSs for (3) and (4) have identical shapes but opposite signs, as both components are mathematically represented by the difference between *anti* and *gauche* C_2_F_4_I_2_. Therefore, we evaluated the scattering contributions from both *anti*-C_2_F_4_I_2_ and *gauche*-C_2_F_4_I_2_ collectively and merged the two components into one (explained as the “third” species, k = 3; therefore, the SADS for C_2_F_4_ becomes the “fourth” species, k = 4).

11 $$\begin{array}{c}{{{{{\rm{SADS}}}}}^{{\prime} }}_{{{{\rm{A}}}}}\left(q\right)\times {{{{{\rm{C}}}}}_{{{{\rm{SADS}}}},{{{\rm{A}}}}}\left(t\right)}^{{{{\rm{T}}}}}+{{{{{\rm{SADS}}}}}^{{\prime} }}_{{{{\rm{G}}}}}\left(q\right)\times {{{{{\rm{C}}}}}_{{{{\rm{SADS}}}},{{{\rm{G}}}}}\left(t\right)}^{{{{\rm{T}}}}}\\=\left({{{{\rm{S}}}}}_{{{{\rm{A}}}}}\left(q\right)-\left({f}_{{{{\rm{exc}}}}}{{{{\rm{S}}}}}_{{{{\rm{A}}}}}\left(q\right)+\left(1-{f}_{{{{\rm{exc}}}}}\right){{{{\rm{S}}}}}_{{{{\rm{G}}}}}\left(q\right)\right)\right)\times {{{{{\rm{C}}}}}_{{{{\rm{A}}}}}\left(t\right)}^{{{{\rm{T}}}}}\\+\left({{{{\rm{S}}}}}_{{{{\rm{G}}}}}\left(q\right)-\left({f}_{{{{\rm{exc}}}}}{{{{\rm{S}}}}}_{{{{\rm{A}}}}}\left(q\right)+\left(1-{f}_{{{{\rm{exc}}}}}\right){{{{\rm{S}}}}}_{{{{\rm{G}}}}}\left(q\right)\right)\right)\times {{{{{\rm{C}}}}}_{{{{\rm{G}}}}}\left(t\right)}^{{{{\rm{T}}}}}\\=\left({{{{\rm{S}}}}}_{{{{\rm{A}}}}}\left(q\right)-{{{{\rm{S}}}}}_{{{{\rm{G}}}}}\left(q\right)\right)\times {\left({{{{\rm{C}}}}}_{{{{\rm{A}}}}}\left(t\right)-{f}_{{{{\rm{exc}}}}}\left({{{{\rm{C}}}}}_{{{{\rm{A}}}}}\left(t\right)+{{{{\rm{C}}}}}_{{{{\rm{G}}}}}\left(t\right)\right)\right)}^{{{{\rm{T}}}}}\\={{{{{\rm{SADS}}}}}^{{\prime} }}_{3}\left(q\right)\times {{{{{\rm{C}}}}}_{{{{\rm{SADS}}}}3}\left(t\right)}^{{{{\rm{T}}}}}\end{array}$$

Here, Eq. 11 has a similar form as Eq. 10 since it is expressed as a multiplication of the time-dependent concentration part and the time-independent *q*-space part.

Subsequently, we retrieved the LCF concentration matrix, **C**_**SADS**_(*t*) (see Fig. 3e), that minimizes the reduced χ^2^ value between the solute-related experimental data (ΔS_0_(*q*, *t*)) and the simulated data (**SADS′**(*q*) × **C**_**SADS**_(*t*)^T^). Details of the LCF analysis are assessed in section 4 of SI. Since the amplitude of the residual signal is negligible (see Supplementary Fig. 13), we concluded that the LCF model adequately explains the TRXL data and effectively summarizes the structural dynamics of C_2_F_4_I_2_. We also provide an alternative analytical approach based on SADSs in Supplementary Fig. 12, whose details are described in section 3-3 of SI.

### Ab-initio and density functional theory (DFT) calculations

Molecular geometries of all reacting species, including *anti*-C_2_F_4_I_2_, *gauche*-C_2_F_4_I_2_, *anti*-C_2_F_4_I•, *gauche*-C_2_F_4_I•, C_2_F_4_, and C_2_F_4_…I_2_ (species “X”) were optimized using DFT. The ωB97X functional^48^, well-known for its ability to optimize geometry in line with experimental observations^26,31,32,37,46^, was utilized. These DFT-optimized structures served as the initial points for the structure refinement (see Supplementary Data 1).

To explore the nature of excited states of C_2_F_4_I_2_, potential energy curves (PECs) with respect to the C–I distance were calculated for *anti* and *gauche* C_2_F_4_I_2_ in extended multi-state complete active space second-order perturbation (XMS-CASPT2) theory^49^, from the SA(9 singlets, 11 triplets)-CAS(12e, 10o)SCF wavefunctions. Diabatic PECs were obtained, with the semi-diabatization process from the calculated adiabatic PECs. For obtaining accurate energy barriers during the C–C bond rotation, Gibbs free energies were calculated for the two transition states, namely T1 and T2, for the four species involved in rotational isomerization (*anti*-C_2_F_4_I_2_, *gauche*-C_2_F_4_I_2_, *anti*-C_2_F_4_I•, and *gauche*-C_2_F_4_I•). Furthermore, PECs with respect to the dihedral angles (ICCI for C_2_F_4_I_2_ and ICCF for C_2_F_4_I•) were calculated with three different levels of theory (CCSD(T), XMS-CASPT2, and DFT (ωB97X functional)) for the four species. The results were demonstrated in Table 2. We note that (7e, 5o) was employed for the active space of C_2_F_4_I•, analogous to the active space of C_2_F_4_I_2_.

To calculate vertical excitation energies and PECs of C_2_F_4_I_2_ with respect to the C–I distance, ANO-RCC-VDZP all-electron basis set was employed. We utilized the LANL2DZ basis set for CCSD(T) and XMS-CASPT2 calculations, and the def2-TZVPP basis set for DFT calculations. Openmolcas 8.6^50^ was used for the multireference calculations, ORCA 5.0.4^51^ was used for the CCSD(T) calculations, and Gaussian 16^52^ was used for DFT calculations.

### Molecular dynamics simulation

Molecular dynamics (MD) simulations were conducted via the MOLDY^53^ program to model the interaction between each chemical species involved in the reaction and surrounding solvent molecules. Here, we employed a virtual cubic box composed of one solute molecule surrounded by 512 rigid solvent molecules. The internal structures of each molecule (*anti*-C_2_F_4_I_2_, *gauche*-C_2_F_4_I_2_, *anti*-C_2_F_4_I•, *gauche*-C_2_F_4_I•, and I•) were fixed to the optimized ones from the DFT method, as detailed in the “Density functional theory calculation” section. The partial charge distributions of each molecule were determined by natural population analysis (NPA) using the optimized structures. In the simulation, the molecular dynamics were governed by intermolecular interactions modeled with Coulombic forces and Lennard-Jones potentials. All simulations took place at an ambient temperature of 300 K and a solvent density of 0.779 g/cm³. We initiated system equilibration via coupling to a Nose-Hoover thermostat over a 20 ps duration. The simulations were conducted within the NVT ensemble, utilizing a time step of 100 as, and trajectories were traced for a duration of 1 ns. During the simulation, molecular snapshots were saved at intervals of 100 ps. From these molecular snapshots, we first extracted the pair distribution functions (PDFs) and calculated the theoretical X-ray scattering curves for the cage term, S_cage_. This process is described in section 2-1 of SI.

### Simulating the rotational isomerization dynamics

We used the Rice-Ramsperger-Kassel-Marcus (RRKM) theory^43–45^ to calculate the theoretical kinetic constants for the rotational isomerization of C_2_F_4_I_2_ and C_2_F_4_I•. We assumed that the rotational isomerization dynamics of both C_2_F_4_I_2_ and C_2_F_4_I• takes a path trespassing either of the two transition states, T1 and T2. We utilized the optimized molecular structures, vibrational frequencies, electronic energy levels, and free energies for *anti*, *gauche*, T1, and T2 states of C_2_F_4_I_2_ and C_2_F_4_I•. The dynamic equilibrium of the forward (*anti*-to-T2-to-*gauche*) and the reverse (*gauche*-to-T2-to-*anti*) pathways leads to identical difference scattering signals with opposite signs, quenching each other in the kinetic picture. Therefore, only the dynamics arising from the re-equilibration of the perturbed state to the dynamic equilibrium are observed in our experiment. The potential energy surface with respect to the dihedral angle implies that the rotational isomerization takes place through the T2 transition state. In this context, only the theoretical kinetic constants for the assigned rotational dynamics (*gauche*-to-*anti* for C_2_F_4_I_2_, *anti*-to-*gauche* for C_2_F_4_I•) passing through the corresponding T2 states were compared with experimentally extracted values in Table 3. All computations based on the RRKM theory were conducted using the *ChemRate* software^54–56^.

In the RRKM calculation, the rate constant is expressed by the following equation.

12 $$k={a}_{1}\times \exp \left(-\frac{{a}_{2}}{{RT}}\right)$$

Here, the gas constant R is 8.314 J·mol^−1^·K^−1^, the temperature T was assumed to be 300 K, and *a*_1_ and *a*_2_ were computed through the RRKM theory. From the computation results, we found that *a*_1_ = 9.48×10^9^ and *a*_2_ = −4117.71 in the *gauche*-to-T2-to-*anti* pathway of C_2_F_4_I_2_, and *a*_1_ = 4.56×10^11^ and *a*_2_ = −1613.45 in the *anti*-to-T2-to-*gauche* pathway of C_2_F_4_I•. At 300 K, these values lead to the time constants of 20.2 ps for the C_2_F_4_I_2_ rotation and 1.15 ps for the C_2_F_4_I• rotation, as discussed in the main text.

### Reporting summary

Further information on research design is available in the Nature Portfolio Reporting Summary linked to this article.

## Supplementary information

Supplementary Information

Description of Additional Supplementary Files

Supplementary Data 1

Reporting Summary

Transparent Peer Review file

## Source data

Source data

## Data Availability

Source data for the figures in the main text and Supplementary Figs. are provided with this paper. The uploaded source data includes the processed data that can reproduce the entire key findings of this study. The raw TRXL data, which is not uploaded due to capacity issues, is securely stored on the archive servers of the beamline facilities and will be made available upon requests from readers. All other data generated in this study to support the findings of this study will also be available from the authors upon request. Source data are provided with this paper.

## Code availability

The code utilized for the analysis of time-resolved X-ray liquidography data can be obtained from the corresponding author upon request.
