# Supplementary material for: Ultrafast structural dynamics of carbon–carbon single-bond rotation in transient radical species at non-equilibrium
Source: Nat Commun. 2025 Feb 25;16:1969. doi: 10.1038/s41467-025-57279-7 (PMC11861307; doi:10.1038/s41467-025-57279-7)
Supplement: Supplementary file 1 — Supplementary Information [file 41467_2025_57279_MOESM1_ESM.pdf]

Supplementary Information for

Ultrafast structural dynamics of carbon–carbon single-bond rotation in transient radical species at non-equilibrium

Seonggon Lee[1], Hosung Ki[1], Donghwan Im[1,2], Jungmin Kim[1,2], Yunbeom Lee[1], Jain Gu[1,2], Alekos Segalina[1], Jun Heo[1], Yongjun Cha[1,2], Kyung Won Lee[1,2], Doyeong Kim[1,2], Jeongho Kim[3], Rory Ma[4], Jae Hyuk Lee[4], and Hyotcherl Ihee[1,2,*]

[1]Center for Advanced Reaction Dynamics, Institute for Basic Science (IBS), Daejeon 34141, Republic of Korea

[2]Department of Chemistry and KI for the BioCentury, Korea Advanced Institute of Science and Technology (KAIST), Daejeon 34141, Republic of Korea

[3]Department of Chemistry, Inha University, 100 Inha-ro, Michuhol-gu, Incheon 22212, Republic of Korea

[4]Pohang Accelerator Laboratory, Pohang, Gyeongbuk 37673, Republic of Korea

[*] Corresponding author. Email: hyotcherl.ihee@kaist.ac.kr

Table of Contents

Supplementary Methods and Discussions

Suppl. Note 1. Processing the TRXL data ········ 3
Suppl. Note 2. Simulation of X-ray scattering curves ········ 8
Suppl. Note 3. Analysis of the decay-associated difference scattering curves (DADSs) ········ 11
Suppl. Note 4. Analysis of the species-associated difference scattering curves (SADSs) ········ 16
Suppl. Note 5. Analysis of the coherent atomic motions related to primary dissociation ········ 17
Suppl. Note 6. Theoretical investigations ········ 18
Suppl. Note 7. Discussions on the rotational isomerization dynamics ········ 20

Supplementary Figures

Supplementary Fig. 1 | Schematic design of the TRXL experimental setup ········ 23
Supplementary Fig. 2 | Sensitivity plots for the isomers of $C_2F_4I_2$, C_2F_4I•, and C_2H_6 ········ 24
Supplementary Fig. 3 | Isotropic TRXL data of $C_2F_4I_2$ in cyclohexane ········ 25
Supplementary Fig. 4 | Solvent term and temperature change in $C_2F_4I_2$ solution in cyclohexane during the photoreaction ········ 26
Supplementary Fig. 5 | Singular value decomposition (SVD) of the PEPCed isotropic data, $\Delta S_0^{\perp}(q, t)$, for $C_2F_4I_2$ in cyclohexane ········ 27
Supplementary Fig. 6 | Candidate structural transitions between possible chemical species that may arise during the dynamics of $C_2F_4I_2$ ········ 28
Supplementary Fig. 7 | Definition of structural parameters ········ 29
Supplementary Fig. 8 | Determining the correct structural transition for $DADS_1$ ········ 30
Supplementary Fig. 9 | Determining the correct structural transition for $DADS_2$ ········ 31
Supplementary Fig. 10 | Determining the correct structural transition for $DADS_3$ ········ 32
Supplementary Fig. 11 | Statistical comparison of various dynamic models ········ 33
Supplementary Fig. 12 | Ultrafast structural dynamics of carbon–carbon single bond rotation visualized with the kinetics-constrained analysis (KCA) ········ 34
Supplementary Fig. 13 | Comparison of experimental and simulated TRXL data modeled by linear combination fit (LCF) analysis ········ 35
Supplementary Fig. 14 | Ultrafast primary iodine dissociation by modeling the time-dependent evolution of distances between carbon (C) and dissociating iodine (I_{dis}) atoms ········ 36
Supplementary Fig. 15 | Diabatized potential energy curves (PECs) of *anti* and *gauche* $C_2F_4I_2$ · 37
Supplementary Fig. 16 | Potential energy curves (PECs) with respect to the dihedral angle ········ 38
Supplementary Fig. 17 | Fraction of initially excited, relaxed, and dissociated $C_2F_4I_2$ molecules 39

Supplementary Tables

Supplementary Table 1 | Calculated harmonic frequencies of $C_2F_4I_2$ and C_2F_4I• ········ 40

Supplementary References ········ 41

Supplementary Methods and Discussions

Suppl. Note 1. Processing the TRXL data

Suppl. Note 1-1. Reduction into 1D difference X-ray scattering curves

In TRXL experiments, a two-dimensional (2D) detector is used to record the 2D pattern of the X-ray scattering intensities. These 2D patterns are then reduced to one-dimensional curves, which are subsequently analyzed. In this section, we introduce the algorithm of reducing the two-dimensional scattering images (as a function of two detector plane axes in real space) to one-dimensional curves as a function of q (the momentum transfer in reciprocal space).

In scattering theory, the two-dimensional detector plane is preferably described using the axes q and φ. Here, q is the magnitude of the momentum transfer vector, given by $q = (4\pi/\lambda)\sin(\theta)$, where 2θ and λ denote the scattering angle and X-ray wavelength, respectively, and φ represents the azimuthal angle measured from the projection of the laser polarization axis on the detector plane. The scattering angle (2θ) is defined as the angle between the incident X-ray beam and the scattered X-ray beam. It is determined by the position of the measured pixel on the detector relative to the direct beam position, which is taken as the origin of the detector plane. Therefore, if we know the sample-to-detector distance and the position of the beam center on the detector plane, the scattering angle, and consequently the magnitude of the momentum transfer vector q, can be calculated. Here, to accurately calibrate the sample-to-detector distance and determine the position of the beam center, we measured the powder X-ray diffraction pattern from a reference sample, lanthanum hexaboride (LaB_6) powder, under the same experimental geometry. We then accurately determined the beam center position on the detector and the sample-to-detector distance by analyzing the measured pattern from the reference sample. Specifically, we optimized these parameters so that the powder peak positions of the measured images accurately replicated the known powder X-ray diffraction pattern in q-space. Afterward, we performed a series of corrections on the measured scattering images to address four crucial factors: (1) the solid angle of each detector pixel, (2) the polarization direction of the X-rays, (3) the orientation of the detector plane, and (4) attenuation arising from the phosphor screen of the detector. We used pyFAI[1], a well-known python library for x-ray scattering and diffraction image processing, to perform these corrections. Then, these corrected 2D X-ray scattering images were reduced to either azimuthal curves (S_{azim}) through azimuthal averaging, or to isotropic (S_0) and anisotropic (S_2) curves through the anisotropic signal decomposition method described in the next section. The static scattering curves at positive and negative time delays were subjected to a pairing algorithm to obtain the differences before

and after photoexcitation. The raw difference scattering curves for azimuthal and isotropic data are visualized in Supplementary Fig. 3.

The time-resolved isotropic difference scattering curves (ΔS_0^{raw}), which are of interest in our posterior analysis, underwent the projection to extract the perpendicular component (PEPC) method[2] to obtain the solute-related ΔS_0 (Supplementary Fig. 3d) and heat-related ΔS_0^{heat} (Supplementary Fig. 4b). The PEPC method aims to remove the influence of solvent-related kinetics from the data so that we can extract the kinetics solely from the solute-related dynamics. The details of the PEPC method are discussed further in related papers[2,3]. We note that, in all subfigures of Supplementary Fig. 3, the curves were multiplied by q ($q\Delta S_{azim}$, $q\Delta S_0^{raw}$, $q\Delta S_0^{\perp}$, and $q\Delta S_0$) to better depict the high-q signal that majorly reflects the solute dynamics.

Suppl. Note 1-2. Extraction of isotropic signals

When a solution system is subjected to photoirradiation, both solute and solvent molecules react depending on the laser polarization. Here, a linearly polarized laser can either (1) align the solute and solvent molecules, or (2) selectively photo-excite the solute molecules depending on their relative orientation to the laser polarization. Upon photoirradiation, both effects cause an anisotropic distribution in the orientation of molecules in solution, leading to anisotropic X-ray scattering images. Therefore, using a simple azimuthal averaging of the anisotropic X-ray scattering images to yield S_{azim}, commonly used in data analysis for TRXL experiments with third-generation synchrotrons, could potentially lead to deviations from the shape theoretically modeled by the Debye equation, which assumes an isotropic molecular distribution. To address this, we utilized a well-established method[4] to extract two different components, S_0 and S_2 curves, from the scattering images. Among the two components, the isotropic scattering data (S_0) directly reflects the structural dynamics of the system. This method is based on the following equation.

$$\Delta S(q, t, \theta_q) = S_0(q, t) + P_2(\cos\theta_q)S_2(q, t) \quad \text{(S1)}$$

Here, for a scattering image measured at a time delay t, $S(q, t, \theta_q)$ is the intensity of a raw image at the pixel defined by q and θ_q, $S_0(q, t)$ and $S_2(q, t)$ stand for the isotropic and anisotropic scattering curves, respectively, and P_2 indicates the second-order Legendre polynomial. Additionally, θ_q accounts for the relative configuration comprising three angles: (1) the scattering angle between incoming and outgoing momentum transfer vectors (2θ), (2) the angle measured from the projection of the laser polarization axis on the detector plane (φ), and (3) the angle between the X-ray propagation direction and the laser

polarization axis (δ). In our experimental setup, the relationship among these angles can be simplified into the following equation, because the X-ray propagates perpendicularly to the axis of laser polarization, that is, $\delta = 90°$.

$$\cos\theta_q = \sin\theta\cos\delta - \cos\theta\cos\phi\sin\delta = -\cos\theta\cos\phi \qquad (S2)$$

Since both θ_q and the second Legendre polynomial of its cosine are solely determined by θ and φ, S_0 and S_2 could be extracted through a linear regression analysis of experimental $S(q, t, \theta_q)$ with respect to $P_2(\cos\theta_q)$. The resulting isotropic part of the data, $S_0(q, t)$, was used for further analysis.

Suppl. Note 1-3. Assessment of laser-induced solvent heating, solvent arrangement, and artifacts

Irradiation with an intense optical laser induces ultrafast alterations in the molecular arrangement of bulk solvent molecules. These changes in molecular arrangement can be classified into two main types. The first is the hydrodynamic response of the solvent due to the heat generated from the reaction[5]. In other words, changes in the temperature and density of the solution prompt distinct rearrangements of the bulk solvent molecules. In addition to this, the interaction with a linearly polarized laser causes the orientational alignment of molecules, resulting in an anisotropic distribution[6]. This alignment of solute and solvent molecules is the microscopic origin of the photoinduced change in the refractive index, a phenomenon collectively known as the optical Kerr effect (OKE)[7-9]. As a consequence of the hydrodynamic response and the OKE response of the solvent, the X-ray scattering signal from the solvent molecules changes before and after the photoexcitation. These changes in the scattering signals are commonly referred to as the solvent term[10]. The solvent term contributes to both ΔS_0 and ΔS_2[5,9,11].

To quantitatively remove the solvent term from the isotropic data (ΔS_0), the shape of the solvent term in q-space needs to be known. To determine this, we conducted a separate fs-TRXL measurement of the 6.4 mM 4-bromo-4′-(*N*,*N*-diethylamino)-azobenzene (Azo-Br) dye solution in cyclohexane. The TRXL data of this dye solution, represented as $\Delta S_0^H(q, t)$ in Supplementary Fig. 4a, was subjected to the singular value decomposition (SVD) analysis to identify the major components contributing to $\Delta S_0^H(q, t)$. As detailed in section 1-5 of SI, SVD decomposes data into left singular vectors (LSVs), right singular vectors (RSVs), and singular values. The LSVs of $\Delta S_0^H(q, t)$ contain the difference X-ray scattering curves related to the temperature rise of the solvent ($(\partial S/\partial T)_\rho$), optical Kerr effect (OKE) of the solvent, and experimental artifact arising from unstable experimental conditions, such as fluctuations in the thickness of the liquid jet, the intensity of the X-ray, or the X-ray photon energy.

We examined the signal-to-noise ratios and the shapes of the LSVs and RSVs to determine the major components. As a result, we selected ten major LSVs as the basis vectors to comprehensively represent the solvent terms and unwanted artifacts. The ten basis vectors were used as column vectors to form a matrix denoted as $\mathbf{H}(q)$. In the subsequent analyses, we applied the PEPC method to the fs-TRXL data of $C_2F_4I_2$ to extract the solute-related kinetics by removing the kinetic contributions of the solvent terms and unwanted artifacts (Supplementary Fig. 4b). Details of the PEPC method and the process for the retrieval of ΔS_0^{heat} are introduced in sections 3 and 4 of SI.

Finally, the time-dependent temperature rise of the $C_2F_4I_2$ solution ($\Delta T(t)$, see Supplementary Fig. 4c) was obtained using the following equation.

$$\mathbf{proj}_{\left(\frac{\partial S(q)}{\partial T}\right)_\rho} \Delta S_0^{heat}(q,t) \cong \left(\frac{\partial S(q)}{\partial T}\right)_\rho \times \Delta T(t) = \left\{\frac{(\text{1st LSV of } \Delta S_H)}{(\text{scale factor})}\right\} \times \Delta T(t) \qquad \text{(S3)}$$

Here, $\Delta T(t)$ represents the time-dependent temperature change of the solution. The notation "$\mathbf{proj}_{(\partial S/\partial T)\rho}\Delta S_0^{heat}$" is used to represent that only a component parallel to $(\partial S/\partial T)_\rho$ are retrieved from ΔS_0^{heat}; in general, $\mathbf{proj}_A B$ indicates the projection of a vector B onto a vector A. The scale factor in eq. S3 was determined by comparing the signal amplitude of $H_1(q)$ with that of $(\partial S/\partial T)_\rho$, which is theoretically estimated using molecular dynamics simulations. Afterward, we extracted the components of ΔS_0^{heat} that are parallel to this scaled $H_1(q)$ vector, and determined the value of $\Delta T(t)$. The resulting $\Delta T(t)$ values are illustrated in Supplementary Fig. 4c. We observed a temperature rise of approximately 1.0–1.5 K throughout the photodynamics of $C_2F_4I_2$ up to 100 ps. It exhibits a qualitative agreement with the reported temperature rise of 0.6–1.2 K in a previous TRXL study[12] that examined the dynamics of $C_2F_4I_2$ after 100 ps.

Suppl. Note 1-4. Projection to extract the perpendicular component (PEPC) method

The primary purpose of the PEPC method in our analysis is to quantitatively remove the contribution of the solvent term from the TRXL data so that the kinetics of the solute molecules can accurately be analyzed[2]. Here, we orthogonally decompose the TRXL data ($\Delta S_0^{raw}(q, t)$) into two components. One component lies along the direction spanned by $\mathbf{H}(q)$, where the column vectors of $\mathbf{H}(q)$ serve as a basis set representing the X-ray scattering components unrelated to the solute dynamics of interest (e.g., solvent heating, OKE, and artifact signals). The other component, $\Delta S_0^{\perp}(q, t)$, lies perpendicular to this direction and is independent of the kinetics of the solvent heating, OKE, and artifact signals. Therefore, $\Delta S_0^{\perp}(q, t)$ purely and precisely reflects the solute-related kinetics of interest.

We note that, since $\mathbf{H}(q)$ is not perpendicular to the pure solute-related components (ΔS_0), the q-space shape of $\Delta S_0^{\perp}(q, t)$ is distorted from ΔS_0. These distortions were compensated through the structure analysis of solute-related components, allowing us to precisely retrieve ΔS_0, as detailed in previous publications. Consequently, through PEPC, we accurately retrieved the solute-related components (ΔS_0) and heat-related components ($\Delta S_0^{heat}(q, t)$).

Suppl. Note 1-5. Determining the number of components and time constants

We conducted singular value decomposition (SVD) to extract the time-independent signal components and the time-dependent changes in their contributions to the signal. Through SVD, ΔS_0 is decomposed into three matrices satisfying the relationship $\Delta S_0 = \mathbf{USV}^T$. Here, $\mathbf{U}$ is a matrix with a size of $n_q \times n_t$ whose column vectors ($U_k(q)$) are left singular vectors (LSVs), $\mathbf{V}$ is a matrix with a size of $n_t \times n_t$ whose column vectors ($V_k(t)$) are right singular vectors (RSVs), and $\mathbf{S}$ is a diagonal matrix with a size of $n_t \times n_t$ whose diagonal elements (S_{kk}) are singular values. The LSVs represent the time-independent signal components in q-space, the RSVs account for the time-dependent amplitude change of the respective LSVs in t-space, and singular values are quantitative measures for the relative significance, or the contribution, of the corresponding LSVs and RSVs to the data. The autocorrelation values for LSVs and RSVs were termed $\mathbf{C}(\mathbf{U})$ and $\mathbf{C}(\mathbf{V})$, respectively, in Supplementary Fig. 5. Large values of S_{kk}, $\mathbf{C}(U_k)$, and $\mathbf{C}(V_k)$ indicate that the kth-rank LSV and RSV significantly contribute to $\Delta S_0(q, t)$. In general, when plotting the values from high rank (starting from the 1st LSV and RSV, rank 1) to low rank, the S_{kk}, $\mathbf{C}(U_k)$, and $\mathbf{C}(V_k)$ values are typically higher for high ranks and lower for low ranks. Ideally, this distribution shows a sudden drop, followed by a flat plateau corresponding to the low-rank ones. This flat plateau at lower ranks indicates that the corresponding LSVs and RSVs represent random noise (or components with negligible contributions). Considering this, LSVs and RSVs with high S_{kk}, $\mathbf{C}(U_k)$, and $\mathbf{C}(V_k)$ values before reaching the plateau contribute much more significantly to the data than the random noise. Thus, the high-rank LSVs and RSVs before the sudden drop can be considered significant components. Following this criterion, we identified five principal components in our data, as depicted in Supplementary Fig. 5a.

To extract the kinetics information from the data, we analyzed the significant RSVs. The five selected RSVs, after being weighted by their respective singular values (resulting in $S_{11}V_1$, $S_{22}V_2$, …, and $S_{55}V_5$), were globally fitted with a sum of multiple exponential functions convoluted by a Gaussian function representing the broadening effect of instrument response function (IRF). The best-fit results

were obtained using five exponential functions with the time constants of 105 ± 13 fs, 114 ± 13 fs, 1.18 ± 0.39 ps, 26.2 ± 2.4 ps, and 292 ± 136 ps, with the retrieved IRF value of 172 ± 47 fs (Supplementary Fig. 5b). These values were used to construct kinetic models in the analyses of DADSs and SADSs.

Here, we emphasize that the earliest two adjacent time constants (105 fs and 114 fs) must be handled with a special care. Since 1) they are overlapping within their error bounds, 2) the difference is smaller than the temporal interval of our experiment (50 fs), and 3) they are both smaller than the IRF, these two time constants in fact do not reflect the physics within ultrafast temporal domain. Instead, they imply the presence of coherent atomic motions, which cannot be described simply through exponential kinetics. In addition, the proximity between the two time constants causes the respective concentration profiles to be almost linearly dependent, resulting in unwanted mathematical artifacts during algebraic decomposition of the data. Thus, we implemented a step-by-step analysis strategy: (1) for the kinetic part, we simplified the intricate ultrafast dynamics with one representative time constant; and (2) for the structural dynamics part, we conducted a separate structural analysis for the TRXL data before 1.2 ps where the ultrafast time constants matter. For (1), we fitted the RSVs with four Gaussian-convoluted exponentials (Supplementary Fig. 5c) to approximate the two earliest time constants (105 ± 13 fs and 114 ± 13 fs) into one (130 ± 50 fs).

Suppl. Note 1-6. Analyzing the structural dynamics

After retrieving all the kinetic constants, we applied three different analytical methods to attribute the correct structural transitions to each time constant, construct a kinetic model, and optimize the molecular structures. The three methods are decay-associated difference scattering curve (DADS) analysis, species-associated difference scattering curve (SADS) analysis, and linear combination fit (LCF) analysis. DADS analysis aims to identify which pair of reactants and products best describe each kinetic constant. On the other hand, SADS and LCF analyses focus more on validating the kinetic model. We comprehensively discuss the three methods in sections 3 and 4 of SI.

Suppl. Note 2. Simulation of X-ray scattering curves

Suppl. Note 2-1. Generation of simulated X-ray scattering curves

The simulated X-ray scattering curves were calculated using standard X-ray scattering formulas. The theoretical difference X-ray scattering curves of the solution comprises three factors: (i) the solute term, (ii) the cage term, and (iii) the solvent term.

First, the solute term is computed directly from the form factor of atomic constituents (f_i and f_j) and the interatomic distances (r_{ij}) of each solute molecule. This computation is based on the Debye equation[13], as shown below:

$$S_{\text{solute}} = \sum_i f_i(q)^2 + \sum_i \sum_{j \neq i} f_i(q) f_j(q) \frac{\sin(q r_{ij})}{q r_{ij}} \tag{S4}$$

The cage term describes the signal component resulting from the interference between two waves, each scattered by a solute atom and a solvent atom, respectively. It is calculated using pairwise distribution functions (PDF), $g_{ij}(r)$, which represent the distribution of distances between atoms. Here, i indexes atom types, or elements, of the atoms belonging to the solute molecule, and j indexes atom types, or elements, of the atoms in the solvent molecule. These PDFs are extracted from molecular dynamics (MD) snapshots[14].

$$S_{\text{cage}} = \sum_i \sum_{j \neq i} \frac{N_i N_j}{V} f_i(q) f_j(q) \int_0^\infty \left(g_{ij}(r) - 1\right) \frac{\sin(q r_{ij})}{q r_{ij}} 4\pi r^2 dr \tag{S5}$$

Here, N_i, N_j, and V represent the number of atoms corresponding to the ith and j^{th} atom types, and the number of atoms contained in the overall volume of the virtual MD box, respectively.

Finally, the solvent term is extracted from a separate TRXL measurement using 6.4 mM of Azo-Br dye dissolved in cyclohexane. In this dye solution, photon induces structural changes primarily related to the rearrangement of solvent molecules[5,9,10]. These changes are caused by the photoinduced alignment of the solvent molecules and a temperature rise in the solution, but do not result in any significant structural change within the dye molecule. Thus, the 1D scattering curves obtained from this dye solution, ΔS_0^{heat}, can be used to remove the contributions of solvent response from the TRXL data for our main system, $C_2F_4I_2$ solution. The consideration of the solvent term is explicitly described in section 1-2 of SI.

Suppl. Note 2-2. Fourier sine transforms into real space

In general, the X-ray scattering curves in reciprocal space, S(q), can be converted into radial distribution functions (RDFs) in real space, R(r), through a Fourier sine transformation, as shown in the following equation.

$$r^2 \mathrm{R}(r, t) = \int \frac{r}{2\pi^2} q \mathrm{S}(q,\ t) \sin(qr) \exp(-q^2 \alpha)\, dq \tag{S6}$$

where $\exp(-q^2\alpha)$ is a damping term that compensates for the finite range of q space in the Fourier sine transform, and α is usually taken as $\alpha = 0.03$ Å^2. This transformation provides a more intuitive view of the structure, as the resulting $R(r_k)$ directly depicts the existence and abundance of the atomic pairwise distance at r_k[14].

The same transformation can be applied not only to $S(q, t)$ but also to various types of difference scattering curves. In this case, the formation and depletion of interatomic distances within the reacting solution are indicated by the positive and negative amplitudes of resulting difference RDFs along the r-axis. Here, not only the atomic pair distances between atoms in solute molecules, but also those between solute and solvent molecules, as well as between solvent molecules, contribute to the difference RDFs. To focus on the change in the first type of interatomic distances—in other words, the structural changes related to solute molecules—we applied the Fourier sine transform to the difference scattering curves corresponding to the solute-related structural changes. These curves include the experimental DADSs ($\mathrm{DADS_k}(q)$, eq. S7) and the theoretical DADSs ($\mathrm{DADS_k}'(q)$, eq. S8), which exclusively contain solute-related dynamics.

$$r^2\mathrm{DADS_k}(r,t) = \int \frac{r}{2\pi^2} q\mathrm{DADS_k}(q,t)\sin(qr)\exp(-q^2\alpha)\, dq \quad \text{(S7)}$$

$$r^2\mathrm{DADS}_\mathrm{k}^{\prime(r,t)} = \int \frac{r}{2\pi^2} q\mathrm{DADS}_\mathrm{k}'(q,t)\sin(qr)\exp(-q^2\alpha)\, dq \quad \text{(S8)}$$

The experimental and theoretical DADSs are shown both in reciprocal ($\mathrm{DADS}(q)$ and $\mathrm{DADS}'(q)$, in Fig. 3b) and real ($\mathrm{DADS}(r)$ and $\mathrm{DADS}'(r)$, in Fig. 3c) spaces. A detailed explanation of the DADS concept will be thoroughly discussed in section 3-2 of SI.

Suppl. Note 2-3. Estimation of standard errors

We estimated the statistical errors of the experimental data and the fitted parameters as follows: (1) estimating the standard error of the mean of the experimental TRXL curves (defined as $\sigma(q, t)$), (2) calculating how the chi-square (χ^2) value of a model varies as a function of the fitted parameters during the optimization process, (3) evaluating the Hessian matrix of χ^2 on a space spanned by the parameters, and (4) retrieving the standard error, which is the square root of two times the diagonal elements of the inverse of the Hessian matrix.

The first step aims to extract the standard error of the mean, $\sigma(q, t)$. As described in section 1-1 of SI, the experimental TRXL data used in the analysis is the average of multiple difference scattering

curves, each representing the difference between two static curves (one taken after the photoexcitation and the other taken without the laser). The standard error is calculated by taking the square root to the deviation divided by the number of images at each time delay minus one. Finally, the standard error of the mean is calculated by further dividing the standard error by the square root of the number of images.

The second step focuses on the evaluation of goodness-of-fit. In this study, we used the concept of chi-square (χ^2) and reduced chi-square (χ_v^2), which are textbook criteria to statistically evaluate how well the model fits to the data. Here, the χ^2 value is calculated as the mean-square distance divided by the square of $\sigma(q, t)$ summed over the entire (q, t) space. The χ_v^2 value is obtained by dividing χ^2 by ($n_q \times n_c - p - 1$), where n_q is the number of q-space points, n_c is the number of simultaneously fitted curves (equals to the number of time constants in the DADS or SADS analyses, and the number of time points in the global fit analysis), and p is the number of parameters. The detailed formulas utilized specifically for this study are provided in eqs. S12 and S13. The optimization process is essentially finding the set of parameters, denoted as (X_1, X_2, …, X_p), that minimizes the χ^2 value. We note that these symbolic notations for the parameters will be used only in this section (section 2-3 of SI).

The third and fourth steps focus on the evaluation of the Hessian matrix, which assesses how the goodness-of-fit becomes worse if the set of optimized parameters slightly deviates from their global minimum. The Hessian matrix H has a size of p by p whose ij^{th} elements defined as $H_{ij} = \partial\chi^2/\partial(X_iX_j)$. If we apply the Taylor expansion to χ^2 near the global minimum set of (X_1, X_2, …, X_p), this Hessian matrix divided by 2! will be the coefficient for the square of parameter deviation (ΔX_1, ΔX_2, …, ΔX_p). Accordingly, the standard error of the kth parameter is the square root of two times the corresponding diagonal term of the inverse Hessian matrix, $([2 \times H^{-1}]_{kk})^{½}$. This value is reported as the error of each parameter in Tables 1–3.

We note that this estimation addresses random noise only and does not account for the potential presence of systematic errors or artifacts in the data. If such systematic errors or artifacts contribute to our data, the actual error values of the parameters may differ from the calculated values. In this regard, the seemingly small errors can be interpreted as precision rather than accuracy. However, as we found no evidence of systematic errors or artifacts in our data, we have not made additional considerations regarding potential distortions in error values caused by such factors.

Suppl. Note 3. Analysis of the decay-associated difference scattering curves (DADSs)

Suppl. Note 3-1. General concept

The concept behind the DADS analysis is similar to that of decay-associated spectra (DAS), a well-known method in time-resolved optical spectroscopy used to attribute the origin of the observed signal changes corresponding to each time constant. This analysis is performed after all the time constants are determined from the kinetic analysis of the time-resolved data. Here, we assume that the number of time constants present is n_τ. To extract DADS, a matrix of hypothetical normalized decay kinetic profiles ($\mathbf{C_{DADS}}'(t)$) is generated. The kth column vector of $\mathbf{C_{DADS}}'(t)$, denoted as $\mathbf{C_{DADSk}}'(t)$, has elements that increase from zero to unity and then decay back to zero with a time constant of τ_k, convoluted by a normalized Gaussian function centered at time zero with the width w representing the IRF. The matrix of experimental decay-associated difference scattering curves (DADS(q)) are retrieved from the solute-related data (ΔS0(q, t)) so that eqs. S9–11, shown below, hold. Here, we note that one slowly decaying profile (with $\tau_{n\tau+1}$ is far slower than the longest time delay measured in this experiment) must be added after the last one to represent the long-lived species that did not completely decay within the time window covered in this experiment.

$$\mathrm{C}'^{(t)}_{\mathrm{DADSk}} = \mathcal{N}(w,0;t) \otimes \left(\exp\left(-\frac{t}{\tau_\mathrm{k}}\right)\right) \tag{S9}$$

$$\mathcal{N}(w,0;t) = \frac{1}{\sqrt{2\pi}w}\exp\left(-\frac{t^2}{2w^2}\right) \tag{S10}$$

$$\Delta \mathrm{S}_0(q,t) = \mathbf{DADS}(q) \times \mathbf{C'_{DADS}}(t)^\mathrm{T} = \sum_{\mathrm{k}=1}^{4} \mathrm{DADS_k}(q) \times \mathrm{C'_{DADSk}}(t)^\mathrm{T} \tag{S11}$$

Next, we extracted the structural information of each species from the experimental DADSs. To achieve this, we modeled simulated X-ray difference scattering curves ($\mathbf{DADS'}(q)$) corresponding to the candidate dynamic processes that could occur with the time constant and compared them with each column of the experimental DADSs ($\mathbf{DADS}(q)$). By varying the molecular structural parameters and simulating $\mathbf{DADS'}(q)$ based on these parameters, we performed a least-χ^2-optimization to ensure that $\mathbf{DADS'}(q)$ best matched $\mathbf{DADS}(q)$. The χ_v^2 value for the kth DADS, denoted as χ^2_{vk}, is calculated using eq. S12, and the total χ_v^2 values are expressed as shown in eq. S13.

$$\chi^2_{v\mathrm{k}} = \frac{\sum_q \left|\frac{\mathrm{DADS_k}(q) - \mathrm{DADS_k}'(q)}{\sigma_{\mathrm{DADS_k}}(q)}\right|^2}{\mathrm{n}_q - \mathrm{n}_p - 1} \tag{S12}$$

$$\chi^2_v = \sum_\mathrm{k} \chi^2_{v\mathrm{k}} \tag{S13}$$

The standard deviation of $DADS_k(q)$, denoted as $\sigma_{DADSk}(q)$, is evaluated from the standard deviation of $\Delta S_0(q, t)$ through error propagation theory. The reduced-χ^2 value for the kth DADS, denoted as χ^2_{vk}, is calculated by dividing the sum of χ^2-values for all q-space points by $(n_q - n_p - 1)$, where n_q and n_p stand for the number of q-space points and the degrees of freedom (equals to the number of iterated parameters), respectively. Finally, the reduced-χ^2 value is computed by taking the sum of χ^2_{vk} for all DADSs and is used as a statistical criterion for the analysis.

Suppl. Note 3-2. Discussions on the DADS analysis and sign considerations

In this section, we introduce the concept of DADS analysis using a simplified example of a hypothetical sequential kinetic model ([A] → [B] → [C]). In this model, the reaction starts from the reactant [A] and ends at the product [C] through a sequential two-step process. The time constants for these two steps are τ_1 and τ_2, respectively. Following the logic in section 3-1 of SI, we can define three DADSs, denoted as $DADS_1(q)$, $DADS_2(q)$, and $DADS_3(q)$. Each DADS corresponds to the normalized decay profiles: $C_{DADS1}'(t)$, which corresponds to τ_1, and $C_{DADS2}'(t)$, which corresponds to τ_2, as well as the profile corresponding to the long-lived species, $C_{DADS3}'(t)$. At the latest measured time delay, the contributions of the first two DADSs have already been decayed to zero whereas only the last DADS, $DADS_3(q)$, survives with the concentration of $C_{DADS3}'(t \gg \tau_2) = 1$. At this moment, only species [C] remains in the solution, and the scattering signal from species [C] subtracted by that from the initial reactant species [A] is experimentally measured. Therefore,

$$\Delta S(q,\, t \gg \tau_2) = S_{[C]}(q) - S_{[A]}(q) = DADS_3(q) \tag{S14}$$

Now, at the time delay before the final transition occurs with the time constant of τ_2, the solution predominantly contains species [B]. The difference scattering curve corresponding to the solution at this time can be explained by the sum of $DADS_2(q)$ and $DADS_3(q)$ because $C_{DADS1}'(\tau_2 \ll t \ll \tau_3) = 0$ and $C_{DADS2}'(\tau_2 \ll t \ll \tau_3) = C_{DADS3}'(\tau_2 \ll t \ll \tau_3) = 1$.

$$\Delta S(q,\, \tau_1 \ll t \ll \tau_2) = S_{[B]}(q) - S_{[A]}(q) = DADS_2(q) + DADS_3(q) \tag{S15}$$

Then, we obtain the expression for $DADS_2(q)$ by substituting eq. S15 to eq. S14.

$$DADS_2(q) = S_{[B]}(q) - S_{[A]}(q) - DADS_3(q) = S_{[B]}(q) - S_{[C]}(q) \tag{S16}$$

Likewise, the first DADS will be represented in the following equation.

$$DADS_1(q) = S_{[A]}(q) - S_{[B]}(q) \tag{S17}$$

Consequently, it is possible to assign a structural transition for each time constant by finding a pair of two species (before and after the transition) where the difference scattering curve, S(before) – S(after), best fits each DADS. It is important to note that the expression for the last DADS ($DADS_3(q)$ in the example) differs slightly from the others, as it is not expressed as S(before) – S(after) but rather as the subtraction of the X-ray scattering curve for the initial reactant from that of the final product, S(product) – S(reactant). This explains why the sign of the last DADS ($DADS_4(q)$ in our TRXL data for $C_2F_4I_2$) was preserved, while the other three DADSs ($DADS_1(q)$, $DADS_2(q)$, and $DADS_3(q)$ in our TRXL data for $C_2F_4I_2$) were inverted, in Fig. 3. Attentions must be paid to the signs in each DADS to ensure that the negative and positive signs correctly match the "depletion" and "formation" of a species.

Suppl. Note 3-3. Practical application of DADS analysis to the photodynamics of $C_2F_4I_2$

Applying DADS analysis to the TRXL data of $C_2F_4I_2$ is less straightforward compared to the example introduced in section 3-2 of SI, as the photodynamics of $C_2F_4I_2$ does not follow sequential kinetics, but starts from two distinct ground state conformers, *anti* and *gauche*. However, each kinetic constant still corresponds to the transition from one species in the reacting mixture to the other. As the DADS analysis uniquely determines one experimental $DADS_k(q)$ per each τ_k, we can compare it with simulated difference scattering curves ($DADS_k'(q)$) derived from candidate structural transitions (see Supplementary Fig. 6). Among these various candidate structural transitions, the structural transition that yields $DADS_k'(q)$ matching with $DADS_k(q)$ is regarded as the correct answer for τ_k.

From the kinetic analysis, we retrieved three time constants within 100 ps: 130 fs, 1.2 ps, and 26 ps, along with one post-100 ps time constant of 292 ps. In the previous study, the last time constant has already been assigned to the secondary dissociation of C_2F_4I• to C_2F_4 and I•. To identify the structural origins of the three newfound time constants (ultrafast (130 fs), 1.2 ps, and 26 ps), we simulated $DADS_k'(q)$ corresponding to various candidate processes and compared their q-space shapes with $DADS_k(q)$ (for k = 1–3). The reduced chi-squares ($\chi^2_{\nu k}$) were used as criteria to check "goodness-of-fit" for $DADS_1(q)$, $DADS_2(q)$, and $DADS_3(q)$, as depicted in Supplementary Figs. 8–10.

At this step, we independently analyzed each DADS and each candidate structural transition; the simulated DADSs were individually scaled only to minimize the χ^2 values with the experimental DADSs. In other words, only the q-space shapes were compared between experimental and simulated DADSs. This analytical approach was inevitable at this stage since we intended to determine the correct structural transition for each time constant without any prejudice to the kinetic model. But, in practice,

the concentration of each species cannot be independent of each other (for example, for a transition from one species to the other, the depleted amount of reactant and the formed amount of product must be coincident). Thus, to accurately model the population change of each species during the photoreaction, constraints must be imposed to the concentration-related scaling constants for each DADS. In this regard, the simulated difference scattering curves were scaled accordingly by a concentration-related scale factor (c_{DADSk}) to yield $DADS_k'(q)$. To fully leverage the high structural sensitivity of TRXL, we also conducted structure refinement of the related species while simultaneously assigning the structural transition corresponding to each time constant. This was achieved by iteratively adjusting the structural parameters, starting from the DFT-optimized values, for each reacting species (see Supplementary Fig. 7).

We optimized the scaling factors (c_{DADSk} for k = 1–4) under the constraints based on the kinetic model. In a correct kinetic model in Fig. 3, the coefficients, denoted as c_{DADSk} (k = 1–4), are constrained as detailed below (eqs. S18 and S19). Under these constraints, $DADS_k'(q)$ (k = 1–4) can be expressed as follows (eqs. S20–S23).

$$c_{DADS1} = c_{DADS2} = c_{DADS4} = c_0\chi_{exc} \tag{S18}$$

$$c_{DADS3} = c_0(1-\chi_{exc}) \tag{S19}$$

$$DADS_1'(q) = c_{DADS1} \cdot \begin{Bmatrix} \left(f_{exc} \cdot S_A(q) + (1-f_{exc}) \cdot S_G(q)\right) \\ -\left(f_{exc} \cdot \left(S_a(q) + S_I(q)\right) + (1-f_{exc}) \cdot \left(S_g(q) + S_I(q)\right)\right) \end{Bmatrix} \tag{S20}$$

$$DADS_2'(q) = c_{DADS2} \cdot \left\{(f_{Rad} - f_{exc}) \cdot \left(S_a(q) - S_g(q)\right)\right\} \tag{S21}$$

$$DADS_3'(q) = c_{DADS3} \cdot \left\{(f_{rem} - f_{GS}) \cdot \left(S_G(q) - S_A(q)\right)\right\} \tag{S22}$$

$$DADS_4'(q) = c_{DADS4} \cdot \begin{Bmatrix} 0.7 \cdot \left(f_{Rad} \cdot \left(S_a(q) + S_I(q)\right) + (1-f_{Rad}) \cdot \left(S_g(q) + S_{I\cdot}(q)\right)\right) \\ +0.3 \cdot \left(S_P(q) + 2S_I(q)\right) - \left(f_{exc} \cdot S_A(q) + (1-f_{exc}) \cdot S_G(q)\right) \end{Bmatrix} \tag{S23}$$

Here, f_{GS}, f_{Rad}, χ_a, and χ_g are independent variables describing the concentration fractions of *anti*-$C_2F_4I_2$ in the total amount of $C_2F_4I_2$ at equilibrium, *anti*-C_2F_4I• in the total amount of C_2F_4I• at equilibrium, *anti*-$C_2F_4I_2$ initially dissociated to *anti*-C_2F_4I•, and *gauche*-$C_2F_4I_2$ initially dissociated to *gauche*-C_2F_4I•, respectively. These parameters determine the three dependent variables: χ_{exc}, f_{exc}, and f_{rem}, which indicate the total dissociation ratio from $C_2F_4I_2$ to C_2F_4I•, the fraction of *anti*-$C_2F_4I_2$ in the dissociating population of $C_2F_4I_2$, and the fraction of *anti*-$C_2F_4I_2$ in the remaining $C_2F_4I_2$ after initial dissociation,

respectively. c_0 indicates the initial $C_2F_4I_2$ concentration (= 60 mM). The expressions for the three dependent parameters are given in the following equations.

$$\chi_{\mathrm{exc}} = f_{\mathrm{GS}}\chi_{\mathrm{a}} + (1 - f_{\mathrm{GS}})\chi_{\mathrm{g}} \tag{S24}$$

$$f_{\mathrm{exc}} = \frac{f_{\mathrm{GS}}\chi_{\mathrm{a}}}{\chi_{\mathrm{exc}}} \tag{S25}$$

$$f_{\mathrm{rem}} = f_{\mathrm{GS}}\frac{1 - \chi_{\mathrm{a}}}{1 - \chi_{\mathrm{exc}}} \tag{S26}$$

Through the integrated DADS analysis, we identified the most consistent structural transitions for the time constants: 130 fs (primary I• dissociation; Supplementary Figs. 6b and 6c), 1.2 ps (*anti*-to-*gauche* rotation of C_2F_4I•; the forward path of Supplementary Fig. 6i), and 26.2 ps (*gauche*-to-*anti* rotation of $C_2F_4I_2$; the reverse path of Supplementary Fig. 6h). We also confirmed that $\mathrm{DADS}_4(q)$ for a time constant of 292 ps agrees well with the known secondary iodine dissociation, modeled via a linear combination of the structural transitions described in Supplementary Figs. 6b, 6c, 6j, and 6k (comparisons shown in the 4th row of Fig. 3b). The optimal values of the structural parameters and the concentration-related parameters are summarized in Table 2 and Table 3, respectively. The error value for each parameter is calculated from the standard deviation of $\mathbf{DADS}(q)$, denoted as $\boldsymbol{\sigma}_{\mathbf{DADS}}(q)$. The error propagation from the experimental standard deviation of $\Delta S_0(q, t)$ to $\boldsymbol{\sigma}_{\mathbf{DADS}}(q)$ was computed by inverting the Hessian matrix and taking the square root, using the *fminunc* library in MATLAB. The error of the optimized parameters was calculated from $\boldsymbol{\sigma}_{\mathbf{DADS}}(q)$ using the HESSE function of the *fminuit* library in MATLAB, provided by CERN.

Suppl. Note 4. Analysis of the species-associated difference scattering curves (SADS)

While the DADS analysis focuses on the structural transition associated with each *time constant*, the other two analyses (SADS analysis and LCF) focus on the *species* involved in the reaction. The latter two methods (SADS analysis and LCF) have commonly been used in the analysis of time-resolved data as they provide a more intuitive and easily understandable picture of the dynamics of each species. Comparing the DADS analysis and the SADS analysis, a key difference between the two is that the DADS analysis is a model-free method that does not depend on the kinetic model (although it is still possible to impose model-based constraints on the concentration-related coefficients, $\mathbf{c}_{\mathbf{DADS}}$), whereas the SADS analysis is a model-dependent method that relies on the kinetic model, highlighting a significant distinction between them.

A typical method of extracting SADS is similar to that of extracting DADS, which satisfies eqs. S9–S11. Specifically, in the case of DADS analysis, the analysis extracts DADSs that satisfy the kinetic-model-independent $C_{DADSk'}$ (shown in eq. S9), which depends only on the time constant. In contrast, SADS extraction involves finding SADS that satisfy the kinetic-model-dependent $C_{SADSk'}$. Using $C_{SADSk'}$, SADSs that satisfy the equation $\Delta S_0(q, t) = \mathbf{SADS'}(q) \times \mathbf{C_{SADS}}(t)^T$ are being extracted (Supplementary Fig. 12), where $\mathbf{C_{SADS}}(t)$ is the matrix of the concentration profile, $C_{SADSk'}$. However, it should be noted here that SADS analysis can also be performed using a different approach. Since we previously assigned the structural transitions associated with each time constant in the DADS analysis and refined the structure of each species, we can use the results of the DADS analysis to compute the theoretical SADSs corresponding to each species, $\mathbf{SADS'}(q)$. Thereafter, using $\mathbf{SADS'}(q)$, the time-dependent linear coefficients, or the concentration profile, $\mathbf{C_{SADS}}(t)$, can be extracted from $\Delta S_0(q, t)$. We employed this latter method in our analysis.

The resulting $\mathbf{C_{SADS}}(t)$ matrix was fitted with the sum of five Gaussian-convoluted exponential functions, collectively represented as $\mathbf{C_{SADS}}^{\mathbf{LCF}}(t)$ (Fig. 3e). In this approach, we found that the single time constant from the DADS analysis (130 fs) fails to describe the ultrafast I• dissociation from the *anti* and *gauche* $C_2F_4I_2$ occurring at two distinct kinetics. To solve the issue, we made use of two freely parametrized time constants for the generation of $\mathbf{C_{SADS}}^{\mathbf{LCF}}(t)$, each corresponding to the I• dissociation from *anti*-$C_2F_4I_2$ or *gauche*-$C_2F_4I_2$ before 1.2 ps.

Suppl. Note 5. Analysis of the coherent atomic motions related to primary dissociation

In this section, we briefly explain how we resolved the discrepancy between experimental and simulated TRXL curves within the first few hundreds of femtoseconds. In this ultrafast temporal range, simulated TRXL curves based on the exponential-kinetics-based LCF model could not fully reproduce the experimental data (Supplementary Fig. 13). Similar deviations were observed between experimental and fitted second RSVs (Fig. 2), underscoring the need to consider coherent atomic motions.

To address this, we incorporated atomic motions into the model by iteratively optimizing three structural parameters (r_{CI}, θ_{CCI}, θ_{pl}) at each time delay. In other words, these structural parameters are assumed to evolve from the values of $C_2F_4I_2$ and converge to those of C_2F_4I•. Among them, the carbon–iodine distance, which had the largest impact on improving the fit, is shown in Supplementary Fig. 14, along with a visual comparison of the simulated curves before and after considering the atomic motions.

We also attempted to iteratively optimize φ_{dihed} as a function of time but did not observe any meaningful improvement in the quality of fit.

Suppl. Note 6. Theoretical investigations

Suppl. Note 6-1. Calculation of potential energy curves (PECs)

Initially, to explore the nature of excited states, extended multi-state complete active space second-order perturbation (XMS-CASPT2) corrections[15] for the SA(9/11)-CASSCF wavefunctions (9 singlets and 11 triplets) were performed. Two sets of $\boldsymbol{\sigma}$ and $\boldsymbol{\sigma}^*$ orbitals localized in the C–I bond and two sets of p orbitals localized in the iodine atoms were utilized for the active space (12e, 8o) of *anti* and *gauche* $C_2F_4I_2$. From the optimized structure with the XMS-CASPT2 level of theory, the excitation energies and oscillator strengths to the excited states are calculated. To obtain further information on excited states along the bond dissociation pathways, potential energy curves (PECs) with respect to the C–I distance were calculated. For a better description of the shape of PECs near the conical intersection, we performed a semi-diabatizaion procedure with careful looking at orbital occupation numbers and the smoothness of curves.

We also computed the PECs with respect to the dihedral angle of $C_2F_4I_2$ and $C_2F_4I\bullet$ to describe the rotational isomerization. Firstly, the PECs with respect to the dihedral angle (ICCI) of $C_2F_4I_2$ were calculated by scanning along the dihedral angle (ICCI) while optimizing the rest of the structures with three different levels (CCSD, XMS-CASPT2, and DFT (ωB97X functional)). The energies of ground states were calculated and reported. To estimate the theoretical time constants associated with the rotational isomerization of $C_2F_4I\bullet$ following ultrafast C–I bond dissociation, the XMS-CASPT2 calculation was performed. For $C_2F_4I\bullet$, (7e, 5o) was used for the active space, which contains $\boldsymbol{\sigma}$ and $\boldsymbol{\sigma}^*$ orbitals of the C–I bond, two p orbitals localized in iodine, and the lone pair of the radical. The geometry optimizations were performed for the *anti* and *gauche* $C_2F_4I\bullet$ and for the well-known two transition states denoted as T1 and T2. Similar to the procedure used for $C_2F_4I_2$, the PECs with respect to the dihedral angle (ICCF) of $C_2F_4I\bullet$ were calculated using three different levels of theories (CCSD, XMS-CASPT2, and DFT). The ICCF dihedral angles were then converted to φ_{dihed}, corresponding to the ICC• angle, to compare with the same geometry as in $C_2F_4I_2$. Furthermore, the Gibbs free energies and harmonic frequencies for all optimized geometries of $C_2F_4I_2$ and $C_2F_4I\bullet$ were calculated and used for further RRKM theory calculations. We confirmed that optimized *anti* and *gauche* conformers of $C_2F_4I_2$ and $C_2F_4I\bullet$ have no imaginary frequencies, indicating that these structures are at minima.

Additionally, T1 and T2 geometries each have only one imaginary frequency, indicating that these structures are in the transition state. For the calculation of vertical excitation energies, oscillator strengths and PECs of $C_2F_4I_2$ with respect to the C–I distance, the ANO-RCC-VDZP basis set was employed. Due to the high cost of the geometry optimization process for the relaxed PECs scan with respect to bond rotation, the LANL2DZ basis set used for CCSD(T) and XMS-CASPT2 calculations, and the def2-TZVPP basis set was employed for the DFT calculations. The Openmolcas 8.6[16] software was used for the multireference calculations, ORCA 5.0.4[17] software was employed for the CCSD(T) calculations, and Gaussian 16[18] software was utilized for DFT calculations.

Suppl. Note 6-2. Calculation of kinetic constants

We performed the analysis based on Rice-Ramsperger-Kassel-Marcus (RRKM) theory to find out the theoretical kinetic constants for the rotational isomerization of $C_2F_4I_2$ and C_2F_4I•. For both $C_2F_4I_2$ and C_2F_4I•, the potential energy surface (PES) with respect to the dihedral angle implies that the rotational isomerization takes place through the T2 transition state, among the two well-known transition states, T1 and T2. We utilized the following information to calculate the kinetic constants: the optimized molecular structures, vibrational frequencies, electronic energy levels, and free energies for *anti*, *gauche*, T1, and T2 states of $C_2F_4I_2$ and C_2F_4I•. In this context, only the theoretical kinetic constants for the assigned rotational dynamics (*gauche*-to-*anti* for $C_2F_4I_2$, *anti*-to-*gauche* for C_2F_4I•) trespassing through the T2 states were compared with experimentally extracted values in Table 1. All RRKM theory computations were conducted using the *ChemRate*[19-21] software.

Suppl. Note 6-3. Molecular dynamics simulation

Molecular dynamics (MD) simulations were conducted using the MOLDY 2.16e[22] software to model the interaction between each chemical species involved in the reaction and their surrounding solvent molecules. In these simulations, we employed a virtual cubic box composed of one solute molecule surrounded by 512 rigid solvent molecules. The internal structures of each molecule (*anti*-$C_2F_4I_2$, *gauche*-$C_2F_4I_2$, *anti*-C_2F_4I•, *gauche*-C_2F_4I•, and I•) were fixed to the optimized ones from the DFT method, as detailed in the "Density functional theory calculation" section. The partial charge distributions of each molecule were determined by natural population analysis (NPA) on the optimized structures. In the simulation, the molecular dynamics were governed by intermolecular interactions modeled with Coulombic forces and Lennard-Jones potentials. All simulations took place at an ambient

temperature of 300 K and a solvent density of 0.779 g/cm³. We initiated system equilibration via coupling to a Nose-Hoover thermostat over a 20 ps duration. The simulations were conducted within the NVT ensemble, utilizing a time step of 100 as, and trajectories were traced for a duration of 1 ns. From the molecular snapshots obtained from the MD simulation, we statistically retrieved the distributions of pairwise atomic distances, known as pairwise distribution functions (PDFs). Subsequently, the theoretical cage term was calculated based on the PDFs using eq. S5.

Suppl. Note 6-4. Fractions of initially excited, relaxed, and bond-dissociated molecules

By quantitatively analyzing the temperature change of the solution upon photoreaction, we can determine how many molecules are initially excited, how many returns to the ground state, and how many participate in bond dissociation. Here, we first calculated the enthalpy change (ΔH) corresponding to the observed temperature rise at 1 ps of $\Delta T = 1.73$ K (refer to Supplementary Fig. 3c) by using the relation $\Delta H = nC_p\Delta T$, where n is the mole number of molecules within the laser spot, and C_p is the heat capacity of cyclohexane at constant pressure (= 156.4 J mol^{-1} K^{-1}). The source of the observed temperature rise is the heat dissipated during the reaction. Both processes—1) the recovery of the photoexcited molecule to the ground state and 2) bond dissociation—release excess energy in the form of heat, albeit in different quantities. If we express the initially excited *anti*-$C_2F_4I_2$ and *gauche*-$C_2F_4I_2$ as [*anti*-$C_2F_4I_2$]* and [*gauche*-$C_2F_4I_2$]*, respectively, the dissipated amount of heat is a sum of the enthalpy changes for the dissociation path ([*anti*-$C_2F_4I_2$]* → *anti*-C_2F_4I• + I• and [*gauche*-$C_2F_4I_2$]* → *gauche*-C_2F_4I• + I•) and the relaxation path ([*anti*-$C_2F_4I_2$]* → *anti*-$C_2F_4I_2$ and [*gauche*-$C_2F_4I_2$]* → *gauche*-$C_2F_4I_2$). Using the relative enthalpies for the species (*anti*-$C_2F_4I_2$, *gauche*-$C_2F_4I_2$, *anti*-C_2F_4I•, *gauche*-C_2F_4I•, and I•), we can estimate the amount of dissociated and relaxed fractions of $C_2F_4I_2$ upon photon absorption. In this experiment, we found that a total of 6.8 mM (11.4%) of $C_2F_4I_2$ are initially excited by photoexcitation. Subsequently, 4.3 mM (7.2%) relax back to the ground state, whereas 2.5 mM (4.2%) undergo primary iodine dissociation and subsequent structural changes. A comprehensive picture of initial excitation, relaxation, and dissociation pathways is shown in Supplementary Fig. 17.

Suppl. Note 7. Discussions on the rotational isomerization dynamics

Suppl. Note 7-1. Qualitative assessment of the time constants

The time constant of rotational isomerization observed in this study is comparable to those reported in a number of previous studies ranging from several picoseconds to tens of picoseconds.

However, while the time constant of 26 ps for the C–C bond is typical, the time constant of 1.2 ps for the C–C• bond is slightly faster than what is generally expected for single bond rotation. This observation can be understood by considering the nature of rotationally isomerizing molecules.

In $C_2F_4I_2$, the *gauche*-to-*anti* isomerization occurs between molecules in the ground state. Conversely, the *anti*-to-*gauche* isomerization of C_2F_4I• occurs between transiently formed radicals that partially retain the absorbed photon energy. As the 267 nm photon carries the energy of 448 kJ/mol and the primary I dissociation requires ~270 kJ/mol, the remaining energies of several hundreds of kilojoules remains within C_2F_4I• and I•. This residual energy is then used to (1) propel the dissociating iodine from the parent molecule, (2) form vibrationally hot states that eventually dissipate heat to the solvents, and (3) overcome the rotational activation barrier more readily than those starting from the ground state. Here, part (3) could make the rotational isomerization in C_2F_4I• to occur faster than what can be predicted solely on the activation energy required for the ground state *anti*-conformers.

Meanwhile, the reaction rate (k) is determined not only by the activation energy (E_a) but also by the pre-exponential factor (A). The pre-exponential factor depends on various properties, such as the rotational, vibrational, and translational states of a molecule. For example, the rotational moments of inertia of each molecule affect the relative ratio of rotational partition functions between the ground state (either *anti* or *gauche*) and the transition state (TS). The presence of an additional heavy atom in $C_2F_4I_2$, compared to C_2F_4I•, restricts rotational freedom at the TS. This causes a smaller $Z^{TS}{}_{rot}/Z^{GSrot}$ for $C_2F_4I_2$ compared to C_2F_4I•, thereby reducing the pre-exponential factor and slowing down the reaction. The shape and stiffness of the TS also plays a role since a stiffer TS allows a limited number of modes to trespass the TS, resulting in a smaller $Z^{TS}{}_{vib}/Z^{GS}{}_{vib}$, again reducing A and slowing down the reaction.

We emphasize that all the effects described here influence only the pre-exponential factor, A, without affecting the $e^{-Ea/kT}$ term. This explains why the difference in activation barriers (4.0 kcal/mol for $C_2F_4I_2$ and 3.2 kcal/mol for C_2F_4I•) is less pronounced compared to the difference in time constants (1.2 ps and 26 ps), while the RRKM theory yields the values far closer to the experiment (1.15 ps and 20.2 ps).

Suppl. Note 7-2. Comparative discussions on the rotational isomerization in the gas phase

We note that a number of previous time-resolved studies investigating the dynamics of $C_2F_4I_2$ and other similar haloalkanes have not captured rotational isomerization, despite utilizing scattering or

diffraction techniques that are directly sensitive to structures[23-25]. We propose that the key distinction lies in the environment, considering that the previous femtosecond time-resolved studies measured $C_2F_4I_2$ in the gas phase while our study probed $C_2F_4I_2$ dissolved in a solvent. Unlike in the gas phase, where the solute can move freely via Brownian motions, the solution environment confines the solute within the solvent shell. This solvent shell and its interactions with the solute exert a critical influence on not only the dynamics itself but also the sensitivity of each species on the measured signals.

The impact of the solvation shell on the dynamics primarily arises from its stabilizing effect. Here, the degree of stabilization varies for reactants, intermediates, and products, depending on their specific interactions with the solvent. In other words, solvation can preferentially stabilize certain states over the others, modifying the free energy differences and activation barriers, thereby shifting the kinetics and equilibrium ratios between reactants, intermediates, and products. Since cyclohexane is nonpolar and aprotic, it can stabilize (1) the nonpolar solutes (*anti*-$C_2F_4I_2$) only via dispersion forces and (2) the polar solutes additionally via dipole-induced dipole interactions. The stabilization effect is slightly stronger for the species at the TS, as they are more polar than the *anti* and *gauche* conformers. This implies that the activation energy is slightly reduced (and the kinetic rate is slightly increased) in cyclohexane compared to the gas phase. However, the overall stabilization effect would be minimal as dispersion and dipole-induced dipole interactions available in cyclohexane are weak. In contrast, we expect the stabilization effect to be significantly more pronounced in polar solvents, leading to more drastic changes in reaction kinetics compared to the gas phase.

Furthermore, the presence of a solvent shell enhances the sensitivity of scattering data to the structural dynamics of rotational isomerization. For instance, the *anti* and *gauche* isomers of C_2F_4I• only differ by the relative positions of the fluorine atoms, making them less differentiable based solely on the scattering signals from the solute, as in the case in gas-phase studies. In contrast, in solution, the solvent shell surrounding the two conformers have different orientations due to variations in partial charge distribution and molecular conformation. The difference in solvation environments renders the scattering signals of the *anti* and *gauche* conformers differentiable, enabling reliable retrieval of the *anti*-to-*gauche* isomerization dynamics for C_2F_4I•.

Supplementary Figures

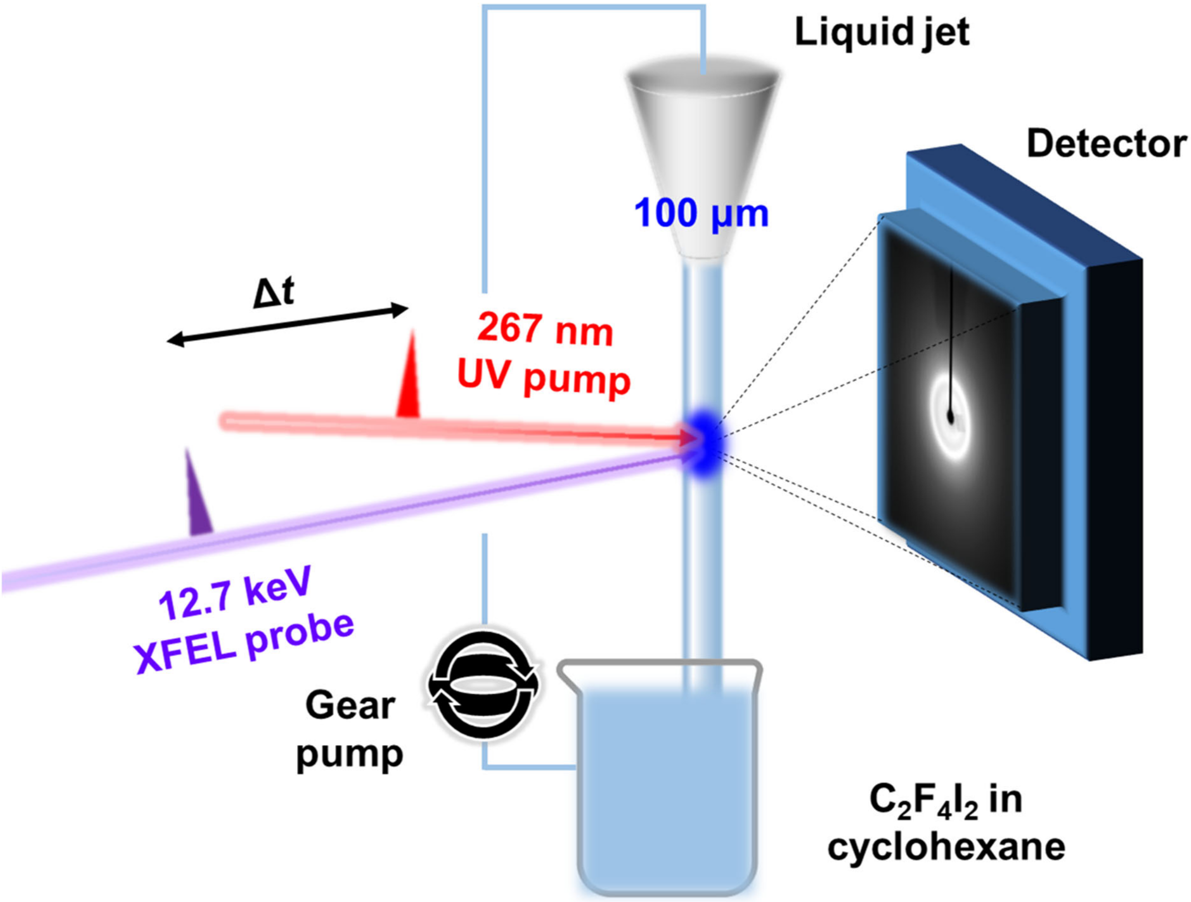

Supplementary Fig. 1 | Schematic design of the TRXL experimental setup. The sample solution containing 60 mM $C_2F_4I_2$ dissolved in cyclohexane was circulated through a gear pump. A capillary jet with a width of 100 µm delivered the solution to the interaction point, where the pump excitation laser pulse at 267 nm (red) and the probe X-ray pulse at 12.7 keV (purple) overlap (blue). When the laser pulse excites the fresh sample of $C_2F_4I_2$, the molecules undergo photoreaction. After a well-defined temporal delay, X-ray scattering images were collected by a two-dimensional area detector. Multiple scattering images were repeatedly measured to improve the signal-to-noise ratio.

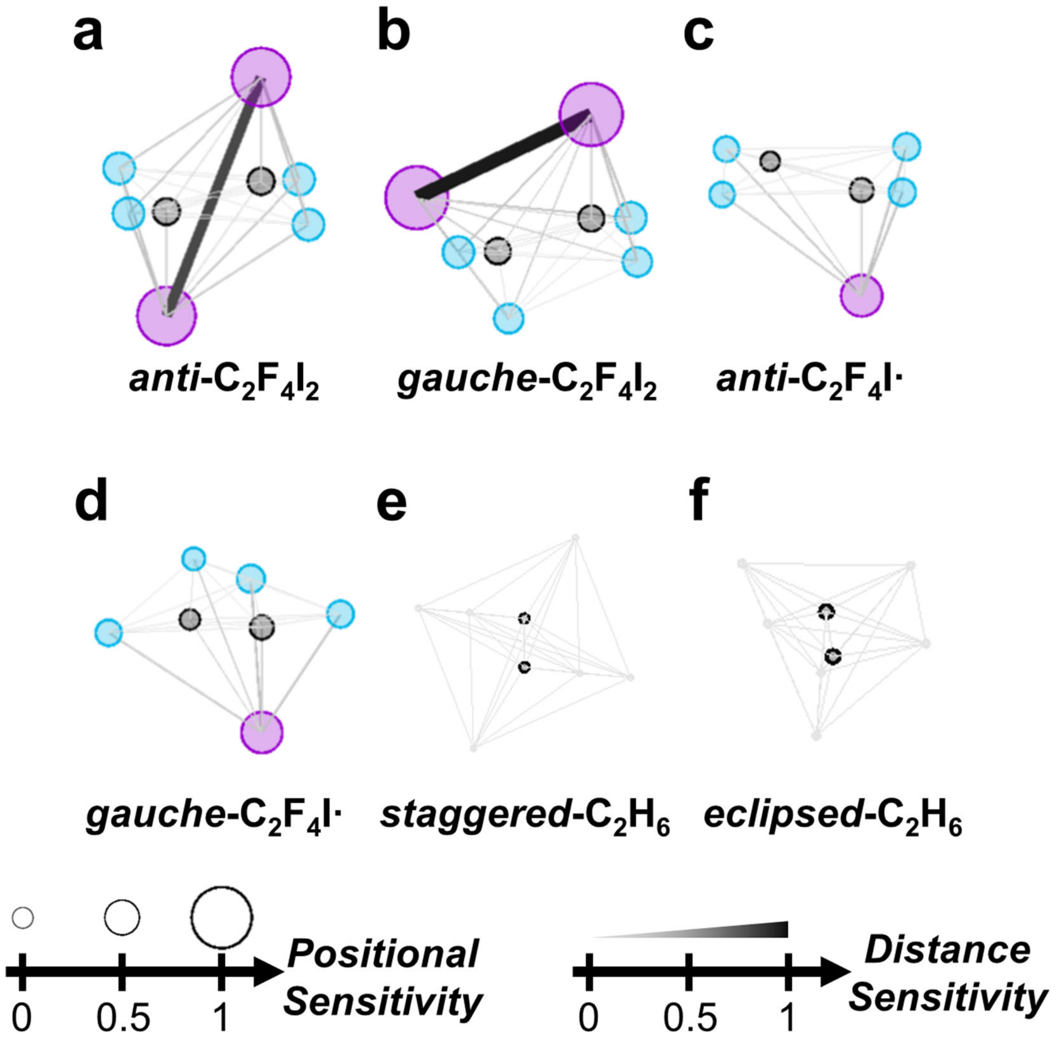

Supplementary Fig. 2 | Sensitivity plots for the isomers of $C_2F_4I_2$, C_2F_4I•, and C_2H_6. The sensitivity plots are visualized for (a) *anti*-$C_2F_4I_2$, (b) *gauche*-$C_2F_4I_2$, (c) *anti*-C_2F_4I•, (d) *gauche*-C_2F_4I•, (e) *staggered*-C_2H_6, and (f) *eclipsed*-C_2H_6. Carbon (C), fluorine (F), iodine (I), and hydrogen (H) atoms are represented by black, cyan, purple, and grey circles, respectively. The area of the circles indicates the sensitivity to atomic positions, while the linewidth and brightness of the lines correspond to the sensitivity to interatomic pair distances. Higher sensitivity is indicated by larger circles or thicker and darker lines.

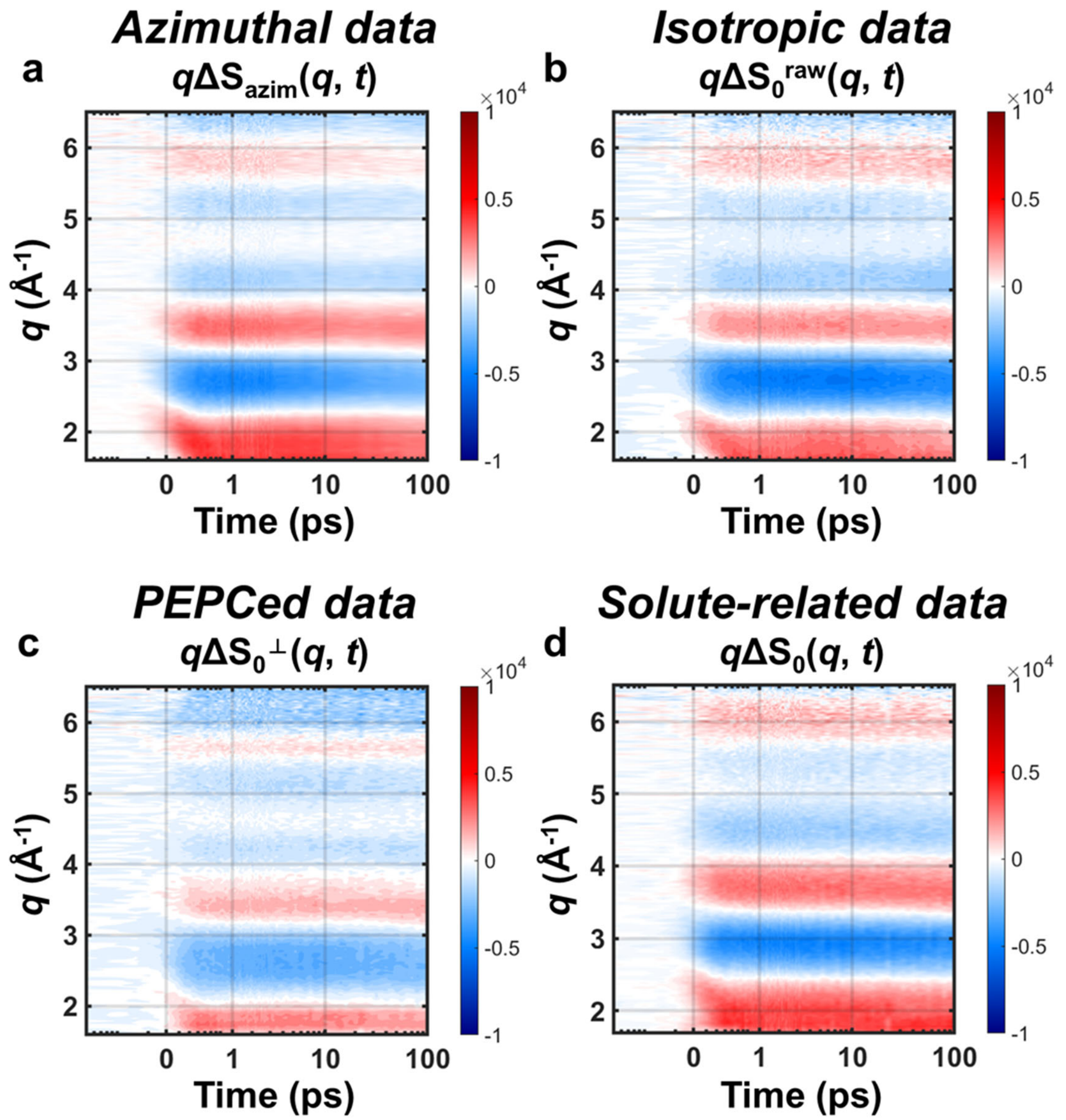

Supplementary Fig. 3 | Isotropic TRXL data of $C_2F_4I_2$ in cyclohexane. (a) The azimuthal data ($q\Delta S_{azim}(q, t)$) obtained through azimuthal integration of the TRXL images. (b) The isotropic data ($q\Delta S_0^{raw}(q, t)$). (c) The PEPCed isotropic data ($q\Delta S_0^{\perp}(q, t)$) using $\mathbf{H}(q)$ as the PEPC components. The PEPC method eliminates the effect of solvent-related kinetics. (d) The solute-related isotropic data ($q\Delta S_0(q, t)$) reconstructed from the model-based analysis.

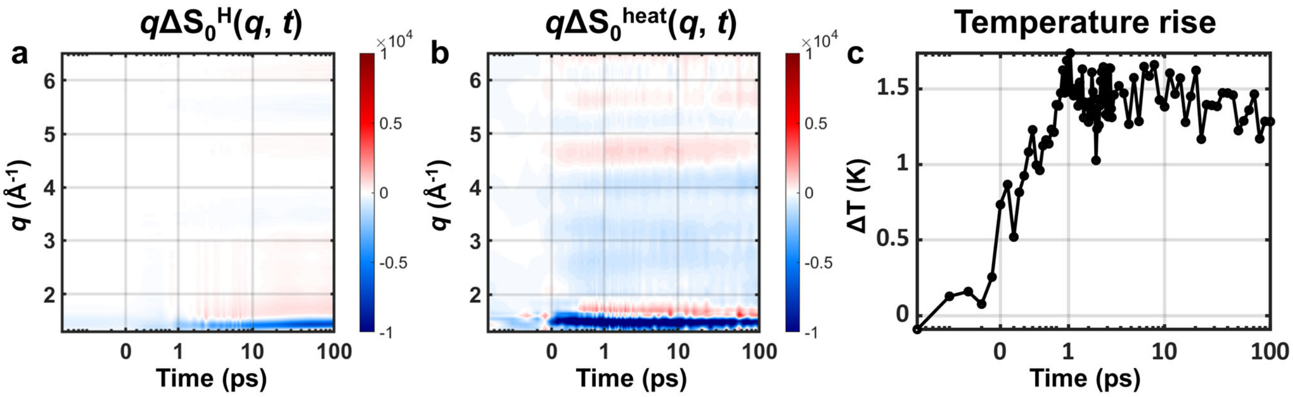

Supplementary Fig. 4 | Solvent term and temperature change in $C_2F_4I_2$ solution in cyclohexane during the photoreaction. (a) The isotropic data corresponding to the solvent response measured in a separate experiment using Azo-Br dye in cyclohexane, denoted as $q\Delta S_0^{H}(q, t)$. (b) The solvent term in the isotropic data of $C_2F_4I_2$ in cyclohexane, denoted as $q\Delta S_0^{heat}(q, t)$. (c) The ultrafast temperature rise of the $C_2F_4I_2$ solution ($\Delta T(t)$) up to 100 ps after photoexcitation, extracted from (b).

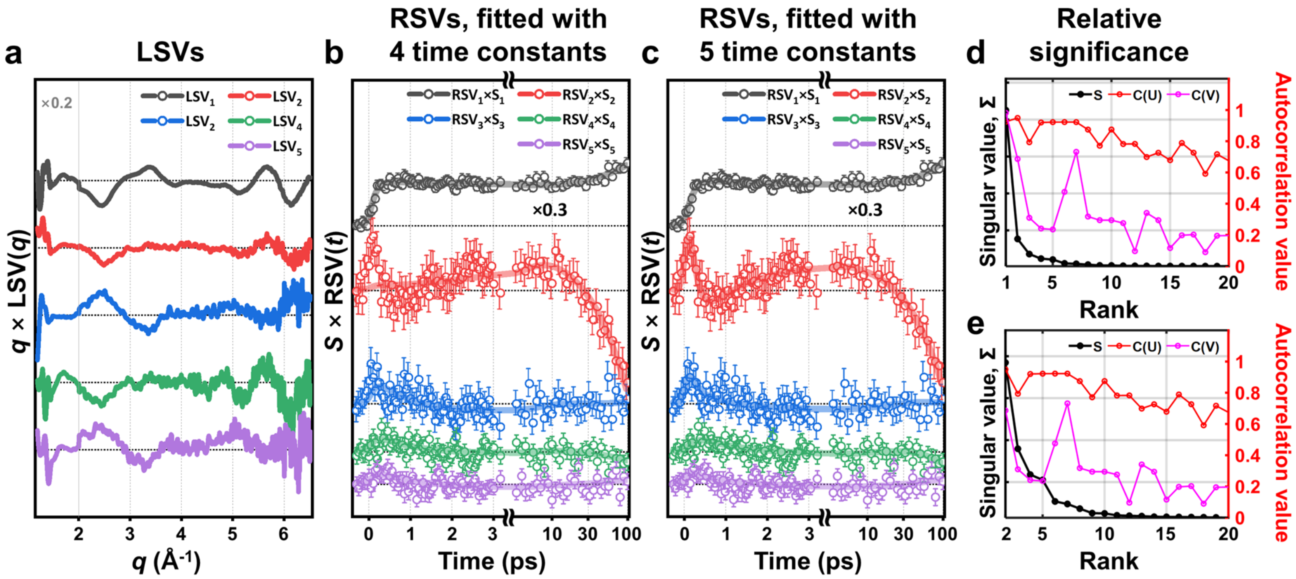

Supplementary Fig. 5 | Singular value decomposition (SVD) of the PEPCed isotropic data, $\Delta S_0^{\perp}(q, t)$, for $C_2F_4I_2$ in cyclohexane. (a) The five major left singular vectors (LSVs). Note that the LSVs are multiplied by *q*, resulting in *q* × LSVs, which are displayed. The amplitude of the LSVs in the region $q < 2$ Å^{-1} was scaled down by a factor of 5 to better visualize the high-*q* data. (b, c) The five major right singular vectors (RSVs) scaled by their respective singular values (**S** × RSVs). The error bars were estimated using the method described in Section 2-3 of the SI, where the LSVs in (a) were used to obtain the time-dependent coefficients (S×RSV) as parameterized variables with standard errors. The **S** × RSVs were fitted with (b) a sum of four Gaussian-convoluted exponential functions and (c) a sum of five Gaussian-convoluted exponential functions. The addition of one ultrafast time constant allows the description of ultrafast behavior in the sub-ps region. (d, e) The singular values (**S**, black), the auto-correlation values for the LSVs (**C**(**U**), red), and the auto-correlation values for the RSVs (**C**(**V**), magenta) up to the 20th rank. In (d), the 1st rank is omitted to better depict the values for the lower ranks. Based on this qualitative evaluation, we concluded that five major components exist in the data.

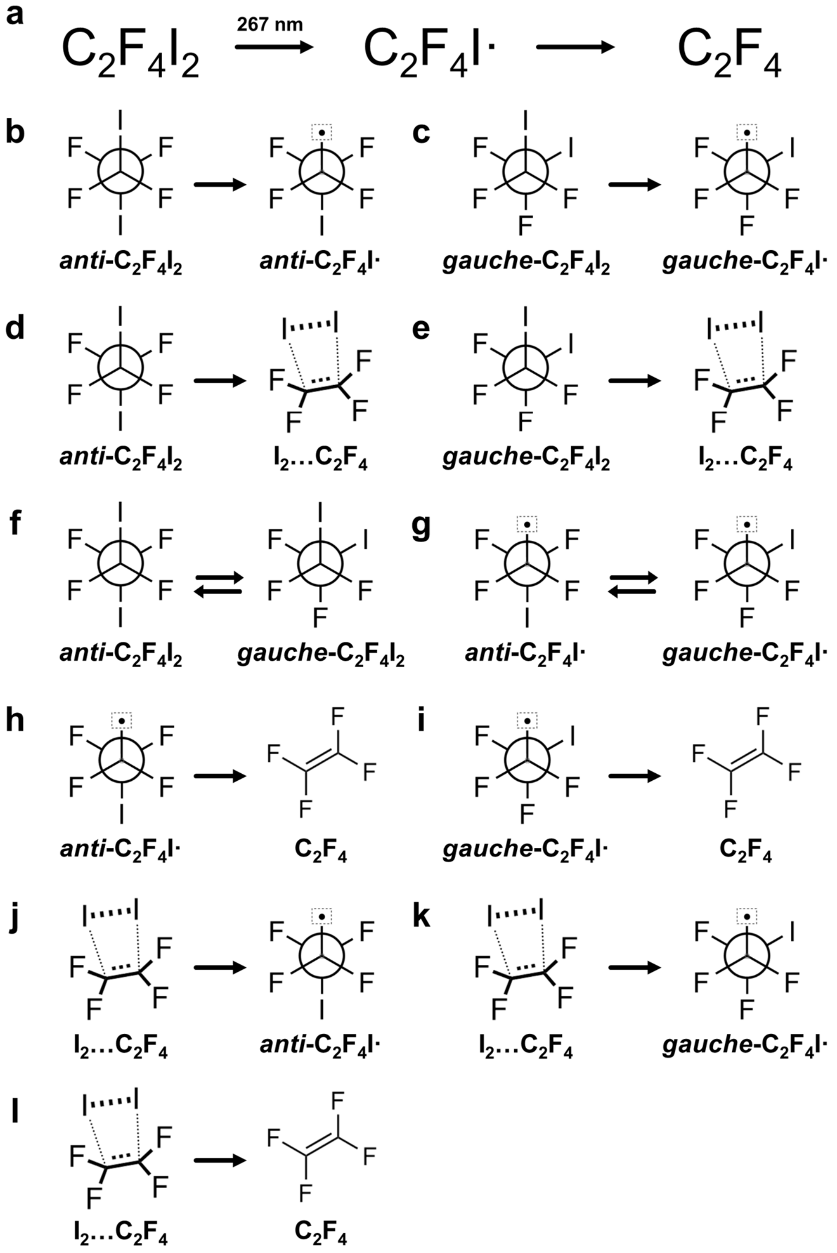

Supplementary Fig. 6 | Candidate structural transitions considered in analyzing $C_2F_4I_2$ dynamics. (a) The known, schematic photoreaction pathways for $C_2F_4I_2$. The photoexcitation of $C_2F_4I_2$ at 267 nm initiates primary I• dissociation, creating the radical intermediate C_2F_4I•, which subsequently undergoes secondary dissociation to form the final product, C_2F_4. To account for all possible dynamic pathways during the reaction, we considered *anti*-$C_2F_4I_2$, *gauche*-$C_2F_4I_2$, *anti*-C_2F_4I•, *gauche*-C_2F_4I•, C_2F_4, and the hypothetical roaming intermediate (I_2…C_2F_4) as the species involved in the reaction. (b–l) The candidate structural transitions connecting these species are depicted. (b) *anti*-$C_2F_4I_2$ → *anti*-C_2F_4I• + I•; (c) *gauche*-$C_2F_4I_2$ → *gauche*-C_2F_4I• + I•; (d) *anti*-$C_2F_4I_2$ → I_2…C_2F_4; (e) *gauche*-$C_2F_4I_2$ → I_2…C_2F_4; (f) *anti*-$C_2F_4I_2$ → *gauche*-$C_2F_4I_2$; (g) *anti*-C_2F_4I• → *gauche*-C_2F_4I•; (h) *anti*-C_2F_4I• → C_2F_4 + I•; (i) *gauche*-C_2F_4I• → C_2F_4 + I•; (j) I_2…C_2F_4 → *anti*-C_2F_4I• + I•; (k) I_2…C_2F_4 → *gauche*-C_2F_4I• + I•; and (l) I_2…C_2F_4 → C_2F_4 + 2I•.

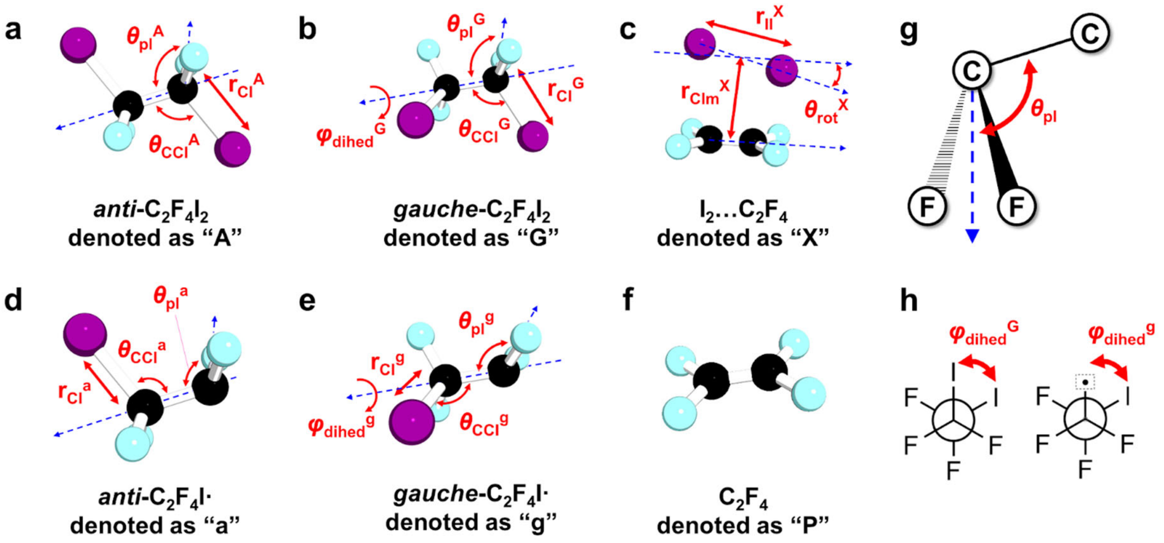

Supplementary Fig. 7 | Definition of structural parameters. The structural parameters defined for the analysis of the TRXL data are shown. The structural parameters allocated for (a) *anti*-$C_2F_4I_2$, (b) *gauche*-$C_2F_4I_2$, (c) $I_2 \ldots C_2F_4$, (d) *anti*-$C_2F_4I\bullet$, (e) *gauche*-$C_2F_4I\bullet$, and (f) C_2F_4 are depicted in the diagram. The superscript notations (A, G, X, a, g, P) designate the species that each structural parameter is involved in. As indicated in the figure, A, G, X, a, g, and P denote *anti*-$C_2F_4I_2$, *gauche*-$C_2F_4I_2$, $I_2 \ldots C_2F_4$, *anti*-$C_2F_4I\bullet$, *gauche*-$C_2F_4I\bullet$, and C_2F_4, respectively. Note that for both *anti* and *gauche* forms of $C_2F_4I_2$, the C–I bond distances ($r_{CI}^{A,G}$) and the CCI angles ($\theta_{CCI}^{A,G}$) were shared for both carbon sites. (g) Definition of the FCF planar angles ($\theta_{pl}^{A,G,a,g}$) from another viewpoint. The FCF planar angle is defined as the angle between the C–C bond and the vector traversing the center of two C–F bonds. (h) The ICCI or •CCI dihedral angles ($\varphi_{dihed}^{A,G,a,g}$) in the Newman projection view. The dihedral angles for the *gauche* structure were parameterized, while those for the *anti* structure were fixed at 180°, as intuitively and computationally expected.

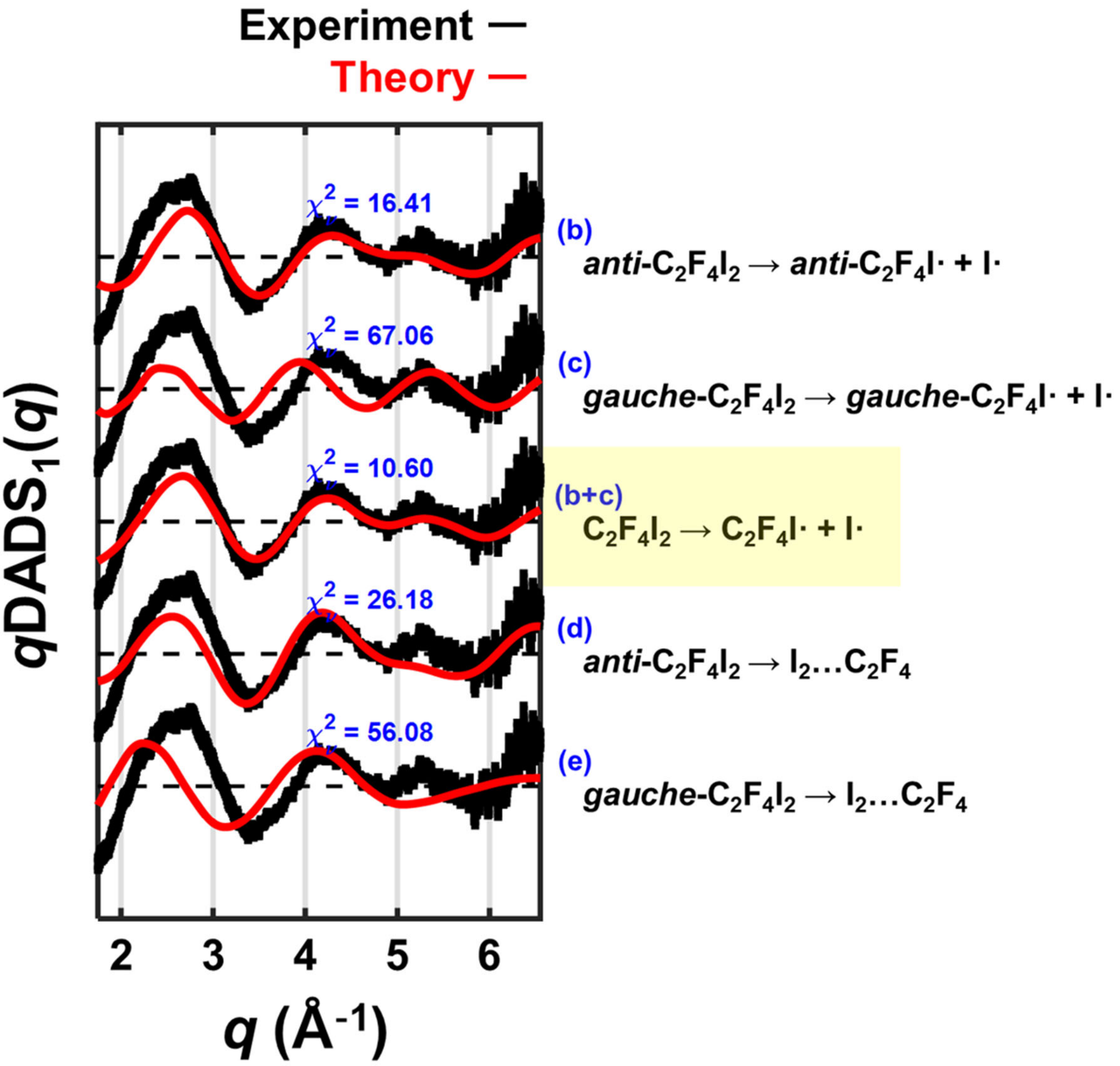

Supplementary Fig. 8 | Determining the correct structural transition for $DADS_1$. The experimental $DADS_1(q)$ (black line) with a time constant of 130 fs was compared with the simulated DADS (red line) corresponding to possible transitions in Supplementary Fig. 6. The chemical equations for candidate transitions used to compare with $DADS_1$ are shown on the right side. Reduced chi-square values (χ_ν^2, in blue texts) were used to quantitatively evaluate the quality of fit among the candidate transitions. As $DADS_1(q)$ represents the earliest dynamics, pathways not starting from $C_2F_4I_2$ ((g–l) in Supplementary Fig. 6) were excluded. The rotational isomerization of $C_2F_4I_2$ ((f) in Supplementary Fig. 6) was also omitted, as dynamic equilibrium must be maintained initially. Note that the simulated DADSs were created by iteratively optimizing r_{CI}, θ_{CCI}, θ_{pl}, and φ_{dihed} near the DFT optimized structures. The candidate transition pathway with the least χ_ν^2 value ($C_2F_4I_2$ to C_2F_4I• and I•, (b)+(c) in Supplementary Fig. 6) is highlighted with a yellow shade.

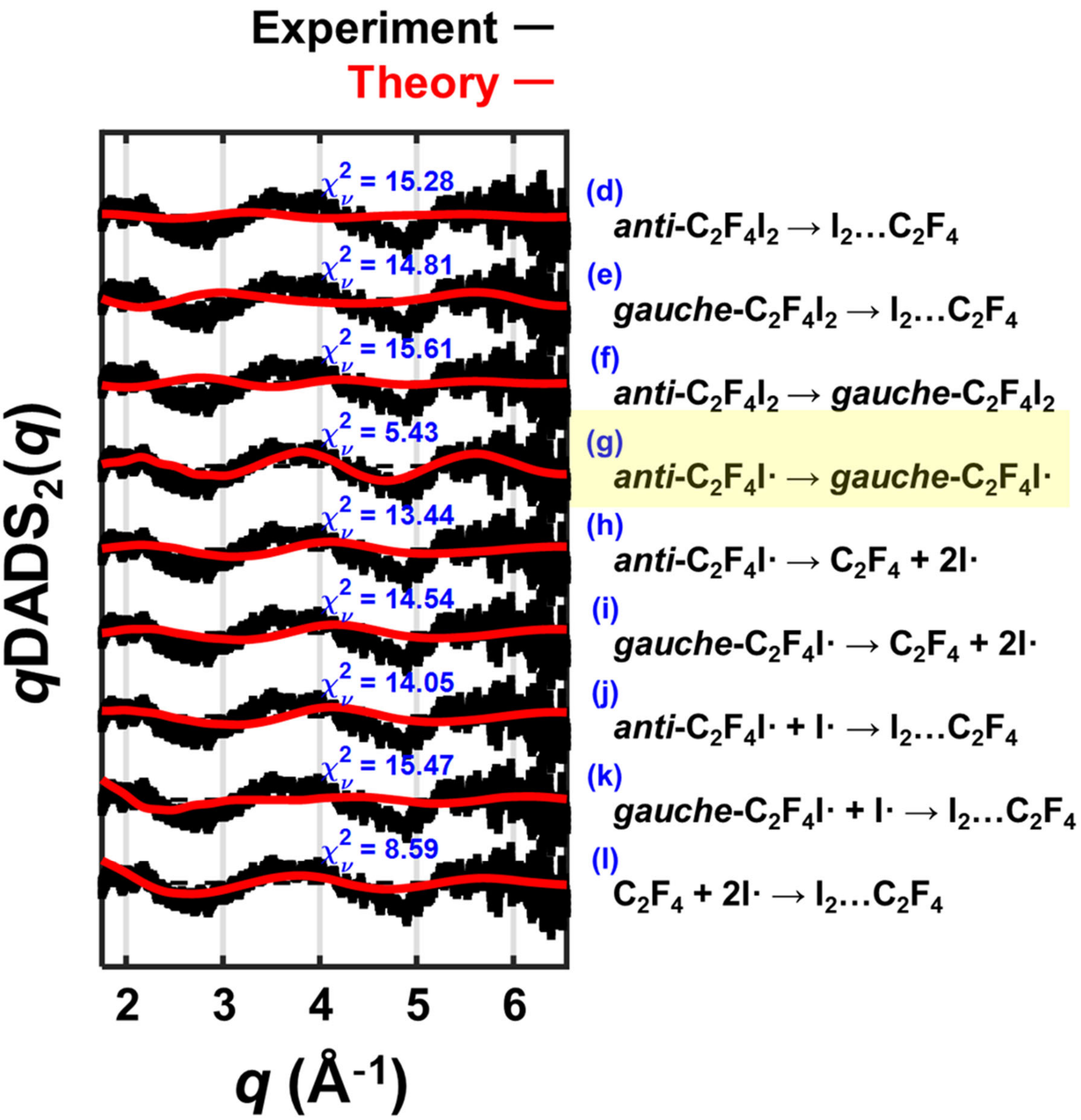

Supplementary Fig. 9 | Determining the correct structural transition for $DADS_2$. The experimental $DADS_2(q)$ (black line) with a time constant of 1.2 ps was compared with the simulated DADS (red line) corresponding to possible transitions in Supplementary Fig. 6. The chemical equations for candidate transitions used to compare with $DADS_2$ are shown on the right side. Reduced chi-square values (χ_ν^2, in blue texts) were used to quantitatively evaluate the quality of fit among the candidate transitions. As $DADS_1(q)$ was already assigned to primary iodine dissociation, the two primary dissociation pathways were excluded from the list of potential candidates. The simulated DADSs were created by iteratively optimizing r_{CI}, θ_{CCI}, θ_{pl}, and φ_{dihed} near the DFT optimized structures. The candidate transition pathway with the least χ_ν^2 value (*anti*-to-*gauche* rotation of C_2F_4I•, (g) in Supplementary Fig. 6) is highlighted with a yellow shade.

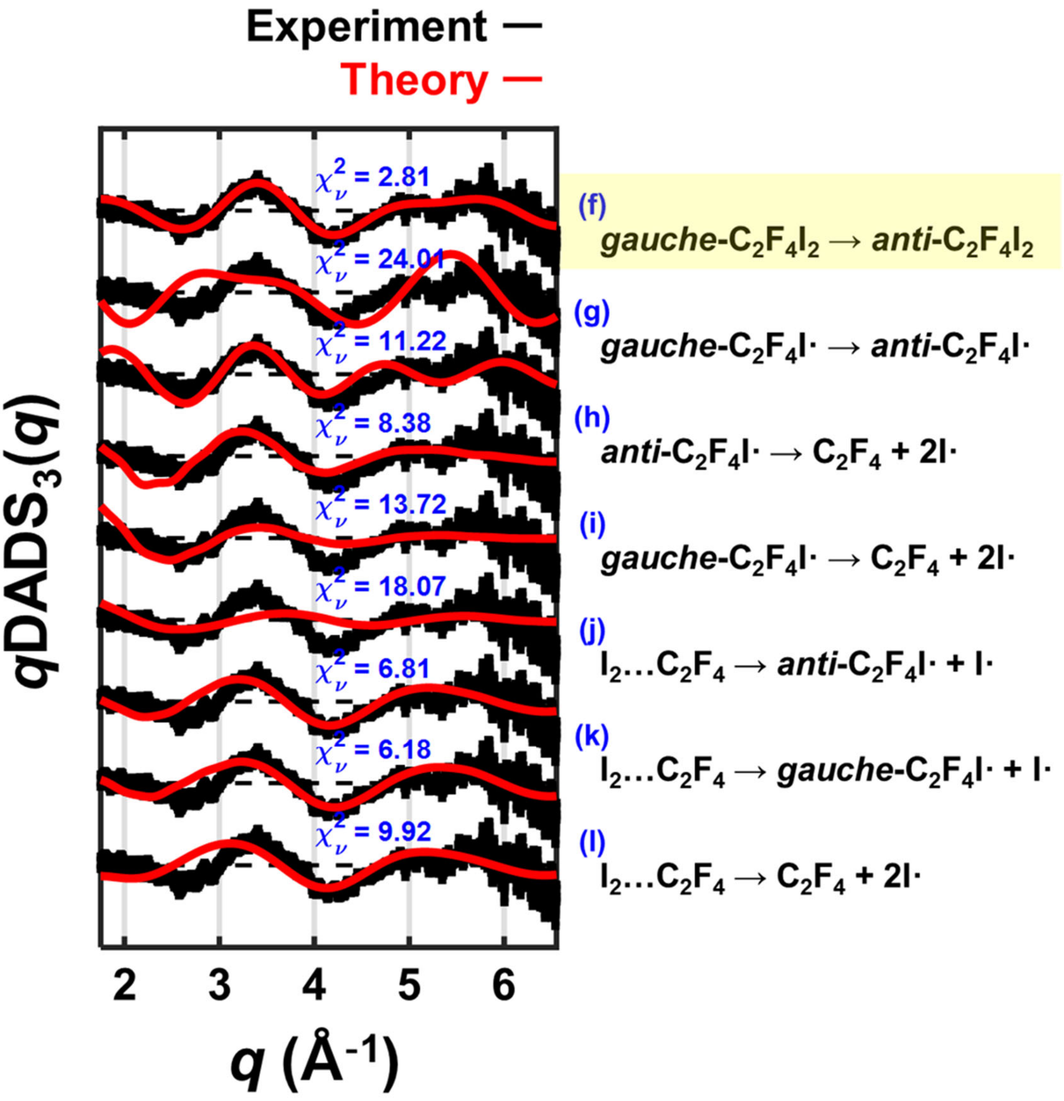

Supplementary Fig. 10 | Determining the correct structural transition for DADS$_3$. The experimental DADS$_3$(q) (black line) with a time constant of 26 ps was compared with the simulated DADS (red line) corresponding to possible transitions in Supplementary Fig. 6. The chemical equations for candidate transitions used to compare with DADS$_3$ are shown on the right side. Reduced chi-square values (χ_ν^2, in blue texts) were used to quantitatively evaluate the quality of fit among the candidate transitions. As DADS$_1$(q) was already assigned to primary iodine dissociation, the two primary dissociation pathways were excluded from the list of potential candidates. Also, we omitted (d) and (e) of Supplementary Fig. 6 since the I_2…C_2F_4 complex does not persist at 100 ps and thus cannot be formed through DADS$_3$(q). The simulated DADSs were created by iteratively optimizing r_{CI}, θ_{CCI}, θ_{pl}, and φ_{dihed} near the DFT optimized structures. The candidate transition pathway with the least χ_ν^2 value (*gauche*-to-*anti* rotation of $C_2F_4I_2$, (f) in Supplementary Fig. 6) is highlighted with a yellow shade.

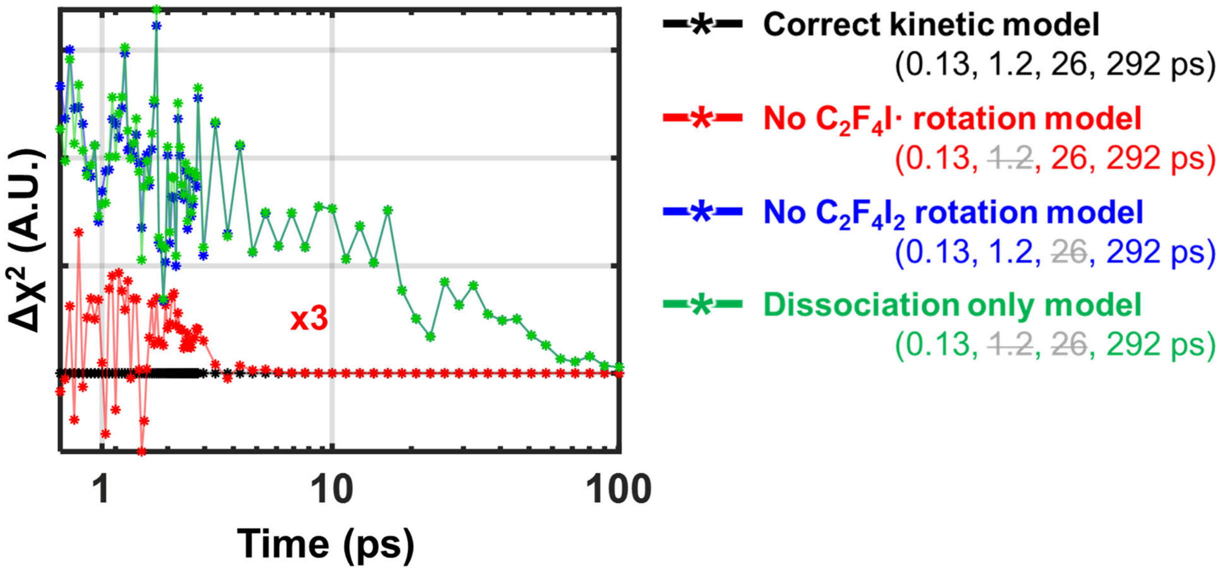

Supplementary Fig. 11 | Statistical comparison of various dynamic models. To evaluate the statistical validity of the two newly observed rotational isomerization dynamics, we examined the changes in χ^2-values when each or both of the dynamics are excluded from the model. Here, we investigated four models: (1) the correct dynamic model (black), (2) a model excluding *anti*-to-*gauche* C_2F_4I• rotation occurring with the time constant of 1.2 ps (red), (3) a model excluding *gauche*-to-*anti* $C_2F_4I_2$ rotation occurring with the time constant of 26 ps (blue), and (4) a model excluding both rotations (green). In the legend, the numbers with strikethrough indicate the excluded time constants in each model. We calculated the χ^2-difference ($\Delta\chi^2$) between the correct model and the models excluding rotational isomerization dynamics. Positive values of $\Delta\chi^2$ indicate that the excluded dynamics contribute meaningfully to the TRXL data. It should be noted that the comparison of $\Delta\chi^2$ in the ultrafast time domain is omitted since the model based on exponential kinetics cannot describe the ultrafast dissociation dynamics (refer to Supplementary Fig. 14 for details).

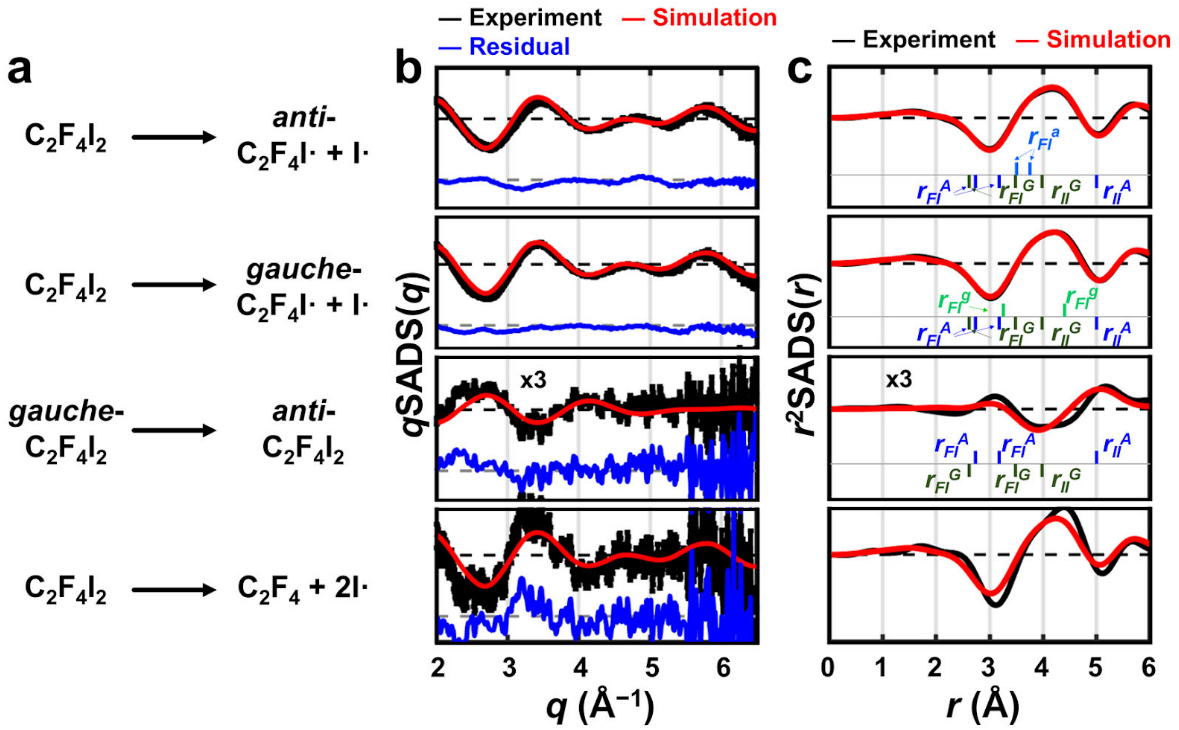

Supplementary Fig. 12 | Ultrafast structural dynamics of carbon–carbon single bond rotation visualized with the kinetics-constrained analysis (KCA). (a) The pair of initial and final states used for generating the species-associated difference scattering curves (SADS(q)). The initial state is the mixture of *anti* and *gauche* $C_2F_4I_2$ for $SADS_1$, $SADS_2$, and $SADS_4$, while the rotational isomerization of $C_2F_4I_2$ is directly modeled for $SADS_3$ (see section 4 of SI for details). (b) The experimental SADSs (black lines, SADS(q)), overlaid with their corresponding simulated SADSs (red lines, $SADS^{KCA}(q)$) corresponding to the four species in (a). The experimental error and fit residuals are shown by vertical bars and blue lines, respectively. (c) The experimental (SADS(r), black) and simulated (SADS′(r), red) SADSs in r-space. The interatomic distances of *anti*-$C_2F_4I_2$, *gauche*-$C_2F_4I_2$, *anti*-C_2F_4I•, and *gauche*-C_2F_4I• were represented with small vertical bars below the SADS, marked with positive and negative signs relative to the reference line. The negative and positive signs of SADS(r) align well with the depletion (marked below the reference line) and formation (marked above the reference line) of interatomic distances, respectively.

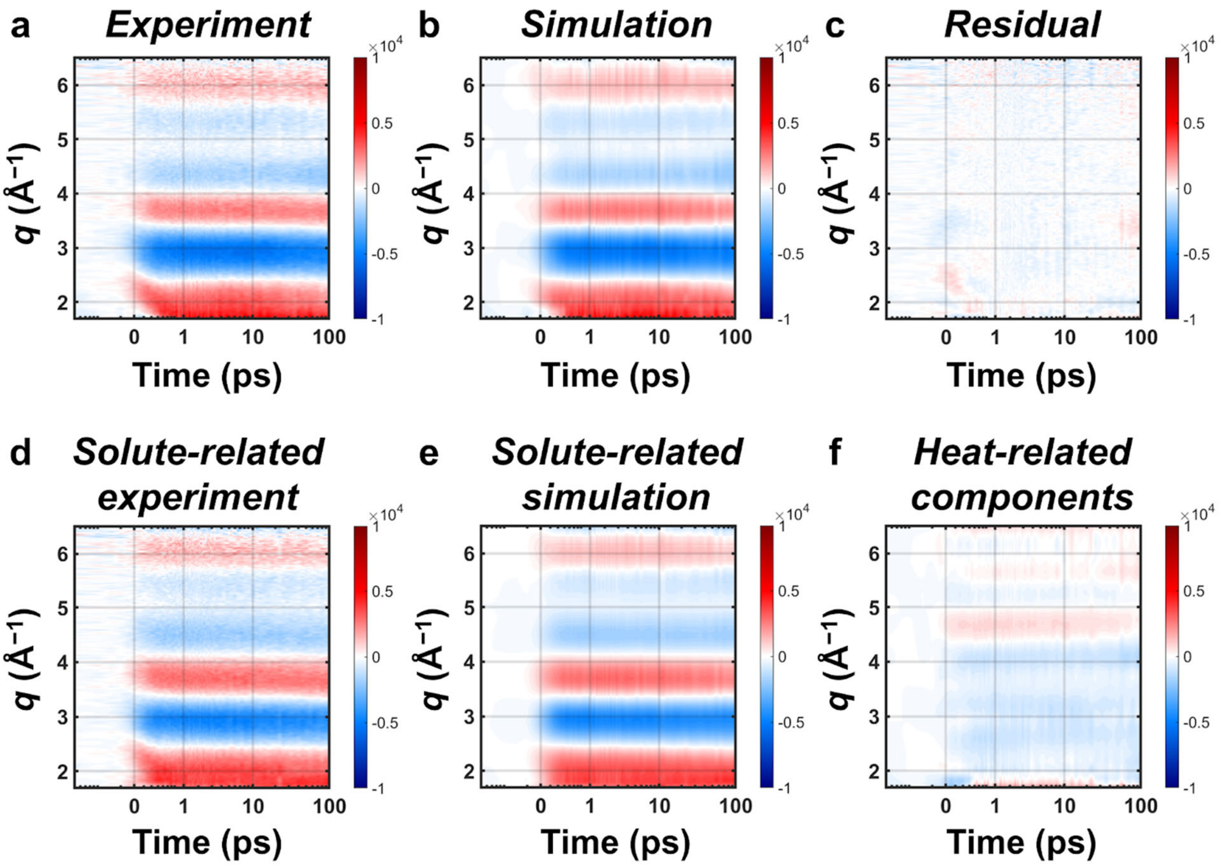

Supplementary Fig. 13 | Comparison of experimental and simulated TRXL data modeled by linear combination fit (LCF) analysis. (a) The experimental TRXL data, denoted as $q\Delta S_0^{\mathrm{raw}}(q, t)$. (b) The simulated TRXL data generated through the LCF of the SADSs, denoted as $q\Delta S_0^{\mathrm{raw}\prime}(q, t)$. (c) The residual map, denoted as $q\Delta S_0^{\mathrm{residual}}(q, t)$, equivalent to $q(\Delta S_0^{\mathrm{raw}} - \Delta S_0^{\mathrm{raw}\prime})$ (and thus $q(\Delta S_0 - \Delta S_0')$). (d) The solute-related experimental TRXL data, denoted as $q\Delta S_0(q, t)$. (e) The solute-related simulated data, denoted as $q\Delta S_0'(q, t)$. (f) The heat-related components of the TRXL data, $q\Delta S_0^{\mathrm{heat}}(q, t)$, retrieved through the PEPC and DADS analysis. A high level of agreement between the experimental and simulated data was confirmed, as observed in both the comparisons of (a) versus (b) and (d) versus (e).

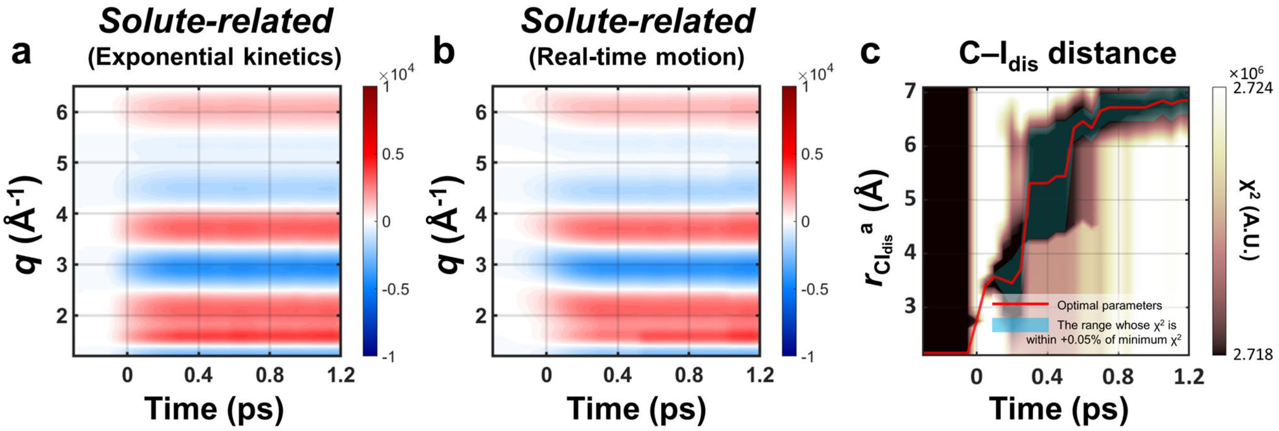

Supplementary Fig. 14 | Ultrafast primary iodine dissociation by modeling the time-dependent evolution of distances between carbon (C) and dissociating iodine (I_{dis}) atoms. (a) The solute-related isotropic data simulated by assuming exponential kinetics for the primary iodine dissociation. (b) The solute-related isotropic data simulated by considering the ultrafast time-dependent changes of C–I_{dis} distance (r_{CIdis}). The inclusion of r_{CIdis} into the model clearly improves the quality of fit of the ultrafast TRXL data before 400 fs, thereby unveiling the origins for the sub-1 ps time constants (in Fig. 2 and Supplementary Fig. 5). (c) The time-dependent C–I_{dis} distance changes in *anti* conformer ($r_{CIdis}{}^{a}(t)$). We note that the color scale in (c) represents the χ^2-values. The first and third quartile of the entire χ^2-values, which correspond to 2.718×10^6 and 2.724×10^6, are selected as the smallest (black) and largest (white) colors, respectively.

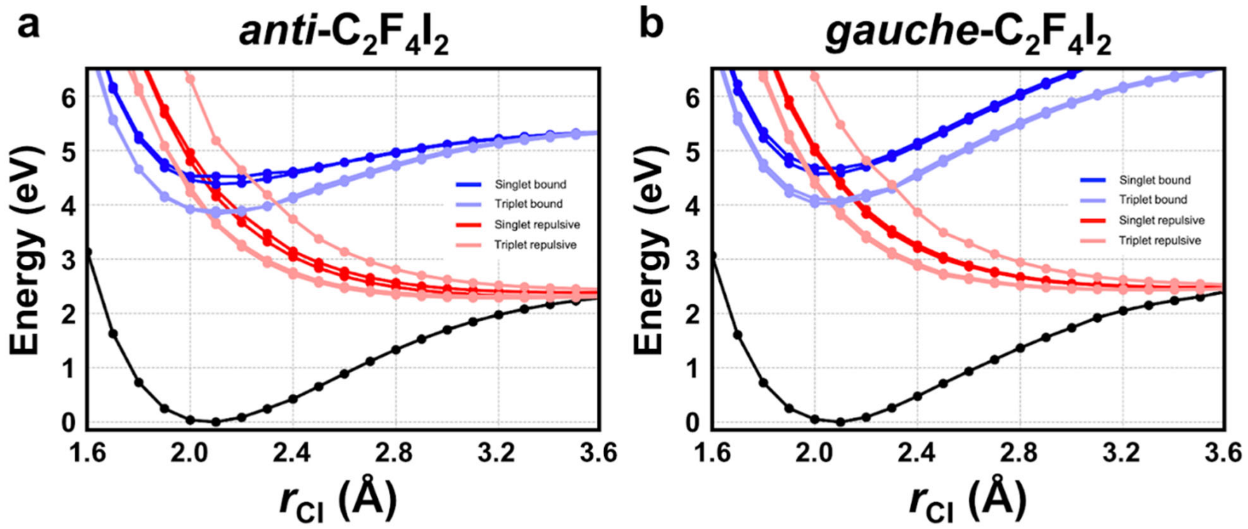

Supplementary Fig. 15 | Diabatized potential energy curves (PECs) with respect to the dissociating C–I bond distance. The PECs for (a) *anti*-$C_2F_4I_2$ and (b) *gauche*-$C_2F_4I_2$ were calculated for the first few singlet and triplet states at the XMS-CASPT2 level of computation. These PECs were diabatized by carefully examining orbital occupations and avoided crossings. The ground state is represented by a black line, bound states are represented in blue, and repulsive states are represented in red. In both figures, the singlet states and triplet states are differentiated by vivid and pale colors, respectively. For both *anti* and *gauche* conformers, we found that the shapes of repulsive states, which directly influence the dissociation dynamics, are similar to each other.

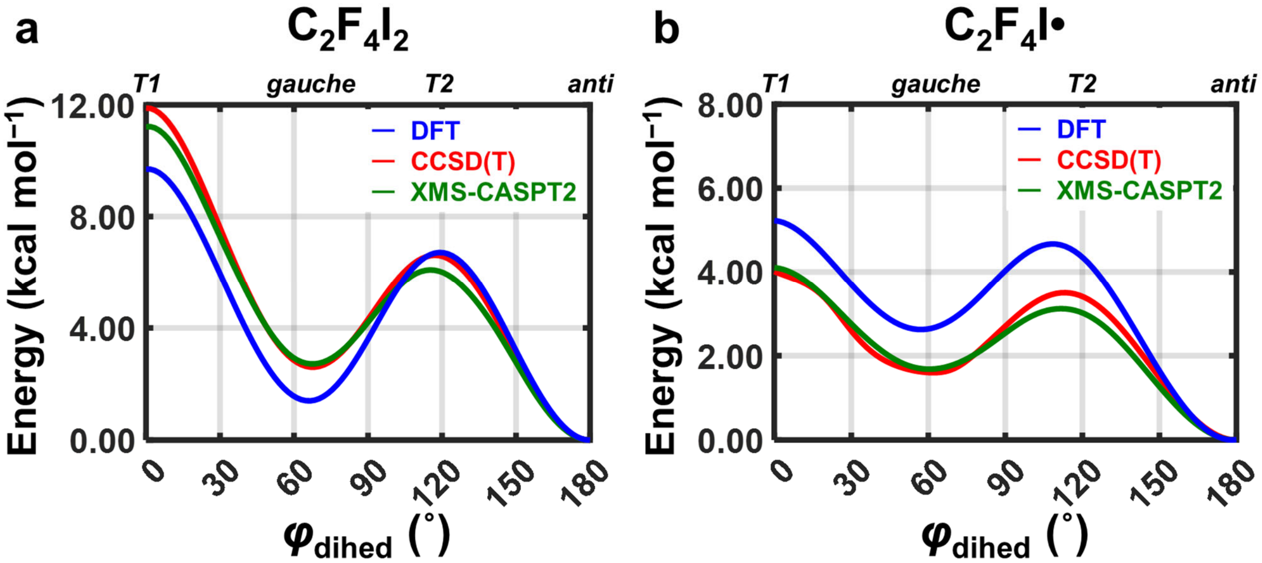

Supplementary Fig. 16 | Potential energy curves (PECs) with respect to the dihedral angle. The PECs for (a) $C_2F_4I_2$ and (b) C_2F_4I•. Both PECs were obtained through a relaxed scan while constraining the dihedral angles (ICCI angle for $C_2F_4I_2$ and ICCF angle for C_2F_4I•). Calculations were performed using three different methods: CCSD(T) (black), XMS-CASPT2 (red), and DFT (green). The PECs obtained with CCSD(T) and XMS-CASPT2 showed similarities, whereas those calculated with DFT (ωB97X / def2-TZVPP) exhibited notable deviations compared to the other two methods. Note that the ICCF angle of C_2F_4I• is transformed to the ICC• angle for visualization, allowing its dihedral angle to be compared to that of $C_2F_4I_2$.

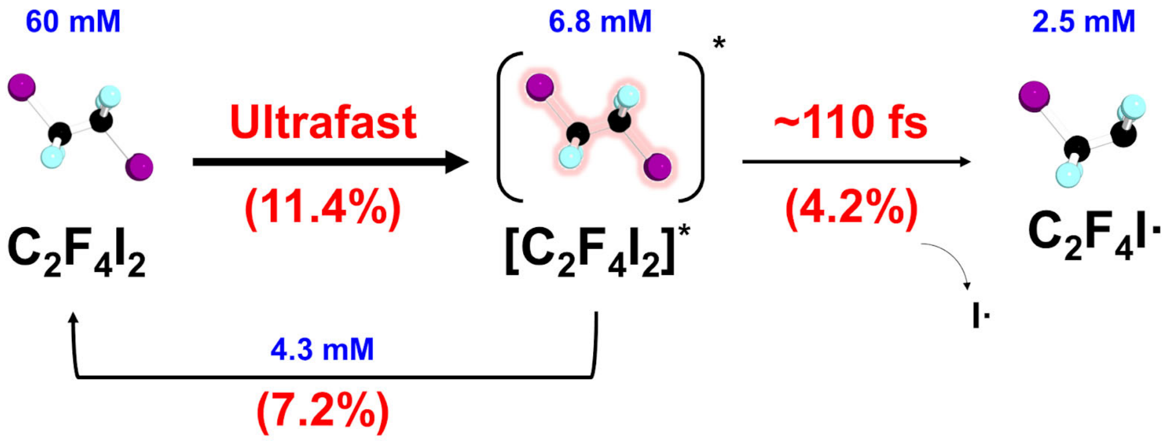

Supplementary Fig. 17 | Fraction of initially excited, relaxed, and dissociated $C_2F_4I_2$ molecules. Upon irradiation of an optical pump pulse, 6.8 mM of $C_2F_4I_2$ are excited to the Franck-Condon region, denoted by an asterisk (*). This amount corresponds to 11.4% of the total $C_2F_4I_2$ molecules in solution. Subsequently, 4.3 mM of the excited molecules ($[C_2F_4I_2]^*$) relax back to the ground state without undergoing bond dissociation. This amount corresponds to 7.2% of the total $C_2F_4I_2$ molecules. Meanwhile, 2.5 mM of the excited molecules undergo primary iodine dissociation with an approximated time constant of 110 fs. This amount corresponds to 4.2% of the total $C_2F_4I_2$ molecules. In this diagram, we represent only the earliest part of dynamics, omitting the dynamics of rotational isomerization or secondary dissociation for clarity.

Supplementary Tables

Supplementary Table 1 | Calculated harmonic frequencies of $C_2F_4I_2$ and C_2F_4I•. We calculated vibrational eigenmodes of $C_2F_4I_2$ and C_2F_4I• in their *anti*, *gauche*, and two transition state conformers (TS1, TS2). The negative frequencies arising in the transition state (TS) conformers are highlighted with gray shades. These values were used to estimate the theoretical kinetic constants for single bond rotation dynamics.

	$C_2F_4I_2$				C_2F_4I·			
	anti	***gauche***	**TS1**	**TS2**	***anti***	***gauche***	**TS1**	**TS2**
Vibrational frequencies (cm^{-1})	49.94	51.71	−59.30	−43.36	62.67	52.01	−57.15	−55.35
	99.02	90.29	92.73	119.55	120.49	131.21	138.22	143.79
	128.97	151.69	172.99	144.50	198.54	211.60	232.09	236.67
	205.29	229.78	232.65	235.05	245.40	243.63	248.04	241.93
	242.89	233.87	246.59	243.23	246.22	250.57	251.85	262.30
	254.47	264.84	251.32	264.79	329.06	276.01	296.01	334.72
	267.12	266.21	260.39	273.16	466.97	388.86	447.67	350.24
	272.47	289.30	304.37	299.47	484.14	489.22	471.18	500.19
	329.03	303.41	338.98	358.11	532.57	552.09	487.39	580.13
	476.47	406.64	453.16	393.03	594.17	712.83	700.89	610.47
	490.64	512.44	504.18	515.53	872.71	866.28	825.83	863.16
	594.20	561.03	509.27	583.93	1009.95	958.88	978.61	986.89
	654.33	783.70	796.41	708.33	1033.31	1020.31	1027.17	1002.58
	974.13	921.57	855.64	940.91	1142.38	1132.48	1137.50	1137.94
	1000.61	992.24	978.85	990.61	1264.41	1258.25	1247.40	1247.55
	1002.00	1012.18	1008.76	1014.81				
	1017.48	1034.60	1014.44	1017.46				
	1165.73	1137.80	1187.40	1098.67				

Supplementary References

1. Kieffer, J., Valls, V., Blanc, N. & Hennig, C. New tools for calibrating diffraction setups. *J. Synchrotron Rad.* **27**, 558-566 (2020). https://doi.org/10.1107/S1600577520000776
2. Ki, H., Gu, J., Cha, Y., Lee, K. W. & Ihee, H. Projection to extract the perpendicular component (PEPC) method for extracting kinetics from time-resolved data. *Struct. Dyn.* **10**, 034103 (2023). https://doi.org/10.1063/4.0000189
3. Lee, Y. *et al.* Cerium photocatalyst in action: Structural dynamics in the presence of substrate visualized via time-resolved x-ray liquidography. *J. Am. Chem. Soc.* **145**, 23715-23726 (2023). https://doi.org/10.1021/jacs.3c08166
4. Biasin, E. *et al.* Anisotropy enhanced X-ray scattering from solvated transition metal complexes. *J. Synchrotron Rad.* **25**, 306-315 (2018). https://doi.org/10.1107/S1600577517016964
5. Cammarata, M. *et al.* Impulsive solvent heating probed by picosecond x-ray diffraction. *J. Chem. Phys.* **124**, 124504 (2006). https://doi.org/10.1063/1.2176617
6. Duguay, M. A. & Hansen, J. W. An ultrafast light gate. *Appl. Phys. Lett.* **15**, 192-194 (1969). https://doi.org/10.1063/1.1652962
7. Ho, P. P. & Alfano, R. R. Optical Kerr effect in liquids. *Phys. Rev. A* **20**, 2170-2187 (1979). https://doi.org/10.1103/PhysRevA.20.2170
8. Righini, R. Ultrafast optical Kerr Effect in liquids and solids. *Science* **262**, 1386-1390 (1993).
9. Ki, H. *et al.* Optical Kerr effect of liquid acetonitrile probed by femtosecond time-resolved X-ray liquidography. *J. Am. Chem. Soc.* **143**, 14261-14273 (2021). https://doi.org/10.1021/jacs.1c06088
10. Kjær, K. S. *et al.* Introducing a standard method for experimental determination of the solvent response in laser pump, X-ray probe time-resolved wide-angle X-ray scattering experiments on system in solution. *Phys. Chem. Chem. Phys.* **15**, 15003-15016 (2013).
11. Choi, E. H. *et al.* Filming ultrafast roaming-mediated isomerization of bismuth triiodide in solution. *Nat. Commun.* **12**, 4732 (2021). https://doi.org/10.1038/s41467-021-25070-z
12. Gu, J. *et al.* Structural dynamics of C2F4I2 in cyclohexane studied via time-resolved x-ray liquidography. *Int. J. Mol. Sci.* **22**, 9793 (2021). https://doi.org/10.3390/ijms22189793
13. Debye, P. Zerstreuung von Röntgenstrahlen. *Ann. Phys.* **351**, 809-823 (1915). https://doi.org/10.1002/andp.19153510606
14. Kim, T. K., Lee, J. H., Wulff, M., Kong, Q. & Ihee, H. Spatiotemporal kinetics in solution studied by time-resolved x-ray liquidography (solution scattering). *ChemPhysChem* **10**, 1958-1980 (2009). https://doi.org/10.1002/cphc.200900154
15. Shiozaki, T., Győrffy, W., Celani, P. & Werner, H.-J. Communication: Extended multi-state complete active space second-order perturbation theory: Energy and nuclear gradients. *J. Chem. Phys.* **135** (2011). https://doi.org/10.1063/1.3633329
16. Fdez. Galván, I. *et al.* OpenMolcas: From source code to insight. *J. Chem. Theory Comput.* **15**, 5925-5964 (2019). https://doi.org/10.1021/acs.jctc.9b00532
17. Neese, F., Wennmohs, F., Becker, U. & Riplinger, C. The ORCA quantum chemistry program package. *J. Chem. Phys.* **152** (2020). https://doi.org/10.1063/5.0004608
18. Gaussian 16 Rev. C.01 (Wallingford, CT, 2016).
19. Bedanov, V. M., Tsang, W. & Zachariah, M. R. Master equation analysis of thermal activation reactions: Reversible isomerization and decomposition. *J. Phys. Chem.* **99**, 11452-11457 (1995). https://doi.org/10.1021/j100029a024
20. Tsang, W., Bedanov, V. & Zachariah, M. R. Master equation analysis of thermal activation reactions: Energy-transfer constraints on falloff behavior in the decomposition of reactive intermediates with low thresholds. *J. Phys. Chem.* **100**, 4011-4018 (1996). https://doi.org/10.1021/jp9524901
21. Knyazev, V. D. & Tsang, W. Chemically and thermally activated decomposition of secondary butyl radical. *J. Phys. Chem. A* **104**, 10747-10765 (2000). https://doi.org/10.1021/jp001921z
22. Refson, K. Moldy: a portable molecular dynamics simulation program for serial and parallel computers. *Comput. Phys. Commun.* **126**, 310-329 (2000). https://doi.org/10.1016/S0010-4655(99)00496-8
23. Ihee, H., Goodson, B. M., Srinivasan, R., Lobastov, V. A. & Zewail, A. H. Ultrafast electron diffraction and structural dynamics: Transient intermediates in the elimination reaction of $C_2F_4I_2$. *J. Phys. Chem. A* **106**, 4087-4103 (2002). https://doi.org/10.1021/jp014144r

24. Reckenthaeler, P. *et al.* Time-resolved electron diffraction from selectively aligned molecules. *Phys. Rev. Lett.* **102**, 213001 (2009). https://doi.org/10.1103/Physrevlett.102.213001
25. Wilkin, K. J. *et al.* Diffractive imaging of dissociation and ground-state dynamics in a complex molecule. *Phys. Rev. A* **100**, 023402 (2019). https://doi.org/10.1103/PhysRevA.100.023402
