# Supplementary material for: Ultrafast structural dynamics of carbon–carbon single-bond rotation in transient radical species at non-equilibrium
Source: Nat Commun. 2025 Feb 25;16:1969. doi: 10.1038/s41467-025-57279-7 (PMC11861307; doi:10.1038/s41467-025-57279-7)
Supplement: Supplementary file 2 — Description of Additional Supplementary Files [file 41467_2025_57279_MOESM2_ESM.pdf]

Description of Additional Supplementary Files:

Supplementary Data 1: Computationally optimized molecular structures for the five species (*anti*-$C_2F_4I_2$, *gauche*-$C_2F_4I_2$, *anti*-C_2F_4I•, *gauche*-C_2F_4I•, and I•)
