# Supplementary material for: Ultrafast structural dynamics of carbon–carbon single-bond rotation in transient radical species at non-equilibrium
Source: Nat Commun. 2025 Feb 25;16:1969. doi: 10.1038/s41467-025-57279-7 (PMC11861307; doi:10.1038/s41467-025-57279-7)
Supplement: Supplementary file 4 — Reporting Summary [file 41467_2025_57279_MOESM4_ESM.pdf]

nature portfolio

Corresponding author(s): Hyotcherl Ihee

Last updated by author(s): Jan 24, 2025

Reporting Summary

Nature Portfolio wishes to improve the reproducibility of the work that we publish. This form provides structure for consistency and transparency in reporting. For further information on Nature Portfolio policies, see our Editorial Policies and the Editorial Policy Checklist.

Statistics

For all statistical analyses, confirm that the following items are present in the figure legend, table legend, main text, or Methods section.

n/a	Confirmed	
☐	☒	The exact sample size (n) for each experimental group/condition, given as a discrete number and unit of measurement
☐	☒	A statement on whether measurements were taken from distinct samples or whether the same sample was measured repeatedly
☐	☒	The statistical test(s) used AND whether they are one- or two-sided<br>*Only common tests should be described solely by name; describe more complex techniques in the Methods section.*
☐	☒	A description of all covariates tested
☐	☒	A description of any assumptions or corrections, such as tests of normality and adjustment for multiple comparisons
☐	☒	A full description of the statistical parameters including central tendency (e.g. means) or other basic estimates (e.g. regression coefficient) AND variation (e.g. standard deviation) or associated estimates of uncertainty (e.g. confidence intervals)
☒	☐	For null hypothesis testing, the test statistic (e.g. F, t, r) with confidence intervals, effect sizes, degrees of freedom and P value noted<br>*Give P values as exact values whenever suitable.*
☒	☐	For Bayesian analysis, information on the choice of priors and Markov chain Monte Carlo settings
☐	☒	For hierarchical and complex designs, identification of the appropriate level for tests and full reporting of outcomes
☒	☐	Estimates of effect sizes (e.g. Cohen's d, Pearson's r), indicating how they were calculated

Our web collection on statistics for biologists contains articles on many of the points above.

Software and code

Policy information about availability of computer code

Data collection	We used python-based data collection macros provided by PAL-XFEL to collect the TRXL data.
Data analysis	We used public functions and libraries in python (pyFAI) and MATLAB (FMINUIT and Global Optimization Toolbox) to conduct data processing and analysis. We used Gaussian 16, Openmolcas 8.6, and ORCA 5.0.4 for ab-initio and DFT calculations. We used ChemRate 1.5.10 for RRKM calculations. We used MOLDY 2.16e for molecular dynamics simulations.

For manuscripts utilizing custom algorithms or software that are central to the research but not yet described in published literature, software must be made available to editors and reviewers. We strongly encourage code deposition in a community repository (e.g. GitHub). See the Nature Portfolio guidelines for submitting code & software for further information.

Data

Policy information about availability of data

All manuscripts must include a data availability statement. This statement should provide the following information, where applicable:

- Accession codes, unique identifiers, or web links for publicly available datasets
- A description of any restrictions on data availability
- For clinical datasets or third party data, please ensure that the statement adheres to our policy

All data supporting the findings of this study are available in the article and supplementary information and from the authors upon reasonable request.

Research involving human participants, their data, or biological material

Policy information about studies with human participants or human data. See also policy information about sex, gender (identity/presentation), and sexual orientation and race, ethnicity and racism.

Reporting on sex and gender	This manuscript does not contain reports on sex or gender.
Reporting on race, ethnicity, or other socially relevant groupings	This manuscript does not contain reports on race, ethnicity, or other socially relevant groupings.
Population characteristics	This manuscript does not contain reports on social studies that require the assessment of population characteristics.
Recruitment	This manuscript does not contain reports on social studies that require the assessment of recruitment processes
Ethics oversight	This manuscript does not contain reports on social studies that require the assessment of ethical protocols.

Note that full information on the approval of the study protocol must also be provided in the manuscript.

Field-specific reporting

Please select the one below that is the best fit for your research. If you are not sure, read the appropriate sections before making your selection.

[x] Life sciences [ ] Behavioural & social sciences [ ] Ecological, evolutionary & environmental sciences

For a reference copy of the document with all sections, see nature.com/documents/nr-reporting-summary-flat.pdf

Life sciences study design

All studies must disclose on these points even when the disclosure is negative.

Sample size	The sample size was determined by counting the total number of scattering images, approximately 8,500 images per time delay. This sample size is sufficient to ensure the credibility of the analysis, as evidenced by the small standard errors of the fitted parameters.
Data exclusions	We compared the area-under-the-curve (AUC) values of individual difference scattering curves and excluded those that deviated significantly from the others during the averaging process. The AUC is a widely accepted criterion in the field of TRXL data processing for determining the quality of each curve.
Replication	We confirmed the data reproducibility by comparing the averaged curves from the entire dataset, consisting of approximately 8,500 images per time delay, with those from individual runs, each containing approximately 1,000 images per time delay. Additionally, we verified the reproducibility of the TRXL curve at 100 ps from this study by comparing it with the curve measured and published elsewhere.
Randomization	Randomization is irrelevant to this study because the experimental TRXL data used in this analysis was obtained by averaging scattering information from repeatedly measured images.
Blinding	Blinding is irrelevant to this study because we did not subjectively group the obtained data and relied solely on objective criteria, such as time delays, which are definitively determined by the physical position of the laser motor.

Reporting for specific materials, systems and methods

We require information from authors about some types of materials, experimental systems and methods used in many studies. Here, indicate whether each material, system or method listed is relevant to your study. If you are not sure if a list item applies to your research, read the appropriate section before selecting a response.

Materials & experimental systems

n/a	Involved in the study	
x		Antibodies
x		Eukaryotic cell lines
x		Palaeontology and archaeology
x		Animals and other organisms
x		Clinical data
x		Dual use research of concern
x		Plants

Methods

n/a	Involved in the study	
x		ChIP-seq
x		Flow cytometry
x		MRI-based neuroimaging

Plants

Seed stocks	Not applicable.
Novel plant genotypes	Not applicable.
Authentication	Not applicable.
